# BCl_3_‐Induced Annulative Oxo‐ and Thioboration for the Formation of C3‐Borylated Benzofurans and Benzothiophenes

**DOI:** 10.1002/anie.201610014

**Published:** 2016-11-29

**Authors:** Andrew J. Warner, Anna Churn, John S. McGough, Michael J. Ingleson

**Affiliations:** ^1^ School of ChemistryUniversity of ManchesterOxford RoadManchesterM13 9PLUK

**Keywords:** annulation, borylation, cross coupling, electrophilic cyclization, organoboranes

## Abstract

BCl_3_‐induced borylative cyclization of aryl‐alkynes possessing ortho‐EMe (E=S, O) groups represents a simple, metal‐free method for the formation of C3‐borylated benzothiophenes and benzofurans. The dichloro(heteroaryl)borane primary products can be protected to form synthetically ubiquitous pinacol boronate esters or used in situ in Suzuki–Miyaura cross couplings to generate 2,3‐disubstituted heteroarenes from simple alkyne precursors in one pot. In a number of cases alkyne trans‐haloboration occurs alongside, or instead of, borylative cyclization and the factors controlling the reaction outcome are determined.

Benzofurans and benzothiophenes are important structures found in pharmaceutical targets (e.g., desketoraloxifene) and organic materials.^1^, ^2^ The boronic acid derivatives of these heteroaromatics are desirable as they are bench‐stable, have low toxicity and are effective in many functional group transformations, including the ubiquitous Suzuki–Miyaura cross coupling reaction.^3^ Typically, the formation of these borylated compounds is achieved via the C−H or C−X borylation of the pre‐formed heteroaromatic.3b, 4 An alternative more efficient approach is to form the heteroaromatic scaffold and the C−B bond in one pot via the borylative cyclization of alkynes. This can be mediated by transition metal catalysts^5^ or in the absence of a metal catalyst by using strong boron electrophiles.^6^ The latter approach was pioneered using B(C_6_F_5_)_3_ which on addition to appropriately substituted alkynes led to a range of borylated heterocycles, including products derived from aminoboration^7^ and oxoboration (Scheme 1).^8^ Other catalyst‐free cyclitive elemento‐borations have been reported, albeit to a lesser extent,^5^, ^6^ with reports of cyclitive thioboration particularly rare.^9^

**Scheme 1:**
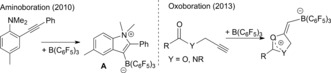
Borylative cyclization of substituted alkynes with B(C_6_F_5_)_3_.

Whilst B(C_6_F_5_)_3_ was crucial in developing metal‐free alkyne borylative cyclization it leads to zwitterionic products such as **A** (Scheme 1). The use of these species in subsequent functional group transformations is not established, currently limiting their synthetic utility.^10^ Using alternative boron Lewis acids such as BCl_3_ to effect borylative cyclization enables the formation of organo‐boronic acid derivatives on work‐up,^11^ and consequentially access to the myriad of already proven transformations. However, this is an underdeveloped approach with demonstrated, modular protocols scarce. Two notable exceptions are 1) the BCl_3_‐induced alkyne borylative cyclization where a (hetero)aromatic moiety is the nucleophile attacking the BCl_3_‐activated alkyne (Scheme 2, top left),^12^ and 2) the use of *B*‐chloro‐catecholborane to produce borylated lactones via cyclitive alkyne oxoboration (Scheme 2, bottom left).^13^ Both protocols generate desirable boronic acid derivatives on (trans)esterification, and are complementary to electrophilic iodinative cyclization (which generates organic electrophiles).^14^

**Scheme 2:**
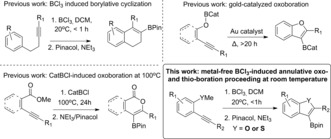
Previous relevant borylative cyclization reactions and this work.

From these studies key requirements enabling borylative cyclization without metal catalysts can be identified, including that the boron electrophile must: a) bind reversibly to the heteroatomic moiety, and b) induce borylative cyclization preferentially to dealkylation reactions (e.g., cyclization occurs prior to O−R cleavage). Guided by these herein we report our studies into the reaction of BCl_3_ with 2‐alkynyl‐anilines, anisoles and thioanisoles, which led to the development of a simple new route to important boronic acid derivatives of benzothiophenes and benzofurans. This route (Scheme 1, bottom right) is catalyst‐free and thus distinct to a recent cyclitive alkyne oxo‐boration report which required Au catalysts (Scheme 1, top right).^15^

Our studies commenced by combining equimolar BCl_3_ and *N*,*N*‐dimethyl‐2‐(phenylethynyl)aniline (**1**) for comparison with B(C_6_F_5_)_3_ which formed zwitterion **A**.^7^ In contrast to B(C_6_F_5_)_3_ addition of BCl_3_ did not lead to a borylated indole with X‐ray diffraction studies revealing it had instead formed **2** (Figure 1), the product from alkyne *trans*‐haloboration. The reactivity disparity between BCl_3_ and B(C_6_F_5_)_3_ is attributed to stronger N→B coordination with BCl_3_ due to the lower steric crowding around boron. Notably, **2** is not the expected product from the direct haloboration of an alkyne with BCl_3_, which would proceed by *syn*‐addition of Cl_2_B−Cl,^16^ suggesting **2** is formed by a different mechanism. Precedence for alkyne *trans*‐haloboration is extremely limited, with compound **C** (Figure 1), the *trans*‐haloboration/demethylation product from the addition of BBr_3_ to *o*‐alkynyl‐anisole **B** a notable exception.^17^ With direct haloboration precluded it is possible that the reaction proceeds from the (*N*,*N*‐Me_2_‐aniline)–BCl_3_ adduct by chloride transfer from boron to carbon, related to that calculated for intramolecular alkyne *trans*‐hydroboration.^18^

**Figure 1:**
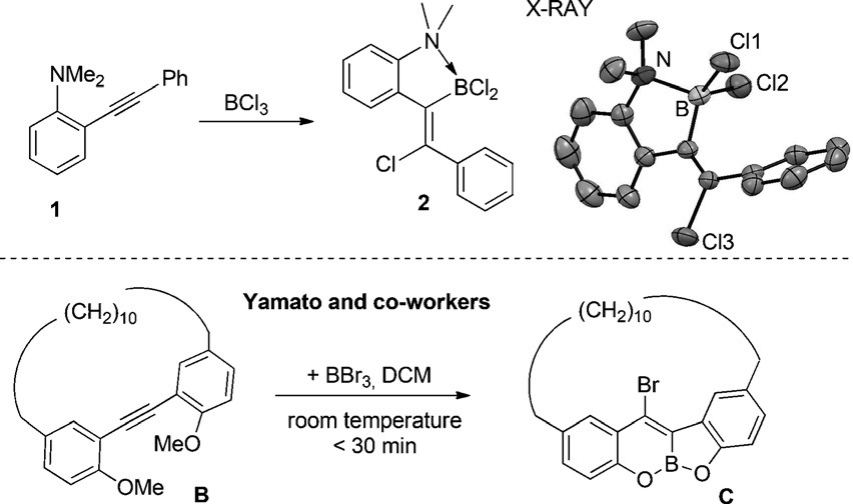
*Trans*‐haloboration of **1** with BCl_3_. Top right, solid state structure of **2**, thermal ellipsoids at 50 % probability and hydrogens omitted for clarity. Bottom, a previously reported alkyne *trans*‐haloboration.

With the formation of C3‐borylated indoles disfavored under these conditions due to *trans*‐haloboration the propensity of *o*‐alkynyl anisoles to undergo borylative cyclization was explored. The rapid formation of **C** from **B** clearly indicates that *trans*‐haloboration also is viable with *o*‐alkynyl‐anisoles, however, this reaction was proposed to proceed via initial ether demethylation then haloboration.^17^ While ether cleavage of anisoles with BBr_3_ is well documented, detailed studies into the mechanism are rare,^19^ but one recent report calculated that PhO−Me cleavage is a bimolecular process involving two Me(Ph)O−BBr_3_ moieties.19a Thus **B** may be prearranged to undergo rapid ether cleavage and other *o*‐alkynyl anisoles may be less prone to ether cleavage, particularly using BCl_3_ instead of BBr_3_. Consistent with this the combination of equimolar anisole and BCl_3_ in DCM at 20 °C resulted in the formation of a single ^11^B resonance at 32 ppm with minimal ether cleavage observed even after 30 h at 20 °C (only ca. 2.5 % CH_3_Cl was formed by ^1^H NMR spectroscopy). The 32 ppm ^11^B chemical shift is consistent with an equilibrium between the Lewis adduct and free BCl_3_ and anisole. Thus anisole binding to BCl_3_ is reversible and ether cleavage is not significant at 20 °C, suggesting that alkyne borylative cyclization using BCl_3_ is viable.

1‐Methoxy‐2‐(phenylethynyl)benzene (**3 a**) was cyclized in DCM using BCl_3_ (Scheme 3). The reaction was rapid (<5 min at 20 °C), as indicated by the consumption of **3 a** along with the generation of CH_3_Cl (*δ*_1H_ 3.02 ppm) and a new major resonance centered at 51 ppm in the ^11^B NMR spectrum, consistent with a heteroaryl‐BCl_2_ species. A minor broad resonance at 14.2 ppm in the ^11^B NMR spectrum was also observed. Esterification with pinacol/NEt_3_ enabled the isolation of **4 a** in 56 % yield without column chromatography. No intermediates are observed so detailed discussion of the mechanism is not warranted, although alkyne activation by BCl_3_ and cyclization presumably occurs prior to demethylation based on the slow ether cleavage observed on combining anisole and BCl_3_. It is noteworthy that a non‐linked analogue of **B**, 1,2‐bis(2‐methoxyphenyl)ethyne, undergoes rapid *trans*‐haloboration and demethylation with both BCl_3_ and BBr_3_, thus the reactivity disparity between **3 a** and **B** is not due to the use of different boron trihalides.

**Scheme 3:**
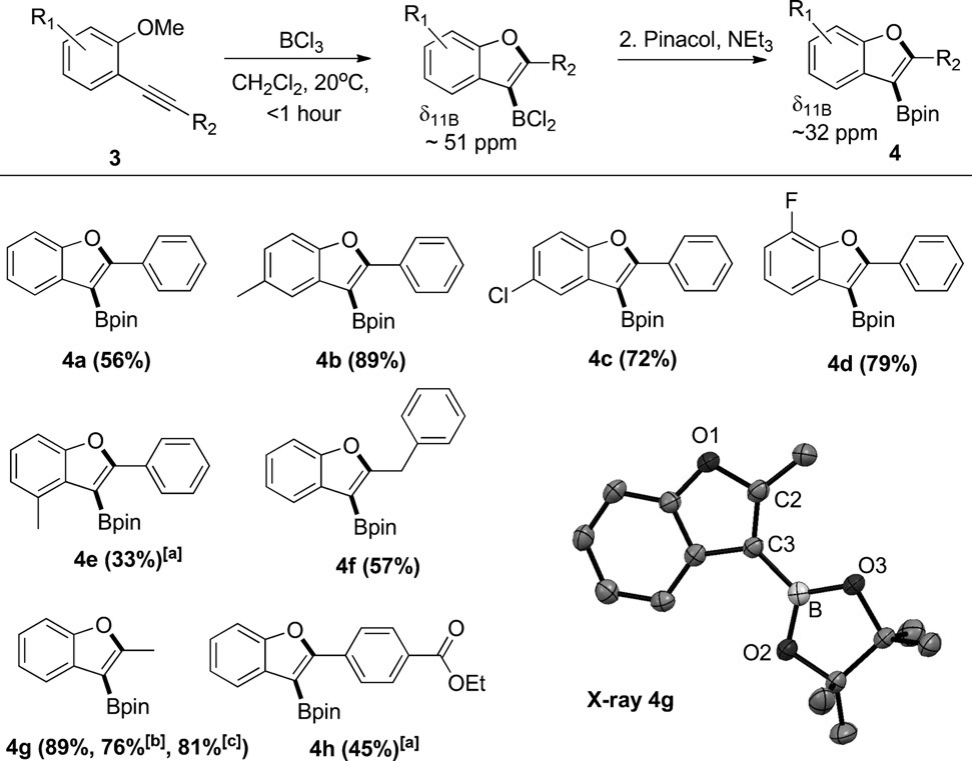
BCl_3_‐induced borylative cyclization of 2‐alkynyl‐anisoles. Bottom right, solid state structure of **4 g**, thermal ellipsoids at 50 % probability and hydrogens omitted for clarity. [a] 12 h. [b] 6 mmol scale to produce 1.16 g of **4 g**. [c] Using non‐purified solvents under ambient atmosphere.

Exploration of the substrate scope revealed that electron‐donating and ‐withdrawing groups on the anisole ring are compatible in certain positions (**4 b**–**e**). Furthermore, borylative cyclization is not limited to diarylalkynes with benzyl‐ and methyl‐substituted alkynes converted to the benzofurans **4 f** and **4 g** in good yield, with the structure of **4 g** confirmed by X‐ray crystallography. **4 g** was also accessible on a gram scale and using non‐purified solvents under ambient conditions in good yield. Whilst a phenyl group substituted with an electron‐withdrawing group *para* to the alkyne led to the borylated benzofuran in moderate isolated yield (**4 h**), when ester and nitro groups were incorporated into the anisole ring *para* to the alkyne this led to low conversions to the benzofuran‐BCl_2_ species (the *δ*_11B_ 51 ppm is the minor component). Instead a *δ*_11B_ 15 ppm resonance was the major product with **3 i** (Scheme 4), whilst for **3 j** (Scheme 4), where a naphthyl group has been incorporated resulting in an increase in the steric environment around the alkyne, the major *δ*_11B_ resonance is centered at 14 ppm. With both these substrates after the addition of BCl_3_ the ^1^H NMR spectra revealed that minimal CH_3_Cl had formed (consistent with *δ*_11B_ 51 ppm being a minor resonance). Instead a singlet was observed at 4.56 and 4.49 ppm, respectively from **3 i** and **3 j**, more consistent with an intact ArylOMe unit coordinated to a Lewis acid. Attempts to isolate the product derived from **3 i** after esterification with Et_3_N/pinacol led to isolation of the starting alkyne, presumably due to E2 elimination. The naphthyl derivative **5** was formed as the major product post esterification, with ^1^H, ^13^C{^1^H}, ^11^B NMR spectroscopy fully consistent with haloboration, a formulation supported by mass spectroscopy. Therefore to form borylated benzofurans in acceptable isolated yields by BCl_3_‐induced borylative cyclization significant bulk around the alkyne and strong EWG in the *para* position (to the alkyne) of the anisole moiety have to be avoided.

**Scheme 4:**
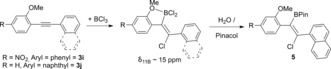
*Trans*‐haloboration with strong electron‐withdrawing/bulky groups.

With the substituent effects probed the functional group tolerance of BCl_3_‐induced borylative cyclization was further explored using the “robustness screen” methodology;^20^ specifically, monitoring the cyclization of **3 b** in the presence of various additives. This revealed that borylative cyclization was not affected by additives containing nitro, vinyl or CF_3_ groups (in each case >80 % of the borylated benzofuran was formed with the additive not consumed). However, benzaldehyde and acetone were not compatible, with the addition of BCl_3_ to separate reactions containing these additives and **3 b** leading to additive consumption and significantly reduced benzofuran formation. Other Lewis basic groups were compatible with borylative cyclization provided that >2 equivalents of BCl_3_ was used, with the first equivalent of BCl_3_ coordinating to the Lewis basic group (in each case >70 % conversion to the borylated benzofuran was observed in the presence of a tertiary amine, a tertiary amide, a pyridine and a nitrile). Established routes to 3‐borylated‐2‐organo‐benzofurans generally proceed from 3‐halo‐2‐organo‐benzofurans by metallation/quenching with B(OR)_3_, or by Pd‐catalyzed Miyaura borylation.3b Notably these routes are not compatible with some of the functional groups tolerated by BCl_3_‐induced borylative cyclization (e.g., amide/nitrile groups are generally incompatible with metallation). Furthermore, this methodology is complementary to iridium‐catalyzed C−H borylation which provides C2‐ or C7‐borylated benzofurans.^4^ Finally, it worth emphasizing that **4 a**–**h** are formed at ambient temperature without a catalyst using inexpensive BCl_3_, in contrast the previous borylative cyclization route to C3‐borylated benzofurans required pre‐installation of the borane (using NaH/CatBCl), Au catalysis, raised temperatures and ≥20 h.^15^

Multiple borylative cyclizations also proceed with appropriately substituted diynes, with **6** converted to **7**, a diborylated diaryl‐benzo[1,2‐b:4,5‐b′]difuran, in excellent yield using BCl_3_ (Scheme 5). **7** represents a versatile precursor to 2,3,6,7‐tetraarylbenzo[1,2‐b:4,5‐b′]difurans which are of interest as hole transport materials.^2^ To the best of our knowledge 3,7‐diborylated benzodifurans have not been previously reported.

**Scheme 5:**
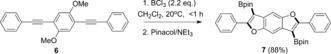
Double BCl_3_‐induced borylative cyclization.

While the purified borylated benzofurans reported herein are effective in Suzuki–Miyaura cross couplings (e.g., **4 g** with 4‐bromo‐toluene) to enhance the utility of this methodology a one‐pot borylative cyclization/Suzuki–Miyaura cross coupling procedure was developed (Scheme 6). This does not require isolation of the borylated benzofuran, instead the benzofuran‐BCl_2_ product is hydrolyzed in situ to the boronic acid and then subjected to conventional Suzuki–Miyaura cross coupling conditions. This one‐pot procedure is a simple and rapid way to generate 2,3‐disubstituted benzofurans from simple alkynyl precursors in good yield (72 % isolated yield of **8**).

**Scheme 6:**
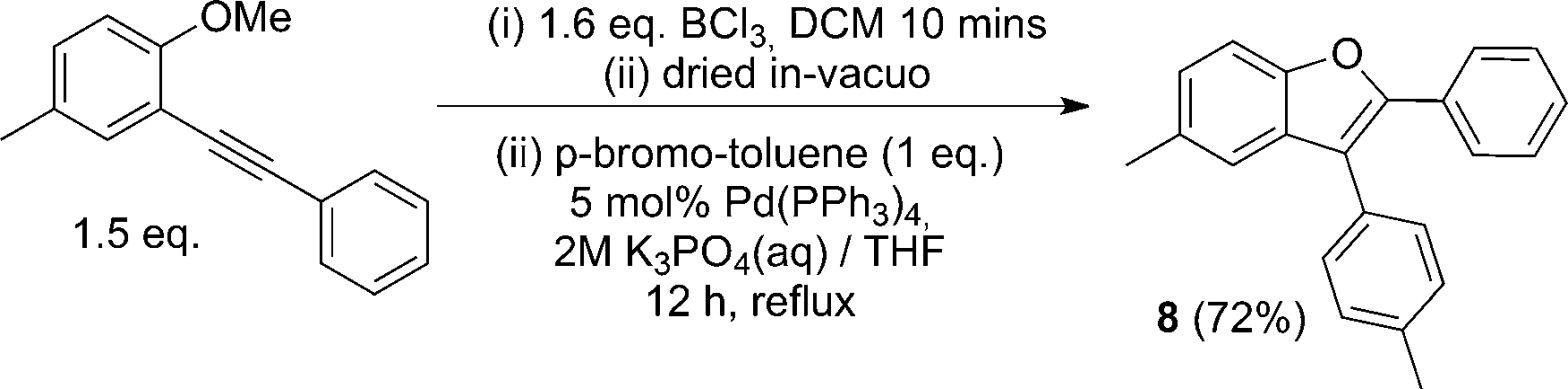
One pot borylative cyclization and Suzuki–Miyaura cross coupling.

*o*‐Alkynyl‐thioanisoles and BCl_3_ were explored next to assess if BCl_3_ induced borylative cyclization was possible via alkyne thio‐boration. Firstly, equimolar thioanisole and BCl_3_ were combined which led to a species with *δ*_11B_ 7.9 ppm, indicating significant adduct formation, but importantly no S−Me cleavage was observed. Furthermore, previous work has shown that thioanisole‐(BH_*x*_Cl_3−*x*_) (*x*=1 or 2) compounds are effective hydroborating agents at 20 °C indicating that an electrophilic borane is accessible from these Lewis adducts.^21^ Therefore BCl_3_ was added to methyl(2‐(phenylethynyl)‐phenyl)sulfane (**9 a**) in DCM with in situ ^11^B NMR spectroscopy revealing one major product had formed with a broad resonance centered at 4 ppm, which does not correspond to a 3‐BCl_2_‐benzothiophene species (expected *δ*_11B_ ca. 52 ppm).^22^ This is consistent with no chloromethane being observed in the ^1^H NMR spectrum. Methylsulfonium cations are significantly weaker methylating agents (less prone to Me^+^ transfer to nucleophiles) than methyloxonium cations,^23^ therefore we surmised that the major compound is the zwitterion **10 a** analogous to **A** (Scheme 7). In our hands crystalline material of **10** could not be isolated therefore support for this assignment was provided by combining **9 a** with BCl_3_ (to form **10 a**) and then adding Et_3_N as a stronger nucleophile to induce demethylation. This led to formation of [Et_3_NMe]^+^ (by ^1^H NMR spectroscopy) and a new major broad ^11^B resonance at 6.3 ppm attributed to the product from demethylation of **10 a** by Et_3_N. On addition of one equivalent of AlCl_3_ this compound was then converted to a new major species displaying a broad ^11^B resonance at 52.9 ppm fully consistent with a benzothiophene‐BCl_2_ compound.^22^ The same boron species is formed by initial addition of AlCl_3_ to **10 a** followed by Et_3_N. Esterification of the *δ*_11B_ 52.9 ppm species with excess pinacol/Et_3_N led to the desired product **11 a** in good isolated yield (68 %), unequivocally confirming that borylative cyclization has taken place. This reaction is notable as a rare example of cyclitive alkyne thioboration.9c It should be noted that attempts to directly esterify the zwitterion **10 a** led to significantly lower isolated yields of **11 a** (38 %). This is attributed to **10 a** having a greater propensity to undergo protodeboronation due to the more nucleophilic anionic benzothienyl‐BCl_3_ moiety (relative to benzothienyl‐BCl_2_).

**Scheme 7:**
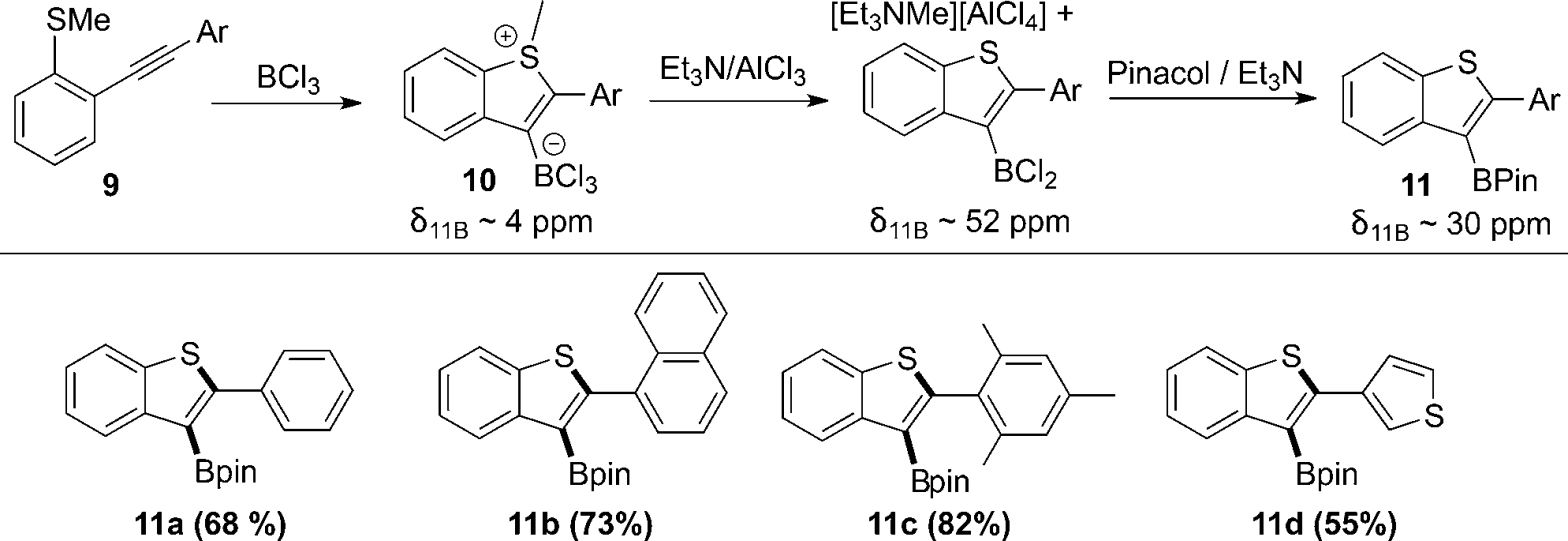
Borylative cyclization followed by demethylation/dehalogenation to form benzothienyl‐BCl_2_ and then subsequent esterification.

With the functional group tolerance already assessed in benzofuran formation other thioanisole substrates were selected to assess if alkyne haloboration was a competitive pathway. As there was no evidence (in situ or post work‐up) for haloboration with **9 a** bulkier substituents, naphthyl and mesityl, **9 b** and **9 c**, respectively, were incorporated into the alkyne. Addition of BCl_3_ to these alkynes resulted in similar outcomes to that observed with **9 a** with no evidence for haloboration in either case, suggesting it is not a competitive reaction with thioanisoles. Again, the isolated yield of the benzothiophene pinacol boronate ester is higher on addition of Et_3_N/AlCl_3_ prior to esterification (e.g., for producing **11 b** yield=48 % direct from the zwitterion **10 b** whereas it is 73 % on esterification after addition of Et_3_N/AlCl_3_). To demonstrate further that this methodology allows access to otherwise challenging to synthesize boronic acid derivatives **11 d** was produced in 55 % isolated yield. Compound **11 d** is not readily accessible by established borylation routes commencing from 2‐(thiophen‐3‐yl)benzo[b]thiophene (e.g., Ir‐catalyzed borylation and halogenation/lithiation approaches would all proceed at the thienyl alpha position).3b, 4

In conclusion, two distinct reaction pathways operate on addition of BCl_3_ to arylalkynes possessing *ortho* E−Me (E=NMe, O or S) moieties, specifically borylative cyclization and *trans*‐haloboration. The latter occurs with *N*,*N*‐dimethyl‐2‐(phenylethynyl)aniline whilst all the *o*‐alkynyl‐thioanisoles studied react selectively by borylative cyclization. For *o*‐alkynyl‐anisoles both pathways are observed, with borylative cyclization dominating provided strong electron‐withdrawing groups on the anisole moiety *para* to the alkyne, or significant steric bulk are absent. This methodology is a simple, scalable and metal‐free route to useful benzofuran and benzothiophene boronic acid derivatives, many of which would be challenging to access by other established borylation methodologies.

## Conflict of interest

The authors declare no conflict of interest.

## Supporting information

As a service to our authors and readers, this journal provides supporting information supplied by the authors. Such materials are peer reviewed and may be re‐organized for online delivery, but are not copy‐edited or typeset. Technical support issues arising from supporting information (other than missing files) should be addressed to the authors.

Supplementary
