# Supplementary material for: BCl3‐Induced Annulative Oxo‐ and Thioboration for the Formation of C3‐Borylated Benzofurans and Benzothiophenes
Source: Angew Chem Int Ed Engl. 2016 Nov 29;56(1):354–8. doi: 10.1002/anie.201610014 (PMC5396270; doi:10.1002/anie.201610014)
Supplement: Supplementary file 1 — Supplementary [file ANIE-56-354-s001.pdf]

Angewandte Chemie
Eine Zeitschrift der Gesellschaft Deutscher Chemiker

Supporting Information

BCl_3-Induced Annulative Oxo- and Thioboration for the Formation of C3-Borylated Benzofurans and Benzothiophenes

*Andrew J. Warner, Anna Churn, John S. McGough, and Michael J. Ingleson**

anie_201610014_sm_miscellaneous_information.pdf

Supporting Information

1. *General Considerations*
2. *N-Directed Trans-haloboration*
3. *Synthesis of Alkynes*
4. *Cyclisation of 2-alkynylanisoles*
5. *O-Directed Trans-haloboration*
6. *External Additive Screening*
7. *Double Borylative Cyclisation*
8. *In-situ Cross-coupling*
9. *Cyclisation of 2-alkynylthioanisoles*
10. *In-situ NMR spectra*
11. *Spectral Data on isolated compounds*
12. *Crystal Data*
13. *References*

1. General Considerations

All manipulations of air and moisture sensitive species were performed under an atmosphere of argon or nitrogen using standard Schlenk and glovebox techniques unless otherwise stated. Glassware was dried in a hot oven overnight and heated under vacuum before use. Triethylamine was dried over calcium hydride and distilled under vacuum. Pentane and dichloromethane were dried by passing through an alumina drying column incorporated into an MBraun SPS800 solvent purification system. Tetrahydrofuran was dried by refluxing over potassium metal. All solvents were degassed and stored over molecular sieves (3Å) under inert atmosphere. All other materials were purchased from commercial vendors and used as received. NMR spectra were recorded with a Bruker AV-400 spectrometer (400 MHz ^{1}H; 100 MHz ^{13}C; 128 MHz ^{11}B; 376.50 MHz ^{19}F; 104 MHz ^{27}Al). ^{1}H NMR chemical shifts are reported in ppm relative to protio impurities in the deuterated solvents and ^{13}C NMR using the solvent resonances unless otherwise stated. ^{11}B NMR spectra were referenced to external BF_3:Et_2O, ^{27}Al to $Al(NO_3)_2$ in D_2O ($Al(D_2O)_6^{3+}$) and ^{19}F to external C_6F_6. *Resonances for the carbon directly bonded to boron are not observed in the $^{13}C\{^{1}H\}$ NMR spectra due to quadrupolar relaxation effects. Some carbon signals in compounds **4a, 4b, 4h and 8** are not observed due to coincident peaks.* The ionisation mode used in GC-MS was electron ionisation. All small scale cyclization reactions were carried out in J. Young NMR tubes to facilitate in-situ reaction monitoring. A number of samples are analysed in-situ in protio solvent with a capillary insert containing wet deuterated d_6-DMSO, this leads to a residual H_2O resonance being observed at 3.95 ppm in the ^{1}H NMR spectra.

2. *N-Directed Transhaloboration*

Nitrogen Directed (**2**)

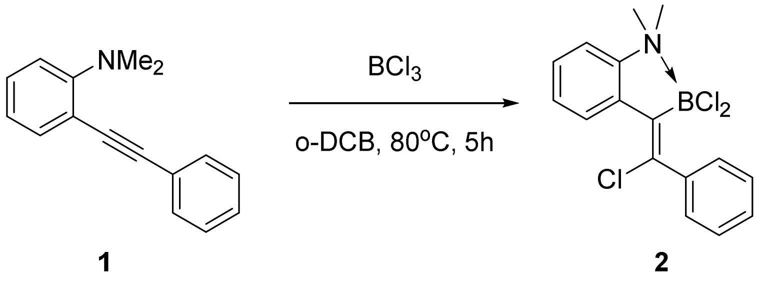

In a Schlenk fitted with J. Young's valve, alkyne **1** (50 mg, 0.226 mmol, 1 eq.) was dissolved in o-dichlorobenzene (2 mL) and BCl_3 (1M in heptane) (0.29 mL, 0.290 mmol, 1.3 eq.) was added. The reaction was heated at 80^oC for 5 hours then allowed to cool to 20^oC. The solution was layered with hexane and stored in the freezer for a week. A colourless crystalline solid deposited on the walls of the ampoule and was collected. Upon analysis, the solid was found to be the desired *trans*-haloboration product **2** (51 mg, 67%). ^1H NMR (400 MHz, CD_2Cl_2): δ 3.02 (6H, s, NMe_2); 7.35-7.41 (4H, m, Ar-*H*); 7.44 (1H, t, J = 7.6 Hz, Ar-*H*); 7.50 (1H, t, J = 7.6 Hz, Ar-*H*); 7.63-7.70 (2H, m, Ar-*H*); 8.47 (1H, d, J = 7.8 Hz, Ar-*H*); $^{13}C\{^1H\}$ NMR (100.6 MHz, CD_2Cl_2): δ 49.5, 117.0, 127.4, 128.1, 128.7, 128.9, 129.0, 129.5, 130.5, 136.0, 140.5, 149.1; ^{11}B NMR (128.6MHz, CD_2Cl_2); δ 10.5 (s) [GC-MS] *m/z* calculated for $C_{16}H_{15}BCl_3N$, 337.0; found 337.0. GC-MS retention times of analytes: 14.30 minutes: N-directed *trans*-haloboration product; [Acc. Mass] Calculated $[M]^+$ 337.0363 $gmol^{-1}$, Observed $[M]^+$ 337.0352 $gmol^{-1}$; [Elem. Analysis] Calc: C (56.78%); H (4.47%); N (4.14%); Found: C (56.79%); H (4.56%); N (4.11%)

3. Synthesis of Alkynes

General Procedure A[1]

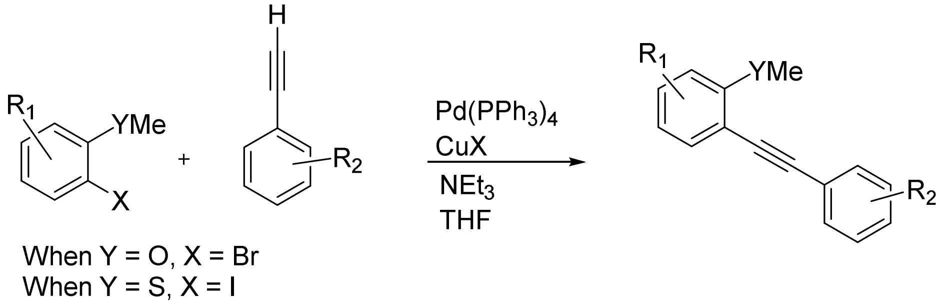

A Schlenk fitted with a J. Young's valve was charged with *tetrakis*-(triphenylphosphine)palladium(0) (0.02 eq.) and copper(I) halide (0.04 eq.). Tetrahydrofuran was added and the suspension was stirred for five minutes, followed by the addition of the aryl bromide (1 eq.) and triethylamine. After five minutes more stirring, the alkyne (1.1 eq.) was added and the solution was stirred and refluxed in the sealed vessel for 12 h. The solution was cooled to room temperature, filtered through a layer of celite on top of silica and eluted with diethyl ether (3 x 10 mL). The solvent was removed *in vacuo* and the crude material was purified via column chromatography to yield the corresponding alkyne.

General Procedure B[1]

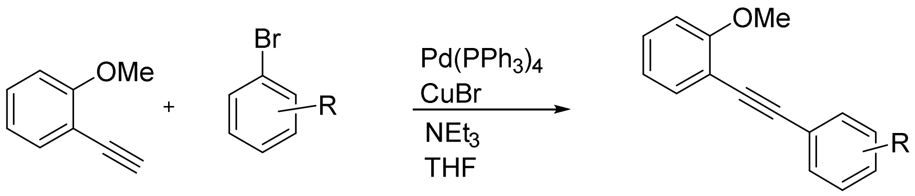

A Schlenk fitted with a J. Young's valve was charged with *tetrakis*-(triphenylphosphine)palladium(0) (0.02 eq.) and copper(I) bromide (0.04 eq.). Tetrahydrofuran was added and the suspension was stirred for five minutes, followed by the addition of the aryl bromide (1 eq.) and triethylamine. After five minutes more stirring, the alkyne (1.1 eq.) was added and the solution was stirred and refluxed in the sealed vessel for 12 h. The solution was cooled to room temperature, filtered through a layer of celite on top of silica and eluted with diethyl ether (3 x 10 mL). The solvent was removed *in vacuo* and the crude material was purified via column chromatography to yield the corresponding alkyne.

N,N-dimethyl-2-(phenylethynyl)aniline **(1)**

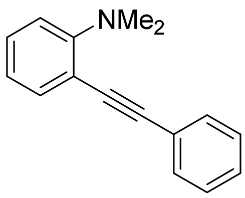

Prepared according to general procedure A. 2-bromo-N,N-dimethylaniline (0.8 g, 4.00mmol, 1 eq.), phenylacetylene (0.66 mL, 6.00 mmol, 1.5 eq), $Pd(PPh_3)_4$ (139 mg, 0.120 mmol, 0.03 eq.), copper(I) bromide (34 mg, 0.240 mmol, 0.06 eq.), triethylamine (8 mL) and THF (10 mL). *Column chromatography eluent: 20% DCM in 40-60 petroleum ether*. **1** (0.42 g, 47%) obtained as an orange oil. Data is in accordance with the literature.[2]

<u>1-methoxy-2-(phenylethynyl)benzene **(3a)**</u>

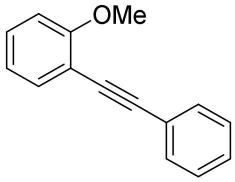

Prepared according to general procedure A. 2-bromoanisole (0.67 mL, 5.35 mmol, 1 eq.), phenylacetylene (0.70 mL, 6.42 mmol, 1.2 eq.), $Pd(PPh_3)_4$ (124 mg, 0.107 mmol, 0.02 eq.), copper(I) bromide (31 mg, 0.214 mmol, 0.04 eq.), triethylamine (8 mL) and THF (10 mL). *Column chromatography eluent: 20% DCM in 40-60 petroleum ether*. **3a** (0.93 g, 84%) obtained as a dark orange oil. The data was in accordance with the literature[3]

<u>1-methoxy-4-methyl-2-(phenylethynyl)benzene **(3b)**</u>

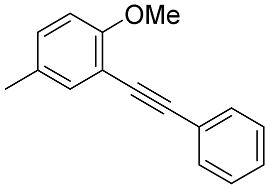

Prepared according to general procedure A. 2-bromo-1-methoxy-4-methylbenzene (1.08 mL, 7.46 mmol, 1 eq.), phenylacetylene (1.00 mL, 8.95 mmol, 1.2 eq.), $Pd(PPh_3)_4$ (172 mg, 0.150 mmol, 0.02 eq.), copper(I) bromide (43 mg, 0.300 mmol, 0.04 eq.), triethylamine (8 mL) and THF (10 mL). *Column chromatography eluent: 20% DCM in 40-60 petroleum ether*. **3b** (1.28 g, 77%) obtained as an orange oil. The data was in accordance with the literature[4]

4-chloro-1-methoxy-2-(phenylethynyl)benzene (**3c**)

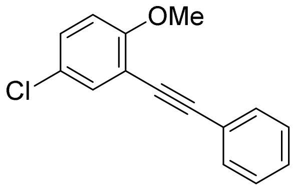

Prepared according to general procedure A. 2-bromo-1-methoxy-4-chlorobenzene (0.92 mL, 6.77 mmol, 1 eq.), phenylacetylene (0.90 mL, 8.13 mmol, 1.2 eq.), $Pd(PPh_3)_4$ (156 mg, 0.135 mmol, 0.02 eq.), copper(I) bromide (39 mg, 0.271 mmol, 0.04 eq.), triethylamine (8 mL) and THF (10 mL). *Column chromatography eluent: 20% DCM in 40-60 petroleum ether.* **3c** (1.62 g, 98%) obtained as an orange oil. ^1H NMR (400 MHz, $CDCl_3$): δ 3.91 (3H, s, OCH_3); 6.83 (1H, d, J = 9.1 Hz, Ar-*H*); 7.26 (1H, dd, J = 8.8 Hz, 2.5 Hz, Ar-*H*); 7.33-7.39 (3H, m, Ar-*H*); 7.48 (1H, d, J = 2.5 Hz, Ar-*H*); 7.54-7.58 (2H, m, Ar-*H*); $^{13}C\{^1H\}$ NMR (100.6 MHz, $CDCl_3$): δ 56.2, 84.4, 94.5, 111.8, 114.1, 123.1, 125.2, 128.3, 128.5, 129.5, 131.7, 132.9, 158.6; [GC-MS] *m/z* calculated for $C_{15}H_{11}ClO$, 242.1; found 242.1. GC-MS retention times of analytes: 11.96 minutes: 4-chloro-1-methoxy-2-(phenylethynyl)benzene; [Acc. Mass] Calculated $[M+H]^+$: 243.0571 $gmol^{-1}$, Observed: $[M+H]^+$ 243.0571 $gmol^{-1}$.

1-(phenylethynyl)-2-methoxy-3-fluorobenzene **(3d)**

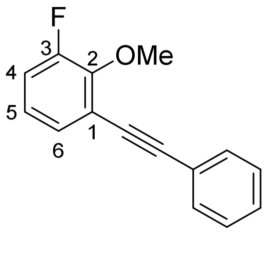

Prepared according to general procedure A. 1-bromo-2-methoxy-3-fluorobenzene (0.65 mL, 4.88 mmol, 1 eq.), phenylacetylene (0.54 mL, 4.88 mmol, 1.2 eq.), $Pd(PPh_3)_4$ (113 mg, 0.098 mmol, 0.02 eq.), copper(I) bromide (28 mg, 0.196 mmol, 0.04 eq.), triethylamine (8 mL) and THF (10 mL). *Column chromatography eluent: 25% DCM in 40-60 petroleum ether.* **3d** (0.77 g, 71%) obtained as a brown solid. ^1H NMR (400 MHz, $CDCl_3$): δ 4.09 (3H, s, OCH_3); 6.96-7.03 (1H, m, Ar-H); 7.05-7.12 (1H, m, Ar-*H*); 7.28 (1H, dt, J = 7.8 Hz, 1.5 Hz, Ar-*H*); 7.34-7.41 (3H, m, Ar-*H*); 7.54-7.60 (2H, m, Ar-*H*); $^{13}C\{^1H\}$ NMR (100.6 MHz, $CDCl_3$): δ 61.6 (d, $^4J_{CF}$ = 4.4 Hz, O*Me*); 84.6 (d, $^4J_{CF}$ = 5.2 Hz, C_1-*C*≡C); 94.3 (C_1-C≡*C*); 117.2 (d, $^2J_{CF}$ = 19.2 Hz, C_4);

118.7 (d, $^3J_{CF}$ = 3.7 Hz, C_1); 123.1, 123.4 (d, $^2J_{CF}$ = 19.2 Hz, C_4); 128.4, 128.56, 128.62 (d, $^4J_{CF}$ = 3.7 Hz, C_6); 131.7, 148.6 (d, $^2J_{CF}$ = 11.8 Hz, C_2); 155.4 (d, $^1J_{CF}$ = 247.0 Hz, C_3); ^{19}F NMR (376.50 MHz, $CDCl_3$): δ -130.79 (1F, Ar-*F*); [GC-MS] *m/z* calculated for $C_{15}H_{11F}O$, 226.1; found 226.1. GC-MS retention times of analytes: 10.92 minutes: 1-(phenylethynyl)-2-methoxy-3-fluorobenzene; [Acc. Mass] Calculated $[M+H]^+$: 227.0867 $gmol^{-1}$, Observed: $[M+H]^+$ 227.0867 $gmol^{-1}$.

<u>1-methoxy-3-methyl-2-(phenylethynyl)benzene **(3e)**</u>

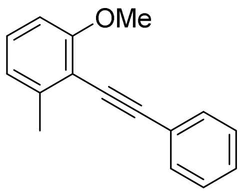

Prepared according to general procedure A. 2-bromo-1-methoxy-3-methylbenzene (1 g, 4.97 mmol, 1 eq.), phenylacetylene (0.55 mL, 4.97 mmol, 1 eq.), $Pd(PPh_3)_4$ (115 mg, 0.099 mmol, 0.02 eq.), copper(I) bromide (28 mg, 0.198 mmol, 0.04 eq.), triethylamine (8 mL) and THF (10 mL). *Column chromatography eluent: 30% DCM in 40-60 petroleum ether*. **3e** (0.22 g, 19%) obtained as a brown oil. Data is in accordance with the literature.[6]

<u>1-methoxy-2-(3-phenylprop-1-yn-1-yl)benzene **(3f)**</u>

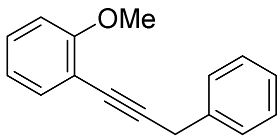

A Young's ampoule was charged with *tetrakis*-(triphenylphosphine)palladium(0) (247 mg, 0.214 mmol, 0.02 eq.) and copper(I) bromide (61 mg, 0.428 mmol, 0.04 eq.). Tetrahydrofuran (10 mL) was added and the suspension was stirred for five minutes, followed by the addition of 2-bromoanisole (1.33 mL, 10.69 mmol, 1 eq.) and triethylamine (7 mL). After five minutes more stirring, 3-phenylprop-1-yne (1.6 mL, 12.83 mmol, 1.2 eq.) was added and the solution was stirred and refluxed in the sealed vessel for 12 h. The solution was cooled to room temperature, filtered through a layer of celite on top of silica and eluted with diethyl ether (3 x 10 mL). The solvent was removed *in vacuo* and the crude material was purified via column chromatography (15% DCM in 40-60 petroleum ether) to yield **3f** (0.93 g, 39%) as a brown oil. ^{1}H NMR (400 MHz, $CDCl_3$): δ 3.91 (3H, s, OCH_3); 3.93

(2H, s, CH_2Ph); 6.88-6.94 (2H, m, Ar-*H*); 7.23-7.32 (2H, m, Ar-*H*); 7.36 (2H, t, J = 7.6 Hz, Ar-*H*); 7.42-7.51 (3H, m, Ar-*H*); $^{13}C\{^1H\}$ NMR (100.6 MHz, $CDCl_3$): δ 26.1, 55.8, 78.9, 91.7, 110.5, 112.7, 120.4, 126.6, 128.0, 128.5, 129.3, 133.7, 136.9, 160.0; [GC-MS] *m/z* calculated for $C_{15}H_{11}O$, 207.1; found 207.1. GC-MS retention times of analytes: 10.81 minutes: 1-methoxy-2-(3-phenylprop-1-yn-1-yl)benzene; [Acc. Mass] Calculated $[M]^+$: 222.1045 $gmol^{-1}$, Observed: $[M]^+$ 222.1045 $gmol^{-1}$.

1-methoxy-2-(prop-1-yn-1-yl)benzene **(3g)**

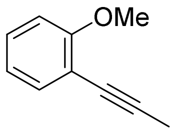

In a Young's ampoule, 2-ethynylanisole (0.98 mL, 7.57 mmol, 1 eq.) was dissolved in tetrahydrofuran and stirred at -78°C over 20 minutes. ^{n}BuLi (5.7 mL, 9.08 mmol, 1.2 eq.) was added dropwise and stirred at -78°C for a further 20 minutes. After this time, iodomethane (0.94 mL, 15.133 mmol, 2 eq.) was added dropwise and the solution was warmed to 20°C and stirred for 5 hours before being quenched with NH_4Cl (100 mL). The desired compound was extracted into DCM (4 x 25 mL), dried over $MgSO_4$ and the solvent was removed *in vacuo* to give **3g** (985 mg, 89%) as an orange oil, with no further purification necessary. Data is in accordance with the literature.[5]

Ethyl 4-((2-methoxyphenyl)ethynyl)benzoate **(3h)**

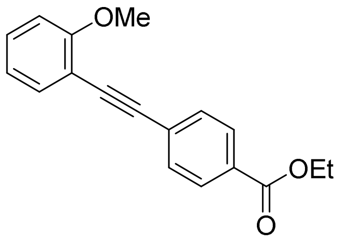

Prepared according to general procedure B. 2-ethynylanisole (0.98 mL, 7.57 mmol, 1.2 eq.), ethyl-4-bromobenzoate (1.03 mL, 6.31 mmol, 1 eq.), $Pd(PPh_3)_4$ (146 mg, 0.126 mmol, 0.02 eq.), copper(I) bromide (36 mg, 0.252 mmol, 0.04 eq.), triethylamine (8 mL) and THF (10

mL). *Column chromatography eluent: 30% DCM in 40-60 petroleum ether*. **3h** (1.33 g, 75%) obtained as an orange oil. Data is in accordance with the literature.[3]

2-methoxy-4-nitro-1-(phenylethynyl)benzene (**3i**)

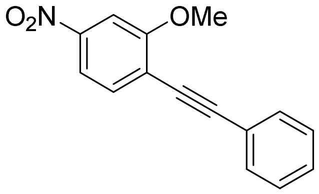

Prepared according to general procedure A. A Schlenk fitted with a J. Young's valve was charged with *tetrakis*-(triphenylphosphine)palladium(0) (122 mg, 0.106 mmol, 0.02 eq.) and copper(I) bromide (30 mg, 0.212 mmol, 0.04 eq.). Tetrahydrofuran (10 mL) was added and the suspension was stirred for five minutes, followed by the addition of 2-bromo-5-nitroanisole (1.23 g, 5.3 mmol, 1 eq.) and triethylamine (7 mL). After five minutes more stirring, phenylacetylene (0.7 mL, 6.36 mmol, 1.2 eq.) was added and the solution was stirred and refluxed in the sealed vessel for 12 h. The solution was cooled to room temperature, filtered through a layer of celite on top of silica and eluted with diethyl ether (3 x 10 mL). The solvent was removed *in vacuo* and the crude material was purified via column chromatography (50% DCM in 40-60 petroleum ether) to yield **3i** (0.91 g, 68%) as a yellow solid. Data is in accordance with the literature.[7]

1-((2-methoxyphenyl)ethynyl)naphthalene **(3j)**

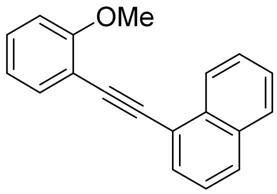

Prepared according to general procedure A. A Schlenk fitted with a J. Young's valve was charged with *tetrakis*-(triphenylphosphine)palladium(0) (124 mg, 0.107 mmol, 0.02 eq.) and copper(I) bromide (31 mg, 0.214 mmol, 0.04 eq.). Tetrahydrofuran (10 mL) was added and the suspension was stirred for five minutes, followed by the addition of 2-bromoanisole (0.67 mL, 5.35 mmol, 1 eq.) and triethylamine (7 mL). After five minutes more stirring, 1-ethynylnaphthalene (0.91 mL, 6.42 mmol, 1.2 eq.) was added and the solution was stirred and refluxed in the sealed vessel for 12 h. The solution was cooled to room temperature, filtered through a layer of celite on top of silica and eluted with diethyl ether (3 x 10 mL). The solvent was removed *in vacuo* and the crude material was purified via column chromatography (50% DCM in 40-60 petroleum ether) to yield **3j** (0.88 g, 63%) as a brown oil. Data is in accordance with the literature.[8]

((2,5-dimethoxy-1,4-phenylene)bis(ethyne-2,1-diyl))dibenzene **(6)**

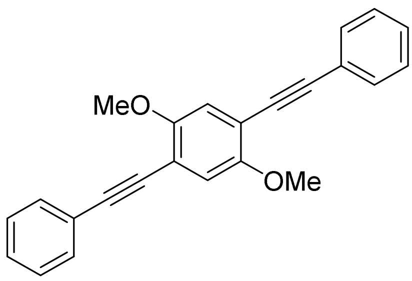

Prepared according to general procedure A. 1,4-dibromo-2,5-dimethoxybenzene (2 g, 6.76 mmol, 1 eq.), phenylacetylene (1.71 mL, 15.55 mmol, 2.3 eq), $Pd(PPh_3)_4$ (312 mg, 0.270mmol, 0.04 eq.), copper(I) bromide (78 mg, 0.541 mmol, 0.08 eq.), triethylamine (10 mL) and THF (15 mL). *Column chromatography eluent: 50% DCM in 40-60 petroleum ether*. **1i** (1.196 g, 52%) obtained as an orange oil. Data is in accordance with the literature.[9]

Methyl(2-(phenylethynyl)phenyl)sulfane **(9a)**

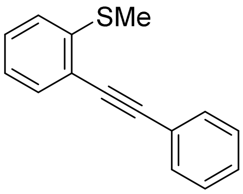

Prepared according to general procedure B. 2-iodothioanisole (1 g, 4.00mmol, 1 eq.), phenylacetylene (0.66 mL, 6.00 mmol, 1.5 eq.), $Pd(PPh_3)_4$ (138 mg, 0.120 mmol, 0.03 eq.), copper(I) iodide (46 mg, 0.24 mmol, 0.06 eq.), triethylamine (8 mL) and THF (10 mL). *Column chromatography eluent: 10% DCM in 40-60 petroleum ether*. **9a** (0.24 g, 27%) obtained as a dark orange oil. Data is in accordance with the literature.[10]

Methyl(2-(naphthalen-1-ylethynyl)phenyl)sulfane **(9b)**

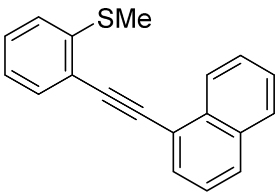

Prepared according to general procedure B. 2-iodothioanisole (1 g, 4.00mmol, 1 eq.), 1-ethynylnaphthalene (0.85 mL, 6.00 mmol, 1.5 eq.), $Pd(PPh_3)_4$ (138 mg, 0.120 mmol, 0.03 eq.), copper(I) iodide (46 mg, 0.24 mmol, 0.06 eq.), triethylamine (8 mL) and THF (10 mL). *Column chromatography eluent: DCM on base-treated silica (run through 5% NEt_3 in hexane before adding compound to column).* **9b** (543 mg, 50%) as a yellow oil. ^{1}H NMR (400 MHz, $CDCl_3$): δ 2.58 (3H, s, S*CH_3*); 7.18 (1H, td, *J* = 7.5 Hz, 1.1 Hz, Ar-*H*); 7.22-7.26 (1H, m, Ar-*H*); 7.33-7.39 (1H, m, Ar-*H*); 7.48 (1H, dd, *J* = 8.3 Hz, 7.3 Hz, Ar-*H*); 7.52 - 7.58 (1H, m, Ar-*H*); 7.60-7.67 (2H, m, Ar-*H*); 7.83 (1H, dd, *J* = 7.2 Hz, 1.1 Hz,); 7.84 - 7.91 (2H, m, Ar-*H*); 8.63 (1H, d, *J* = 8.3 Hz, Ar-*H*); ^{13}C{^{1}H} NMR (100.6 MHz, $CDCl_3$): δ 15.3, 91.7, 94.1, 120.9, 121.6, 124.3, 124.4, 125.3, 126.5, 126.6, 126.9, 128.2, 128.87, 128.92, 130.6, 132.5, 133.2, 133.3, 141.7 [GC-MS] *m/z* calculated for $C_{19}H_{14}S$, 274.1; found 274.1. GC-MS retention times of analytes: 14.23 minutes: Methyl(2-(naphthalen-1-ylethynyl)phenyl)sulfane; [Acc. Mass] Calculated $[M+H]^+$: 275.0889 gmol^{-1}, Observed: $[M+H]^+$ 275.0892 gmol^{-1}.

(2-(mesitylethynyl)phenyl)(methyl)sulfane **(9c)**

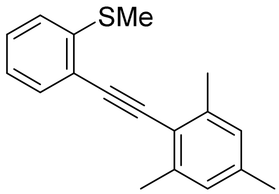

Prepared according to general procedure A. 2-iodothioanisole (1.6 g, 6.30 mmol, 1 eq.), 2-ethynylmesitylene (1 g, 6.93 mmol, 1.1 eq), $Pd(PPh_3)_4$ (146 mg, 0.126 mmol, 0.02 eq.), copper(I) iodide (48 mg, 0.252 mmol, 0.04 eq.), triethylamine (8 mL) and THF (10 mL). *Column chromatography eluent: 15% DCM in 40-60 petroleum ether*. **9c** (1.59 g, 95%) obtained as an orange solid. ^1H NMR (400 MHz, $CDCl_3$): δ 2.34 (3H, s, SCH_3); 2.54 (3H, s, Mes(*p*-CH_3)); 2.58 (6H, s, C≡C-Mes(*o*-CH_3)$_2$); 6.94 (2H, s, Mes-*H*); 7.15 (1H, t, J = 7.6 Hz, Ar-*H*); 7.21 (1H, d, J = 7.8 Hz, Ar-*H*); 7.30-7.40 (1H, m, Ar-*H*); 7.54(1H, d, J = 7.8 Hz, Ar-*H*); $^{13}C\{^1H\}$ NMR (100.6 MHz, $CDCl_3$): δ 15.1, 21.3, 21.5, 94.0, 94.4, 120.0, 121.9, 123.8, 124.2, 127.7, 128.5, 132.3, 138.0, 140.4, 141.1; [GC-MS] *m/z* calculated for $C_{18}H_{18}S$, 266.1; found 266.1. GC-MS retention times of analytes: 12.98 minutes: (2-(mesitylethynyl)phenyl)(methyl)sulfane; [Acc. Mass] Calculated $[M+H]^+$: 267.1202 $gmol^{-1}$, Observed $[M+H]^+$: 267.1198 $gmol^{-1}$.

3-((2-(methylthio)phenyl)ethynyl)thiophene **(9d)**

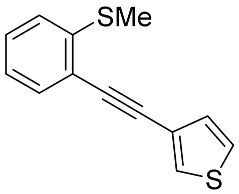

Prepared according to general procedure A. 2-iodothioanisole (1 g, 4.00mmol, 1 eq.), 3-ethynylthiophene (0.47 mL, 4.80 mmol, 1.2 eq), $Pd(PPh_3)_4$ (139 mg, 0.120 mmol, 0.02 eq.), copper(I) iodide (46 mg, 0.240 mmol, 0.04 eq.), triethylamine (8 mL) and THF (10 mL). *Column chromatography eluent: 20% DCM in 40-60 petroleum ether*. **9d** (0.62 g, 68%) obtained as an orange oil. ^{1}H NMR (400 MHz, $CDCl_3$): δ 2.52 (3H, s, SC*H$_3$*); 7.12 (1H, td, J = 7.6 Hz, 1.3 Hz, Ar-*H*); 7.18 (1H, d, J = 8.1 Hz, Ar-*H*); 7.25 (1H, dd, J = 5.0 Hz, 1.3 Hz, Ar-*H*); 7.29-7.34 (2H, m, Ar-*H*); 7.48 (1H, dd, J = 7.6 Hz, 1.3 Hz, Ar-*H*); 7.58 (1H, dd, J = 3.0 Hz, 1.3 Hz, Ar-*H*); $^{13}C\{^1H\}$ NMR (100.6 MHz, $CDCl_3$): δ 15.1, 86.3, 91.0, 121.3, 122.2, 124.1, 124.3, 125.4, 128.7, 128.8, 129.9, 132.2, 141.6; [GC-MS] *m/z* calculated for $C_{13}H_{10}S_2$, 230.0; found 230.0. GC-MS retention times of analytes: 12.08 minutes: 3-((2-(methylthio)phenyl)ethynyl)thiophene; [Acc. Mass] Calculated $[M+H]^+$: 231.0296 gmol^{-1}, Observed: $[M+H]^+$ 231.0306 gmol^{-1}.

4. Cyclisation of 2-alkynylanisoles

General Procedure

Due to the variable molarity of commercial BCl_3 solutions an excess of BCl_3 was formally used. All commercial BCl_3 solutions from various vendors labelled as 1M in DCM were actually found to be lower than 1M by varying amounts (by NMR titration experiments with PPh_3). Therefore between 1.1 – 1.4 equivalents of "1M" BCl_3 are used, which really approximates to a 1:1 ratio between alkyne and BCl_3. To minimize the gradual decrease in molarity of BCl_3 solutions over time due to the high volatility of BCl_3 the BCl_3 solutions are transferred to ampoules sealed with J. Youngs valves after first use. An excess of BCl_3 can be used rather than determining the molarity of BCl_3, with removal of solvent in-vacuo also removing any excess of BCl_3.

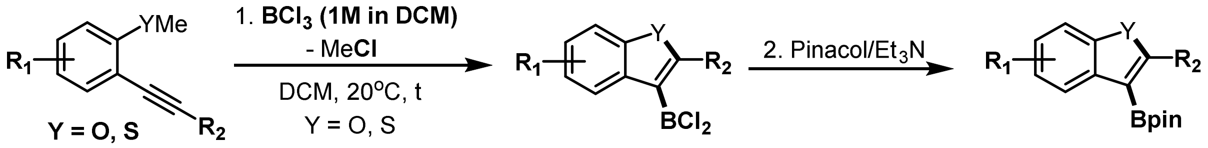

The alkyne (1 eq.) was dissolved in DCM and boron trichloride ("1M" in DCM, 1.1-1.4 eq) was added. The reaction was stirred (or rotated at 10 revolutions per minute if performed in a J. Youngs NMR tube) at room temperature for the specified time until reaction completion and the solvent was removed *in vacuo* (also removing any unreacted BCl_3). The resulting oil was then re-dissolved in DCM, and transferred via cannula to a 0-5°C solution of pinacol (1.1 eq.) and NEt_3 (approx. 15 eq.) in DCM **(Caution: the esterification is highly exothermic!).** All subsequent steps were performed under air with non-purified solvents. The solvent was removed *in vacuo* and the product was extracted into pentane and filtered. The filtrate was collected and the solvent removed *in vacuo*. If necessary the resulting material was purified further via column chromatography to yield the corresponding boronate ester. It should be noted the majority of boronate esters did not require column chromatography, with a simple filtration through a plug of silica sufficient to yield pure material.

4,4,5,5-tetramethyl-2-(2-phenylbenzofuran-3-yl)-1,3,2-dioxaborolane **(4a)**

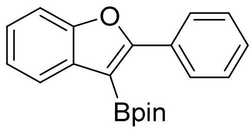

Prepared according to the general procedure. Alkyne **3a** (130 mg, 0.630 mmol, 1 eq.), BCl_3 (0.75 mL, 0.750 mmol, 1.2 eq.), DCM (0.5 mL), NEt_3 (1.5 mL) and pinacol (78 mg, 0.660 mmol, 1.05 eq.). Filtered through silica with pentane for purification. **4a** (112 mg, 56%) obtained as an orange film. ^{1}H NMR (400 MHz, $CDCl_3$): δ 1.40 (12H, s, B*pin*); 7.21-7.33 (2H, m, Ar-*H*); 7.37-7.47 (3H, m, Ar-*H*); 7.49-7.53 (1H, m, Ar-*H*); 8.00-8.03 (1H, m, Ar-*H*); 8.13-8.19 (2H, m, Ar-*H*); $^{13}C\{^{1}H\}$ NMR (100.6 MHz, $CDCl_3$): δ 23.9, 82.6, 109.5, 121.9, 122.1, 123.2, 127.1, 128.1, 130.3, 132.3, 153.6, 161.9; ^{11}B NMR (128.6MHz, $CDCl_3$); δ 30.6 (s); [GC-MS] *m/z* calculated for $C_{20}H_{21}BO_3$, 320.2; found 320.2. GC-MS retention times of analytes: 16.31 minutes: 4,4,5,5-tetramethyl-2-(2-phenylbenzofuran-3-yl)-1,3,2-dioxaborolane; [Acc. Mass] Calculated $[M+H]^+$: 321.1657 $gmol^{-1}$, Observed $[M+H]^+$: 321.1655 $gmol^{-1}$.

4,4,5,5-tetramethyl-2-(5-methyl-2-phenylbenzofuran-3-yl)-1,3,2-dioxaborolane **(4b)**

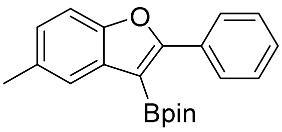

Prepared according to the general procedure. Alkyne **3b** (44 mg, 0.198 mmol, 1 eq.), BCl_3 (0.24 mL, 0.240 mmol, 1.2 eq.), DCM (0.5 mL), NEt_3 (0.5 mL) and pinacol (28 mg, 0.240 mmol, 1.2 eq.). Filtered through silica with pentane for purification. **4b** (59 mg, 89%) obtained as a colourless film. ^{1}H NMR (400 MHz, $CDCl_3$): δ 1.43 (12H, s, B*pin*); 2.50 (3H, s, Ar-CH_3); 7.11 (1H, dd, J = 8.3 Hz, 1.8 Hz, Ar-*H*); 7.38-7.49 (4H, m, Ar-*H*); 7.76-7.79 (1H, m, Ar-*H*); 8.14-8.18 (2H, m, Ar-*H*); $^{13}C\{^{1}H\}$ NMR (100.6 MHz, $CDCl_3$): δ 21.6, 25.0, 83.6, 110.1, 122.8, 125.5, 128.1, 129.0, 131.4, 132.4, 133.3, 153.1, 163.1; ^{11}B NMR (128.6MHz, $CDCl_3$); δ 30.5 (s); [GC-MS] *m/z* calculated for $C_{21}H_{23}BO_3$**:** 334.2; found 334.2. GC-MS retention times of analytes: 13.44 minutes: 4,4,5,5-tetramethyl-2-(5-methyl-2-phenylbenzofuran-3-yl)-1,3,2-dioxaborolane; [Acc. Mass] Calculated $[M]^+$: 334.1740 $gmol^{-1}$, Observed $[M]^+$: 334.1733 $gmol^{-1}$.

2-(5-chloro-2-phenylbenzofuran-3-yl)-4,4,5,5-tetramethyl-1,3,2-dioxaborolane **(4c)**

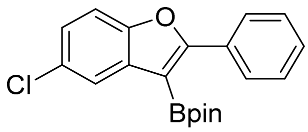

Prepared according to the general procedure. Alkyne **3c** (44 mg, 0.181 mmol, 1 eq.), BCl_3 (0.22 mL, 0.220 mmol, 1.2 eq.), DCM (0.5 mL), NEt_3 (0.5 mL) and pinacol (26 mg, 0.220 mmol, 1.2 eq.). Filtered through silica with pentane for purification. **4c** (46 mg, 72%) obtained as a colourless film. ^{1}H NMR (400 MHz, $CDCl_3$): δ 1.42 (12H, s, B*pin*); 7.25 (1H, dd, J = 8.6 Hz, 2.3 Hz, Ar-*H*); 7.40-7.49 (4H, m, Ar-*H*); 7.98 (1H, dd, J = 2.3 Hz, 0.5 Hz, Ar-*H*); 8.14-8.18 (2H, m, Ar-*H*); $^{13}C\{^{1}H\}$ NMR (100.6 MHz, $CDCl_3$): δ 24.9, 83.8, 111.5, 122.7, 124.4, 128.2, 128.3, 128.5, 129.5, 130.8, 134.8, 153.0, 164.4; ^{11}B NMR (128.6MHz, $CDCl_3$); δ 30.4 (s); [GC-MS] *m/z* calculated for $C_{20}H_{20}BClO_3$**:** 354.2; found 354.2. GC-MS retention times of analytes: 13.80 minutes: 2-(5-chloro-2-phenylbenzofuran-3-yl)-4,4,5,5-tetramethyl-1,3,2-dioxaborolane ; [Acc. Mass] $[M]^+$ 354.1189 $gmol^{-1}$ [Acc. Mass] Calculated $[M]^+$: 354.1194 $gmol^{-1}$, Observed $[M]^+$: 354.1189 $gmol^{-1}$.

2-(7-fluoro-2-phenylbenzofuran-3-yl)-4,4,5,5-tetramethyl-1,3,2-dioxaborolane **(4d)**

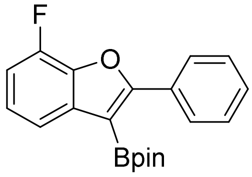

Prepared according to the general procedure. Alkyne **3d** (91 mg, 0.402 mmol, 1 eq.), BCl_3 (0.53 mL, 0.52 mmol, 1.3 eq.), DCM (0.5 mL), NEt_3 (1 mL) and pinacol (65 mg, 0.550 mmol, 1.4 eq.). Filtered through silica with pentane for purification. **4d** (107 mg, 79%) obtained as a colourless film. ^{1}H NMR (400 MHz, $CDCl_3$): δ 1.45 (12H, s, B*pin*); 7.06 (1H, ddd, J = 10.6 Hz, 8.1 Hz, 1.0 Hz, Ar-*H*); 7.21 (1H, td, J = 8.1 Hz, 4.5 Hz, Ar-*H*); 7.43-7.53 (3H, m, Ar-*H*); 7.82 (1H, dd, J = 7.8 Hz, 1.0 Hz, Ar-*H*); 8.21-8.25 (2H, m, Ar-*H*); $^{13}C\{^{1}H\}$ NMR (100.6 MHz, $CDCl_3$): δ 24.9 (($\mathit{C}H_3CO)_2B$), 83.8 ($(CH_3\mathit{C}O)_2B$), 110.5 (d, J = 15.5 Hz); 118.7 (d, 3.7 Hz); 123.4 (d, J = 5.9 Hz); 128.3 (d, J = 8.9 Hz); 128.9 (d, J = 13.2 Hz); 129.5, 130.7, 136.9 (d, J = 3.0 Hz); 141.6 (d, J = 11.1 Hz); 142.6 (d, J = 248.4 Hz); 163.8; ^{11}B NMR (128.6MHz, $CDCl_3$); δ 30.5 (s); ^{19}F NMR (376.50 MHz, $CDCl_3$): δ -137.66; [GC-MS] *m/z* calculated for $C_{20}H_{20}BFO_3$**:** 338.2; found 338.2.

GC-MS retention times of analytes: 16.19 minutes: 2-(7-fluoro-2-phenylbenzofuran-3-yl)-4,4,5,5-tetramethyl-1,3,2-dioxaborolane; [Acc. Mass] Calculated $[M]^+$: 338.1490 $gmol^{-1}$, Observed $[M]^+$: 338.1486 $gmol^{-1}$.

<u>4,4,5,5-tetramethyl-2-(4-methyl-2-phenylbenzofuran-3-yl)-1,3,2-dioxaborolane **(4e)**</u>

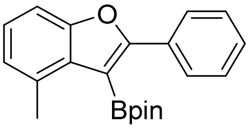

In a J. Young's NMR tube, Alkyne **3e** (32 mg, 0.144 mmol, 1 eq.) and 2,4,6-tri-*tert*-butylpyridine (TBP) (36 mg, 0.144 mmol, 1 eq.) were dissolved in DCM (0.5 mL). BCl_3 (0.53 mL, 0.52 mmol, 1.3 eq.) was added and the mixture was left for 12 hours at 20°C. After this time, the mixture was layered with NEt_3 (1 mL) and pinacol (18 mg, 0.152 mmol, 1.25 eq.) was added. The tube was inverted to mix, and the crude compound was immediately generated. This was purified via preparative TLC (Eluent: 15% DCM in 40-60 petroleum ether) to give **4e** (16 mg, 33%) as a white solid. ^1H NMR (400 MHz, $CDCl_3$): δ 1.41 (12H, s, B*pin*); 2.66 (3H, m, Ar-C*H₃*); 7.02 (1H, dt, J = 7.3 Hz, 0.7 Hz, Ar-*H*); 7.18 (1H, t, J = 7.8 Hz, Ar-*H*); 7.34-7.47 (4H, m, Ar-*H*); 7.91-7.95 (2H, m, Ar-*H*); $^{13}C\{^1H\}$ NMR (100.6 MHz, $CDCl_3$): δ 20.7, 25.1, 84.3, 108.4, 123.9, 124.2, 127.8, 128.2, 128.8, 131. 4, 131.7, 132.4, 155.1,; ^{11}B NMR (128.6MHz, $CDCl_3$); δ 31.7 (s); [GC-MS] *m/z* calculated for $C_{21}H_{23}BO_3$**:** 334.2; found 334.2. GC-MS retention times of analytes: 13.49 minutes: 4,4,5,5-tetramethyl-2-(4-methyl-2-phenylbenzofuran-3-yl)-1,3,2-dioxaborolane; [Acc. Mass] Calculated $[M+H]^+$: 335.1813 $gmol^{-1}$, Observed $[M+H]^+$: 335.1811 $gmol^{-1}$

TBP was added to this reaction mixture as trace hydrolysis of BCl_3 generates HCl which can lead to alkyl migration as previously observed.[11] Indeed on addition of TBP a small quantity of protonated TBP was observed.

2-(2-benzylbenzofuran-3-yl)-4,4,5,5-tetramethyl-1,3,2-dioxaborolane **(4f)**

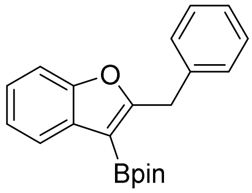

Prepared according to the general procedure. Alkyne **3f** (30 mg, 0.135 mmol, 1 eq.), BCl_3 (0.16 mL, 0.160 mmol, 1.2 eq.), DCM (0.5 mL), NEt_3 (0.5 mL) and pinacol (19 mg, 0.160 mmol, 1.2 eq.). Filtered through silica with pentane for purification. **4f** (26 mg, 57%) obtained as a colourless film. ^{1}H NMR (400 MHz, $CDCl_3$): δ 1.42 (12H, s, B*pin*); 4.40 (2H, s, PhC*H₂*); 7.20-7.27 (3H, m, Ar-*H*); 7.32 (2H, t, J = 7.6 Hz); 7.38-7.43 (3H, m, Ar-*H*); 7.89-7.93 (1H, m, Ar-*H*); $^{13}C\{^{1}H\}$ NMR (100.6 MHz, $CDCl_3$): δ 25.0, 34.8, 83.3, 110.6, 122.5, 122.7, 123.5, 126.5, 128.5, 128.9, 131.8, 138.2, 155.1, 167.1; ^{11}B NMR (128.6MHz, $CDCl_3$); δ 30.4 (s); [GC-MS] *m/z* calculated for $C_{21}H_{23}BO_3$**:** 334.2; found 334.2. GC-MS retention times of analytes: 13.00 minutes: 2-(2-benzylbenzofuran-3-yl)-4,4,5,5-tetramethyl-1,3,2-dioxaborolane; [Acc. Mass] Calculated $[M+H]^+$: 335.1813 $gmol^{-1}$, Observed $[M+H]^+$: 335.1824 $gmol^{-1}$

4,4,5,5-tetramethyl-2-(2-methylbenzofuran-3-yl)-1,3,2-dioxaborolane **(4g)**

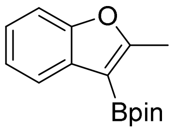

Small scale: Prepared according to the general procedure using a J. Young's NMR tube. Alkyne **3g** (31 mg, 0.212 mmol, 1 eq.), BCl_3 (0.23 mL, 0.230 mmol, 1.1 eq.), DCM (0.5 mL), NEt_3 (0.5 mL) and pinacol (27 mg, 0.230 mmol, 1.1 eq.). Filtered through silica with pentane for purification. **4g** (48 mg, 89%) obtained as a white solid.

Larger scale: Prepared according to the general procedure. Alkyne **3g** (867 mg, 5.931 mmol, 1 eq.), BCl_3 (7.7 mL, 7.71 mmol, 1.3 eq.), DCM (5 mL), NEt_3 (5 mL) and pinacol (940 mg, 7.95 mmol, 1.3 eq.). Filtered through silica with pentane for purification. **4g** (1.16 g, 76%) obtained as a white solid.

Non-purified solvents under ambient conditions: In a 100 mL round-bottomed flask open to the atmosphere, alkyne **3g** (51mg, 0.345mmol, 1 eq.), BCl_3 (0.41 mL, 0.410 mmol, 1.1 eq.), non-purified DCM (0.5 mL) were added. The mixture was stirred for five minutes before non-purified NEt_3 (1 mL) and pinacol (49 mg, 0.41 mmol, 1.1 eq.) were added. The crude mixture was extracted and filtered through silica using pentane to give **4g** (72 mg, 81%) as a white solid.

^{1}H NMR (400 MHz, $CDCl_3$): δ 1.38 (12H, s, B*pin*); 2.65 (3H, s, Ar-C*H₃*); 7.18-7.25 (2H, m, Ar-*H*); 7.38-7.44 (1H, m, Ar-*H*); 7.82-7.88 (1H, m, Ar-*H*); ^{13}C{^{1}H} NMR (100.6 MHz, $CDCl_3$): δ 14.5, 25.0, 83.1, 110.2, 122.1, 122.7, 123.2, 132.1, 154.8, 165.8; ^{11}B NMR (128.6MHz, $CDCl_3$); δ 30.3 (s); [GC-MS] *m/z* calculated for $C_{15}H_{19}BO_3$, 258.1; found 258.1. GC-MS retention times of analytes: 10.73 minutes: 2-(2,5-dimethylbenzofuran-3-yl)-4,4,5,5-tetramethyl-1,3,2-dioxaborolane; [Acc. Mass] Calculated $[M+H]^+$: 259.1500 gmol^{-1}, Observed $[M+H]^+$: 259.1499 gmol^{-1}

Ethyl 4-(3-(4,4,5,5-tetramethyl-1,3,2-dioxaborolan-2-yl)benzofuran-2-yl)benzoate **(4h)**

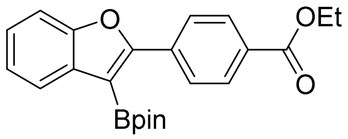

In a J. Young's NMR tube, alkyne **3h** (20 mg, 0.071 mmol, 1 eq.) and TBP (20 mg, 0.071 mmol, 1 eq.) were dissolved in DCM (0.5 mL). BCl_3 (0.24 mL, 0.240 mmol, 3.3 eq.) was added and the mixture was left for 12 hours at 20°C. After this time, the mixture was layered with NEt_3 (1 mL) and pinacol (29 mg, 0.245 mmol, 3.5 eq.) was added. The tube was inverted to mix, and the crude compound was immediately generated. This was purified via preparative TLC (Eluent: 20% DCM in 40-60 petroleum ether) to give **4h** (13 mg, 46%) as a white solid. ^{1}H NMR (400 MHz, $CDCl_3$): δ 1.43 (12H, s, B*pin*); 1.44 (3H, t, J = 7.1 Hz, $COOCH_2CH_3$); 4.42 (2H, q, J = 7.1 Hz, $COOCH_2CH_3$); 7.25-7.36 (2H, m Ar-*H*); 7.52-7.55 (1H, m, Ar-*H*); 8.04 (1H, m, Ar-*H*); 8.12 (2H, dt, J = 8.4 Hz, 1.7 Hz, Ar-*H*); 8.28 (2H, dt, J = 8.4 Hz, 1.7 Hz, Ar-*H*); ^{13}C{^{1}H} NMR (100.6 MHz, $CDCl_3$): δ 14.4, 25.0, 61.1, 83.9, 110.7, 123.2, 123.4, 124.9, 127.8, 129.4, 130.4, 133.1, 135.4, 154.8, 161.4, 166.4; ^{11}B NMR (128.6MHz, $CDCl_3$); δ 30.4 (s); [GC-MS] *m/z* calculated for $C_{23}H_{25}BO_5$**:** 392.2; found 392.2. GC-MS retention times of analytes: 15.33 minutes: Ethyl 4-(3-(4,4,5,5-tetramethyl-1,3,2-dioxaborolan-2-yl)benzofuran-2-yl)benzoate; [Acc. Mass] Calculated $[M+H]^+$: 393.1868 gmol^{-1}, Observed $[M+H]^+$: 393.1868 gmol^{-1}

Excess BCl_3 was utilised in this case as one equivalent of BCl_3 coordinates to the ester moiety.

5. O-Directed Transhaloboration

Oxygen Directed (**5**)

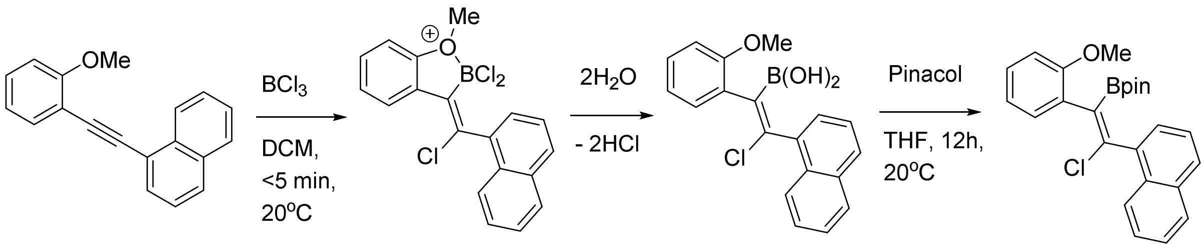

In a Young's NMR tube, alkyne **3j** (35 mg, 0.135 mmol, 1 eq.) was dissolved in DCM (0.4 mL). BCl_3 (0.15 mL, 0.150 mmol, 1.1 eq.) was added. After five minutes, an excess of water was added and the tube was sonicated to assist with mixing. The resulting boronic acid was then stirred with pinacol (18 mg, 0.149 mmol, 1.1 eq.) in THF (5 mL) to generate the esterified product. This was purified via chromatography (20% DCM in 40-60 petroleum ether), however two very minor impurities, the starting alkyne and the borylated benzofuran (from the cyclisation pathway), were observed that could not be completely separated in our hands. Data reported for the major component, which corresponds to the haloboration product.

^{1}H NMR (400 MHz, $CDCl_3$): δ 1.39 (6H, s, B*pin*); 1.41 (6H, s, B*pin*); 3.74 (3H, s, O*Me*); 6.37 (1H, td, J = 7.6 Hz, 1.0 Hz, Ar-*H*); 6.62 (1H, dd, J = 7.6 Hz, 1.1 Hz, Ar-*H*); 6.67 (1H, d, J = 8.1 Hz, Ar-*H*); 6.95 (1H, td, J = 8.3 Hz, 1.7 Hz, Ar-*H*); 7.23-7.31 (2H, m, Ar-*H*); 7.42-7.51 (2H, m, Ar-*H*); 7.70 (1H, d, J = 7.6 Hz, Ar-*H*); 7.78 (1H, d, J = 7.3 Hz, Ar-*H*); 8.20 (1H, d, J = 8.3 Hz, Ar-*H*); ^{11}B NMR (128.6MHz, $CDCl_3$); δ 31.0 (s); ^{13}C{^{1}H} NMR (100.6 MHz, $CDCl_3$): δ24.8, 24.9, 55.1, 84.0, 109.8, 120.3, 125.1, 125.8, 126.0, 126.2, 127.3, 128.1, 128.2, 128.3, 128.8, 130.0, 130.7, 133.4, 137.2, 138.3, 156.1 [APCI] *m/z* calculated for $[M+H]^+$ 421.2; found 421.1; $[M-Cl]^+$ Calculated 385.2; found 385.2; $[M+NH_4]^+$ Calculated 438.2; Found 438.2 [Acc. Mass] $[M+H]^+$ Calculated 421.1736; Found 421.1743

6. External Screening

General Procedure

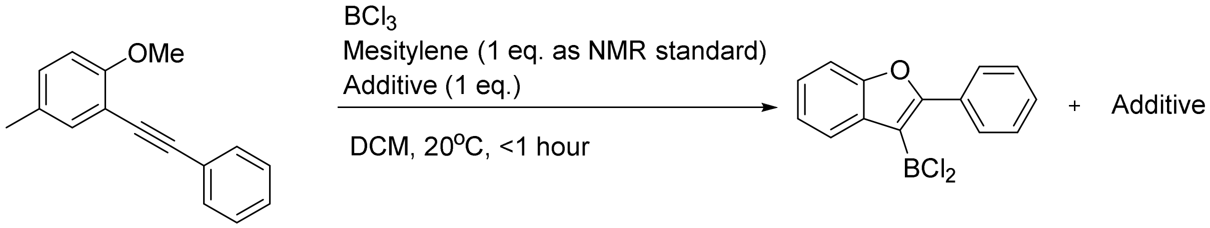

In a J. Young's NMR tube, alkyne **3b** (30mg) was dissolved in DCM (0.5 mL) with a known amount of mesitylene to act as an internal standard, and one equivalent of an additive. BCl_3 was added and the *in-situ* yields were determined within one hour using NMR spectroscopy to compare the quantities of desired product and additive against the mesitylene standard.

Additive	BCl_3 Equivalents	Substrate Consumption (%)	NMR Conversion To Product (%)	Timescale (h)	
No additive	**1.2**	**100**	**96**	**<0.5**	
Nitromethane	**1.2**	**100**	**84**	**<0.5**	
Acetone	**2.2**	**100**	**38**	**<0.5**	
Trifluorotoluene	**1.2**	**100**	**90**	**<0.5**	
N,N-Dimethylaniline	**2.2**	**100**	**74**	**<0.5**	
Benzaldehyde	**2.2**	**100**	**17**	**<0.5**	
Hex-1-ene	**1.2**	**100**	**91**	**<0.5**	
N,N-Dimethylbenzamide	**2.2**	**100**	**72**	**<0.5**	
2-bromopyridine	**2.2**	**100**	**86**	**<0.5**	
4-bromobenzonitrile	**1.2**	**51**	**45**	**<0.5**	
	**1.2**	**100**	***72**	**24**	**Aryl BCl_2 with coordinated CN**
	**2.2**	**100**	**96**	**<0.5**	

Table 1. Additives used in the external functionality screening

7. Double Borylative Cyclisation

2,2'-(2,6-diphenylbenzo[1,2-b4,5-b']difuran-3,7-diyl)bis(4,4,5,5-tetramethyl-1,3,2-dioxaborolane) **(7)**

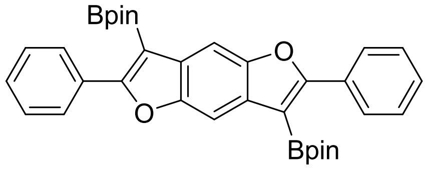

Prepared according to the general procedure. Alkyne **6** (118 mg, 0.358 mmol, 1 eq.), BCl_3 (0.79 mL, 0.790 mmol, 2.2 eq.), DCM (4 mL), NEt_3 (4 mL) and pinacol (91mg, 0.790 mmol, 2.2 eq.). Filtered through silica with pentane for purification. **7** (176 mg, 88%) obtained as a white solid. ^{1}H NMR (400 MHz, $CDCl_3$): δ 1.45 (24H, s, B*pin*); 7.37-7.50 (6H, m, Ar-*H*); 8.10 (2H, s, 2(C-*H*) on central ring); 8.20-8.24 (4H, m, Ar-*H*); ^{13}C{^{1}H} NMR (100.6 MHz, $CDCl_3$): δ 25.0, 83.7, 103.3, 128.12, 128.16, 129.0, 131.4, 131.5, 152.1, 163.5; ^{11}B NMR (128.6MHz, $CDCl_3$); δ 31.0 (s); [APCI] *m/z* calculated for $C_{34}H_{36}B_2O_6$, 563.3; found 563.3; [Acc. Mass] Calculated $[M+H]^+$: 563.2771 gmol^{-1}, Observed $[M+H]^+$: 563.2755 gmol^{-1}

8. In-situ Cross-coupling

5-methyl-2-phenyl-3-(p-tolyl)benzofuran **(8)**

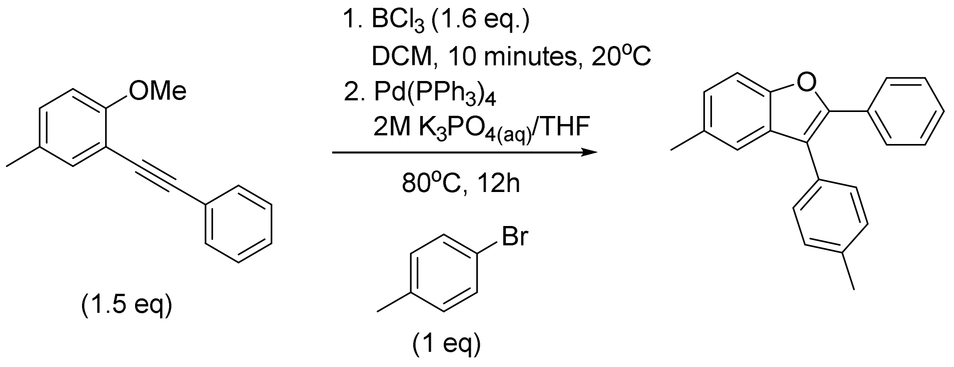

In a Schlenk fitted with a J. Young valve, BCl_3 (0.54 mL, 0.540 mmol, 1.7 eq.) was added to a solution of alkyne **3b** (100 mg, 0.450 mmol, 1.5 eq.) in DCM (2 mL) and stirred for 10 minutes. After this time, the solvent and any remaining BCl_3 were removed *in vacuo* and $Pd(PPh_3)_4$ (21 mg, 0.018 mmol, 0.06 mmol) and 4-bromotoluene (51 mg, 0.298 mmol, 1 eq.) were added as solids. A solution of aqueous 2M K_3PO_4 (1.1 mL, 2.250 mmol, 7.5 eq.) in THF (10 mL) was degassed for 30 minutes and added to the ampoule. The resulting solution was stirred and refluxed for 12 hours. After this time, the solution was allowed to cool to 20°C and Et_2O (15 mL) was added. The crude mixture was washed with distilled water (3 x 10 mL) and the organic layer was dried over $MgSO_4$ and evaporated *in vacuo*. The resulting oil was purified via column chromatography (10% DCM / 90% 40-60 petroleum ether) to give benzofuran **8** (63 mg, 72% based on 4-bromo-toluene) as a colourless oil. ^{1}H NMR (400 MHz, $CDCl_3$): δ 2.44 (3H, s, Ar-*Me*); 2.47 (3H, s, Ar-*Me*); 7.15 (1H, d, J = 8.3 Hz, Ar-*H*); 7.27-7.36 (6H, m, Ar-*H*); 7.38-7.47 (3H, m, Ar-*H*); 7.68 (2H, d, J = 7.3 Hz, Ar-*H*); ^{13}C{^{1}H} NMR (100.6 MHz, $CDCl_3$): δ 21.4, 110.6, 117.3, 119.8, 125.9, 126.9, 128.2, 128.4, 129.65, 129.71, 130.0, 130.5, 131.0, 132.4, 137.3, 150.5, 152.4; [GC-MS] *m/z* calculated for $C_{22}H_{18}O$, 298.1; found 298.1. GC-MS retention times of analytes: 17.46 minutes: 5-methyl-2-phenyl-3-(p-tolyl)benzofuran; [Acc. Mass] Calculated $[M+H]^+$ 299.1431 gmol^{-1}, Observed $[M+H]^+$ 299.1430 gmol^{-1}

Isolated cross coupling of 4g with 4-bromo-toluene

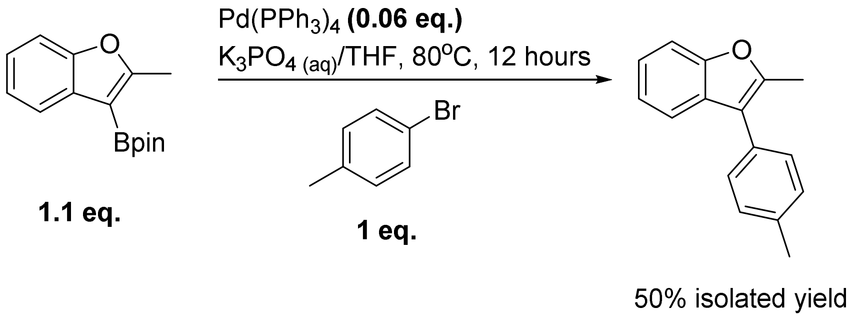

In a Schlenk fitted with a J. Young's valve 4,4,5,5-tetramethyl-2-(2-methylbenzofuran-3-yl)-1,3,2-dioxaborolane **4g** (100 mg, 0.387 mmol, 1.1 eq.), 4-bromotoluene (60 mg, 0.352 mmol, 1 eq.) and $Pd(PPh_3)_4$ (25 mg, 0.021 mmol, 0.06 eq) were added to a degassed solution of 1:1 THF/2M $K_3PO_{4(aq)}$ (10 mL) which was sealed and stirred at 80°C for 12 hours. The mixture was then cooled to 20°C, washed with water (3 x 10 mL) and extracted into ether (3 x 10 mL). The solvent was removed *in vacuo* and the compound was purified via column chromatography (10% DCM in 40-60 petroleum ether) to give the cross coupled product (43 mg, 50%) as a white solid. The data was in accordance with the literature.[12]

In this case, the moderate yield was attributed to protodeboronation of the boronate ester. Typically heteroaryl boronate esters are used in a larger excess to allow complete consumption of the aryl halide due to competitive protodeboronation (for example 1.5 eq of the alkyne (and thus boronate ester) was used in the one-pot borylative cyclisation cross-coupling).

9. Cyclisation of 2-alkynylthioanisoles

4,4,5,5-tetramethyl-2-(2-phenylbenzo[b]thiophen-3-yl)-1,3,2-dioxaborolane **(11a)**

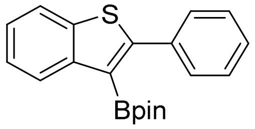

Prepared according to the general procedure. Alkyne **9a** (100 mg, 0.446 mmol, 1 eq.), BCl_3 (0.56mL, 0.560 mmol, 1.2 eq.), DCM (1 mL), NEt_3 (1 mL) and pinacol (70 mg, 0.592 mmol, 1.3 eq.). Filtered through silica with pentane for purification. **11a** (57 mg, 38%) obtained as a white solid. ^{1}H NMR (400 MHz, $CDCl_3$): δ 1.35 (12H, s, B*pin*); 7.30 - 7.37 (1H, m, Ar-*H*); 7.37 - 7.47 (4H, m, Ar-*H*); 7.60 - 7.68 (2H, m, Ar-*H*); 7.82 - 7.89 (1H, m, Ar-*H*); 8.22 - 8.29 (1H, m, Ar-*H*); ^{13}C{^{1}H} NMR (100.6 MHz, $CDCl_3$): δ 24.8, 83.6, 121.6, 124.0, 124.4, 125.2, 127.9, 128.4, 129.8, 135.4, 140.5, 144.8, 154.9; ^{11}B NMR (128.6MHz, $CDCl_3$); δ 30.32 (s); [GC-MS] *m/z* calculated for $C_{20}H_{21}BO_2S$, 336.2; found 336.2. GC-MS retention times of analytes: 13.56 minutes: 4,4,5,5-tetramethyl-2-(2-phenylbenzo[b]thiophen-3-yl)-1,3,2-dioxaborolane; [Acc. Mass] Calculated $[M+H]^+$: 337.1428 gmol^{-1}, Observed $[M+H]^+$: 337.1425 gmol^{-1}

Modified procedure – with addition of NEt_3 / $AlCl_3$

Further investigations into the reaction involved the direct comparison of the *in-situ* NMR spectra between the sulphur and oxygen analogues. It was concluded that the thioanisole moiety was not demethylated *in-situ*, leading to the generation of a zwitterionic sulphonium intermediate **10**:

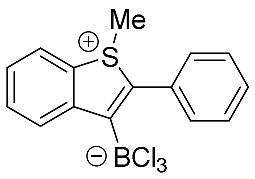

Compound 10

Adapted procedure – NEt_3 followed by $AlCl_3$

In a J. Young's NMR tube, alkyne **9a** (56 mg, 0.249 mmol, 1 eq.), DCM (0.4 mL), BCl_3 (0.55mL, 0.55 mmol, 2.2 eq.) were added. An equivalent of $AlCl_3$ (33 mg, 1 eq. 0.249 mmol) was added to abstract the chloride, followed by demethylation with an excess of NEt_3 (0.5 mL) to generate the neutral thiophene-BCl_2 compound (presumably as the Et_3N adduct) and

[$MeNEt_3$][$AlCl_4$]. 3 equivalents of pinacol (88 mg, 0.747 mmol) were added with an excess used to ensure complete esterification of the borylated species (as pinacol also reacts with the $AlCl_4$). This was purified via chromatography (20% DCM in 40-60 petroleum ether) to give the desired compound **11a** (57 mg, 68%).

4,4,5,5-tetramethyl-2-(2-(naphthalen-1-yl)benzo[b]thiophen-3-yl)-1,3,2-dioxaborolane **(11b)**

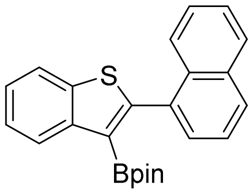

Prepared according to the general procedure using a J. Young's NMR tube. Alkyne **9b** (10 mg, 0.036 mmol, 1 eq.), BCl_3 (0.44 mL, 0.440 mmol, 1.2 eq.), DCM (0.5 mL), NEt_3 (0.5 mL) and pinacol (5 mg, 0.042 mmol, 1.2 eq.). Purified via preparative TLC (Eluent: 30% DCM in 40-60 petroleum ether). **11b** (7 mg, 48%) obtained as a white solid. ^{1}H NMR (400 MHz, $CDCl_3$): δ 0.98 (12H, s, B*pin*); 7.34 - 7.55 (5H, m, Ar-*H*); 7.61 (1H, dd, *J* = 6.9 Hz, 1.1 Hz, Ar-*H*); 7.87-7.93 (4H, m, Ar-*H*); 8.32 (1H, d, J = 7.9 Hz, Ar-*H*); ^{13}C{^{1}H} NMR (100.6 MHz, $CDCl_3$): δ 24.5, 83.1, 121.6, 124.1, 124.5, 124.7, 125.2, 125.6, 126.16, 126.21, 127.9, 128.2, 128.6, 133.1, 133.3, 133.6, 140.9, 144.0, 152.8; ^{11}B NMR (128.6MHz, $CDCl_3$); δ 30.2 (s); [GC-MS] *m/z* calculated for $C_{24}H_{23}BO_2S$, 386.2; found 386.2. GC-MS retention times of analytes: 15.22 minutes: 4,4,5,5-tetramethyl-2-(2-(naphthalen-1-yl)benzo[b]thiophen-3-yl)-1,3,2-dioxaborolane; [Acc. Mass] Calculated $[M+H]^+$: 387.1585 $gmol^{-1}$, Observed $[M+H]^+$: 387.1585 $gmol^{-1}$

Following the previously described adapted procedure ($AlCl_3$ followed by NEt_3), Alkyne **9b** (29 mg, 0.106 mmol, 1 eq.), DCM (0.4 mL), BCl_3 (0.23 mL, 0.23 mmol, 2.2 eq.), $AlCl_3$ (14 mg, 0.106 mmol, 1 eq.), NEt_3 (0.5 mL), pinacol (38 mg, 0.318 mmol, 3eq.). Purified via chromatography (20% DCM in 40-60 petroleum ether) to give a higher yield of **11b** (30 mg, 73%).

2-(2-mesitylbenzo[b]thiophen-3-yl)-4,4,5,5-tetramethyl-1,3,2-dioxaborolane **(11c)**

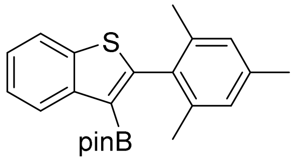

Prepared according to the general procedure. Alkyne **9c** (26 mg, 0.098 mmol, 1 eq.), BCl_3 (0.12mL, 0.120 mmol, 1.2 eq.), DCM (0.5 mL), NEt_3 (0.5 mL) and pinacol (14 mg, 0.120 mmol, 1.2 eq.). Filtered through silica with pentane for purification. **11c** (30 mg, 82%) obtained as a white solid. ^{1}H NMR (400 MHz, $CDCl_3$): δ 1.17 (12H, s, B*pin*); 2.11 (6H, s, Ar-Ph(*o*-C*H*$_3$)$_2$); 2.36 (3H, s, Ar-Ph(*p*-C*H*$_3$)); 6.92 (2H, s, Mes-*H*); 7.31-7.37 (1H, m, Ar-*H*); 7.39-7.44 (1H, m, Ar-*H*); 7.86 (1H, dt, J = 8.1 Hz, 0.8 Hz, Ar-*H*); 8.30 (1H, dq, J = 7.3 Hz, 0.8 Hz); ^{13}C{^{1}H} NMR (100.6 MHz, $CDCl_3$): δ 20.4, 21.2, 24.6, 82.9, 121.7, 123.7, 124.1, 125.1, 127.4, 132.1, 137.4, 141.1, 144.2, 154.7; ^{11}B NMR (128.6MHz, $CDCl_3$); δ 29.2 (s); [GC-MS] *m/z* calculated for $C_{23}H_{27}BO_2S$, 378.3; found 378.3. GC-MS retention times of analytes: 16.8 minutes: 2-(2-mesitylbenzo[b]thiophen-3-yl)-4,4,5,5-tetramethyl-1,3,2-dioxaborolane; [Acc. Mass] Calculated $[M]^+$: 378.1825 gmol^{-1}, Observed $[M]^+$: 378.1824 gmol^{-1}

4,4,5,5-tetramethyl-2-(2-(thiophen-3-yl)benzo[b]thiophen-3-yl)-1,3,2-dioxaborolane **(11d)**

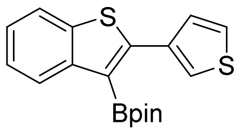

Prepared according to the general procedure. Alkyne **9d** (62 mg, 0.269 mmol, 1 eq.), BCl_3 (0.33 mL, 0.330 mmol, 1.2 eq.), DCM (1 mL), NEt_3 (1 mL) and pinacol (34 mg, 0.287 mmol, 1.2 eq.). Filtered through silica with pentane for purification. **11d** (49 mg, 55%) obtained as a white solid. ^{1}H NMR (400 MHz, $CDCl_3$): δ 1.39 (12H, s, B*pin*); 7.28-7.42 (3H, m, Ar-*H*); 7.44 (1H, dd, J = 5.0 Hz, 1.3 Hz, Ar-*H*); 7.69 (1H, dd, J = 3.0 Hz, 1.3 Hz, Ar-*H*); 7.81 (1H, d, J = 7.8 Hz, Ar-*H*); 8.25 (1H, d, J = 8.3 Hz, Ar-*H*); ^{13}C{^{1}H} NMR (100.6 MHz, $CDCl_3$): δ 24.9, 83.7, 121.5, 124.1, 124.5, 124.7, 124.9, 125.2, 129.3, 135.9, 139.8, 144.9, 148.9; ^{11}B NMR (128.6MHz, $CDCl_3$); δ 30.0 (s); [GC-MS] *m/z* calculated for $C_{18}H_{19}BO_2S_2$, 342.1; found 342.1. GC-MS retention times of analytes: 13.75 minutes: 4,4,5,5-tetramethyl-2-(2-(thiophen-3-

yl)benzo[b]thiophen-3-yl)-1,3,2-dioxaborolane; [Acc. Mass] Calculated $[M+H]^+$: 343.0992 $gmol^{-1}$, Observed $[M+H]^+$: 343.0991 $gmol^{-1}$

Attempts using the adapted procedure involving addition of $AlCl_3$ / Et_3N prior to pinacol led to complex mixtures containing intractable material, this is attributed to AlCl3 reacting with the alpha thienyl positions.

10. In-situ NMR Spectra

Anisole + BCl_3 (*in situ*) – 20 minutes vs 30 hours

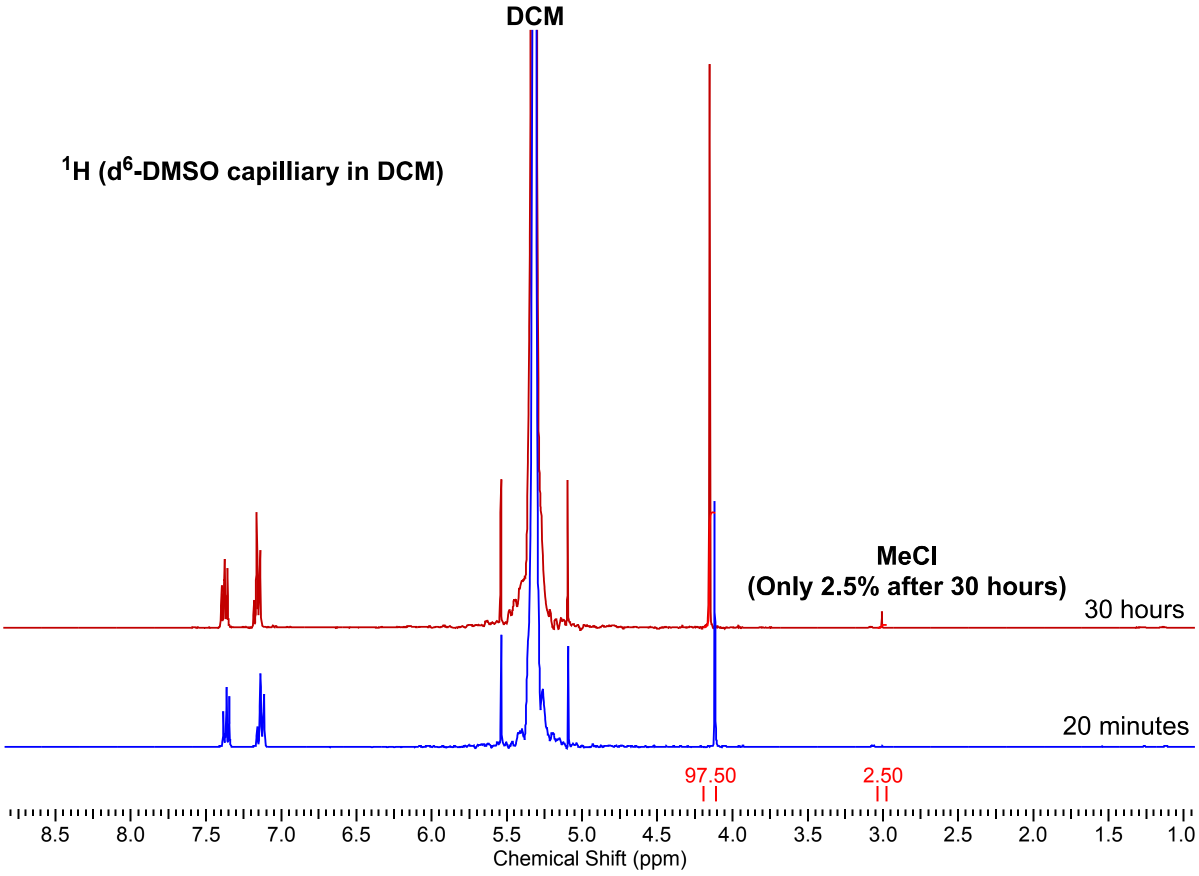

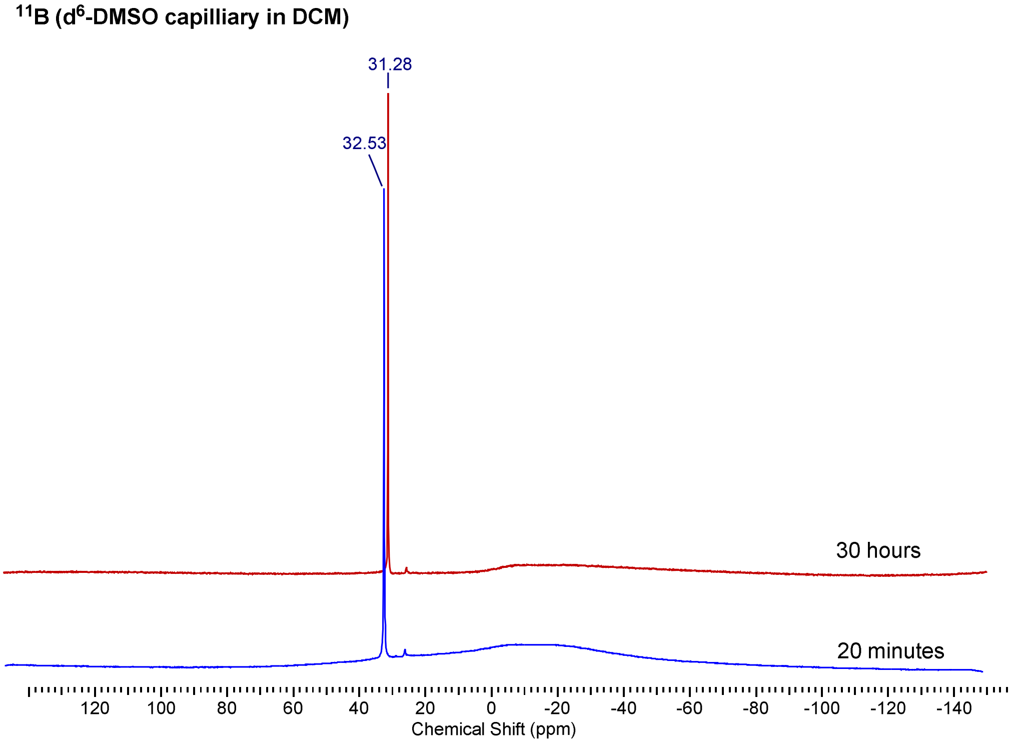

In Situ Spectra prior to esterification to form (4a)

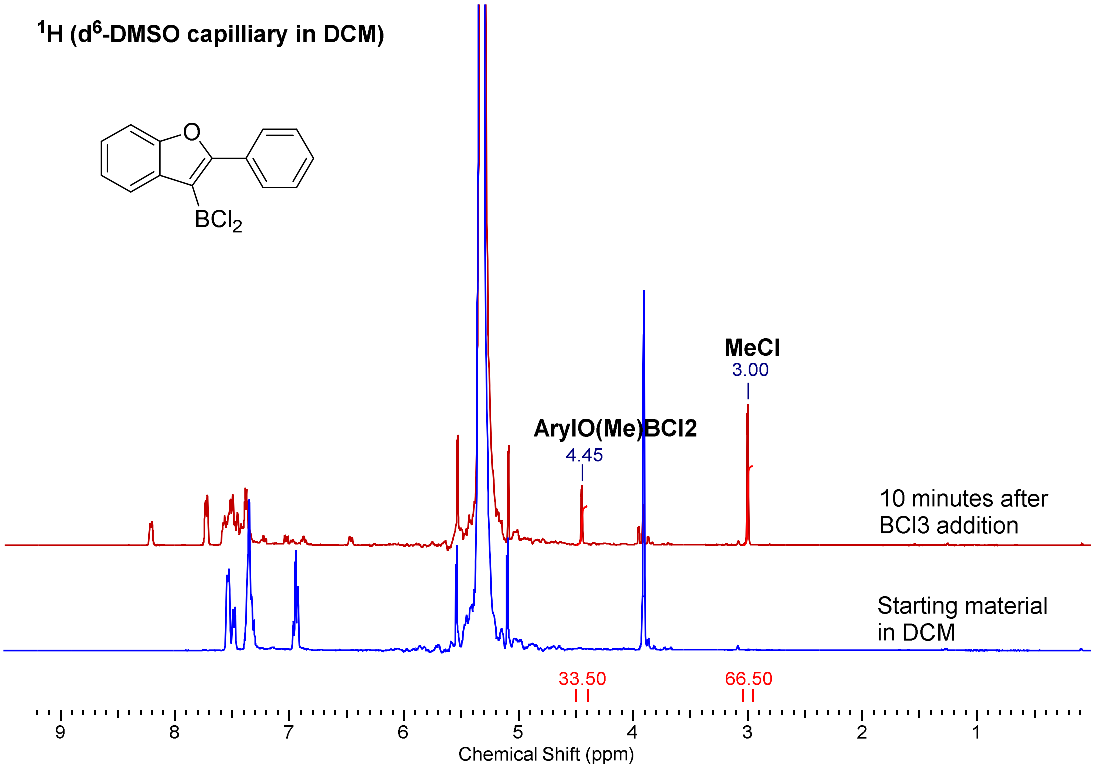

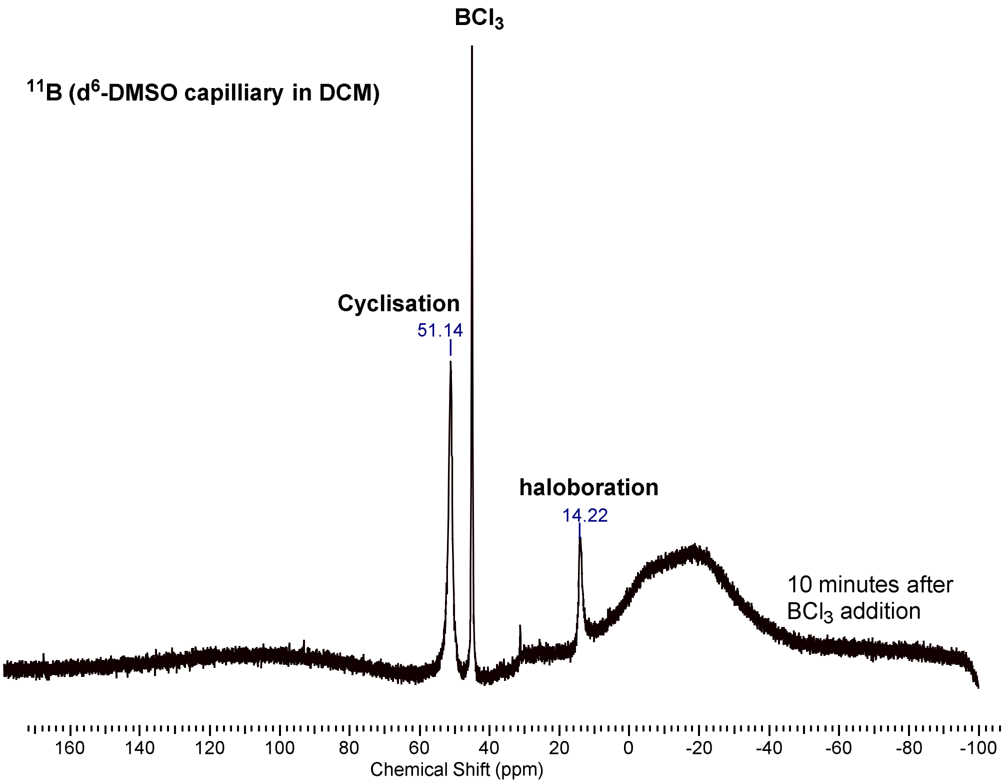

Transhaloboration / Demethylation with 1,2-bis(2-methoxyphenyl)ethyne

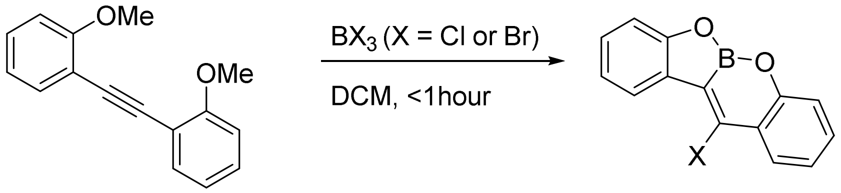

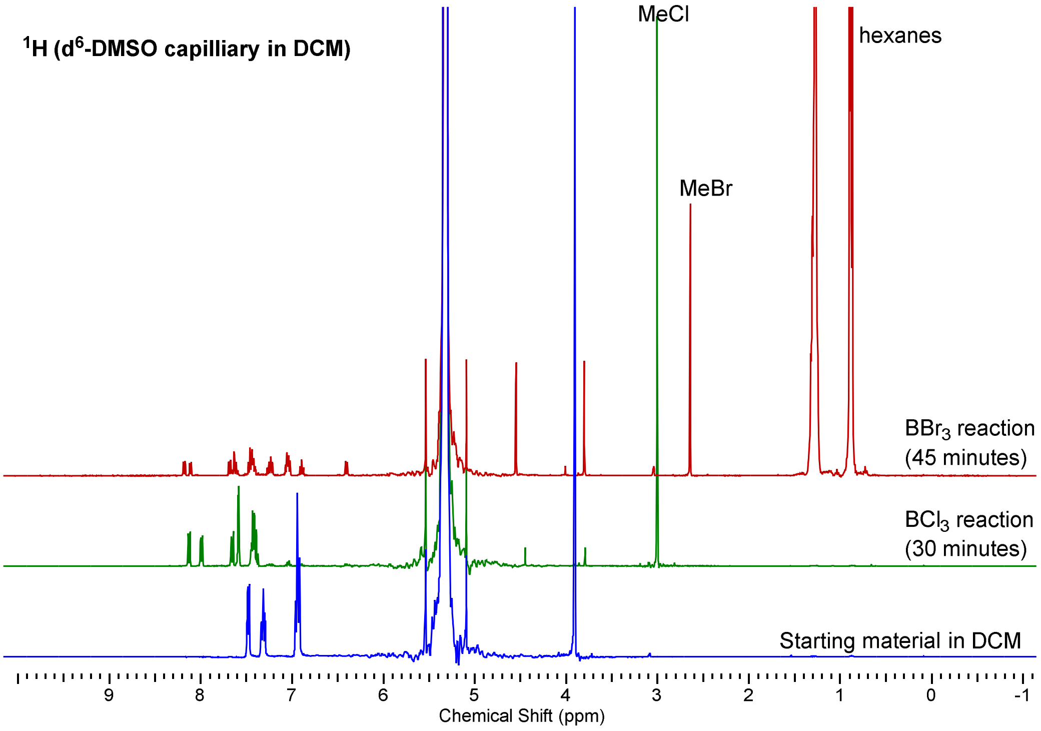

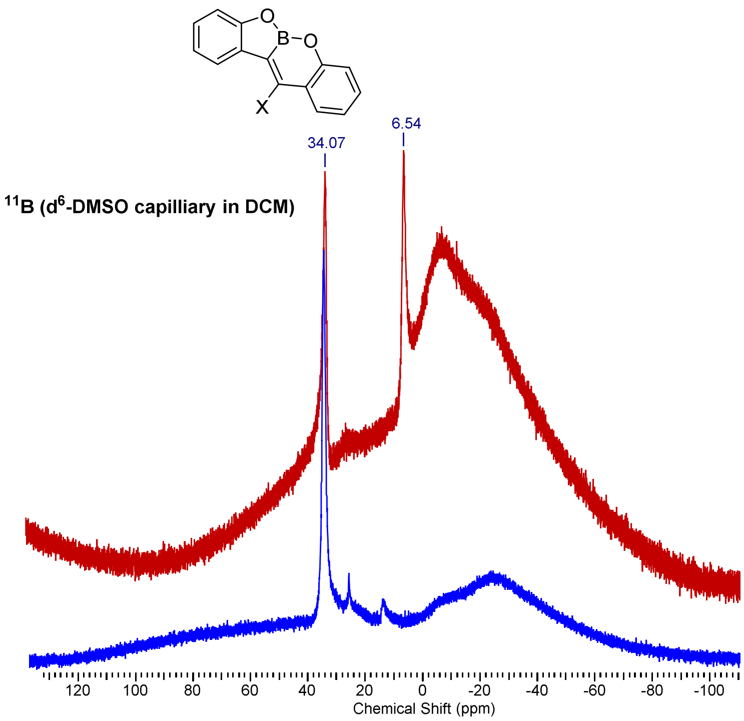

The δ_{11B} 34 ppm species is fully consistent with a VinylB(OR)$_2$ species. The similar chemical shifts using BCl_3 and BBr_3 supports similar products being made consistent with trans-haloboration / demethylation. The formation of benzofuranBX$_2$ (X = Br or Cl) species would lead to different ^{11}B chemical shifts. Furthermore the reaction with BCl_3 results in MeCl being the major product consistent with 2 demethylation steps. The other species observed using BBr3 is currently unknown.

In situ Oxygen-directed trans-haloboration using 3i

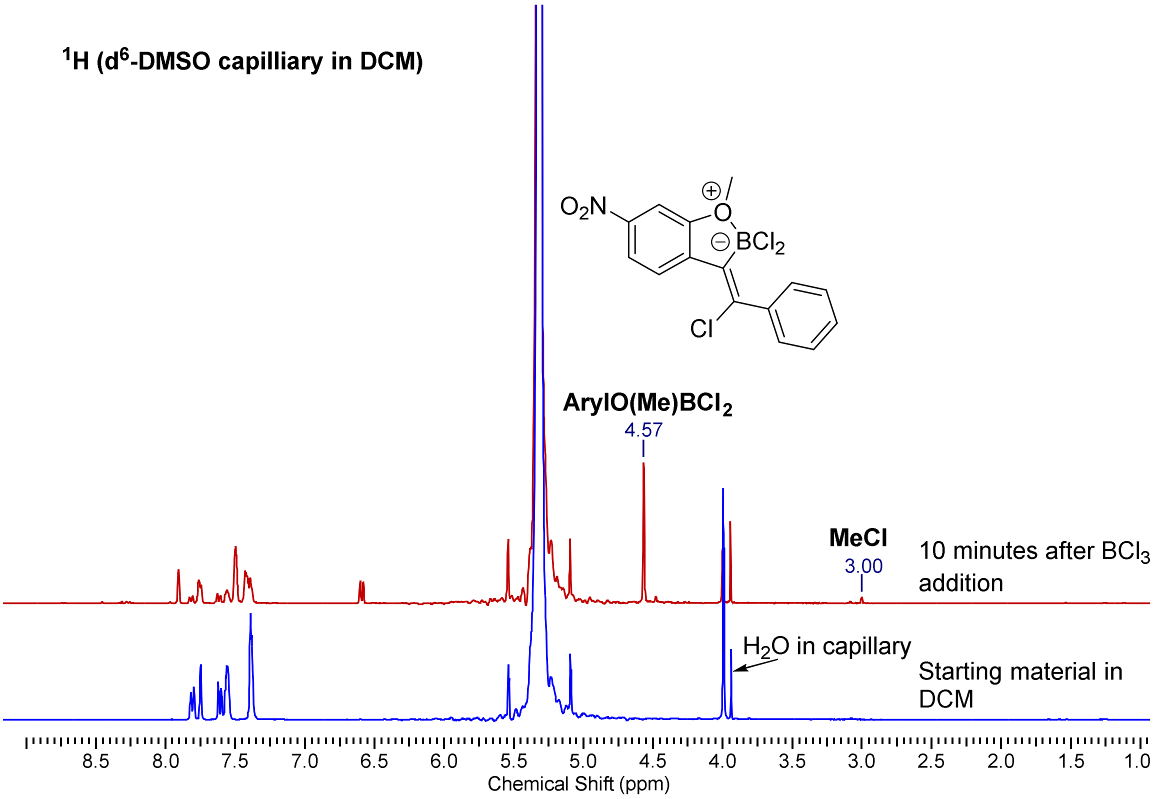

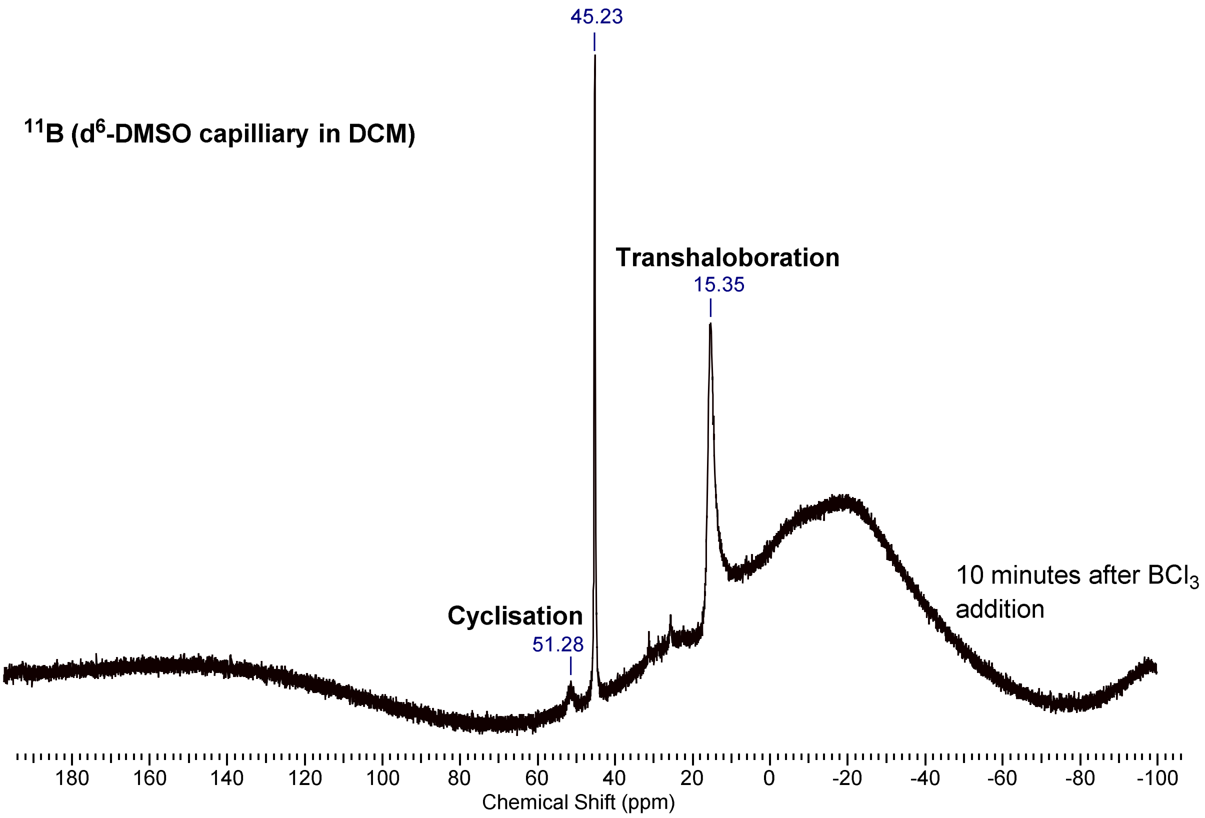

In situ Oxygen-directed trans-haloboration using 3j

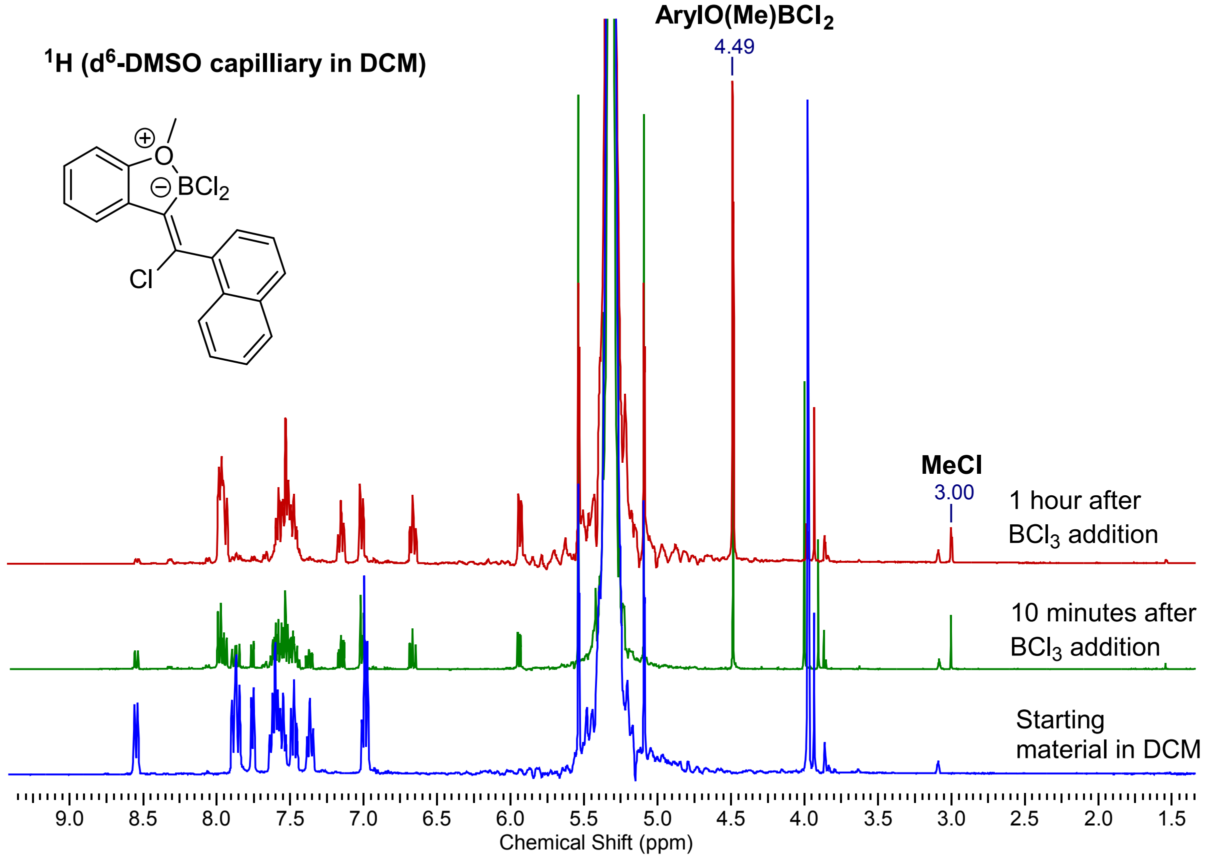

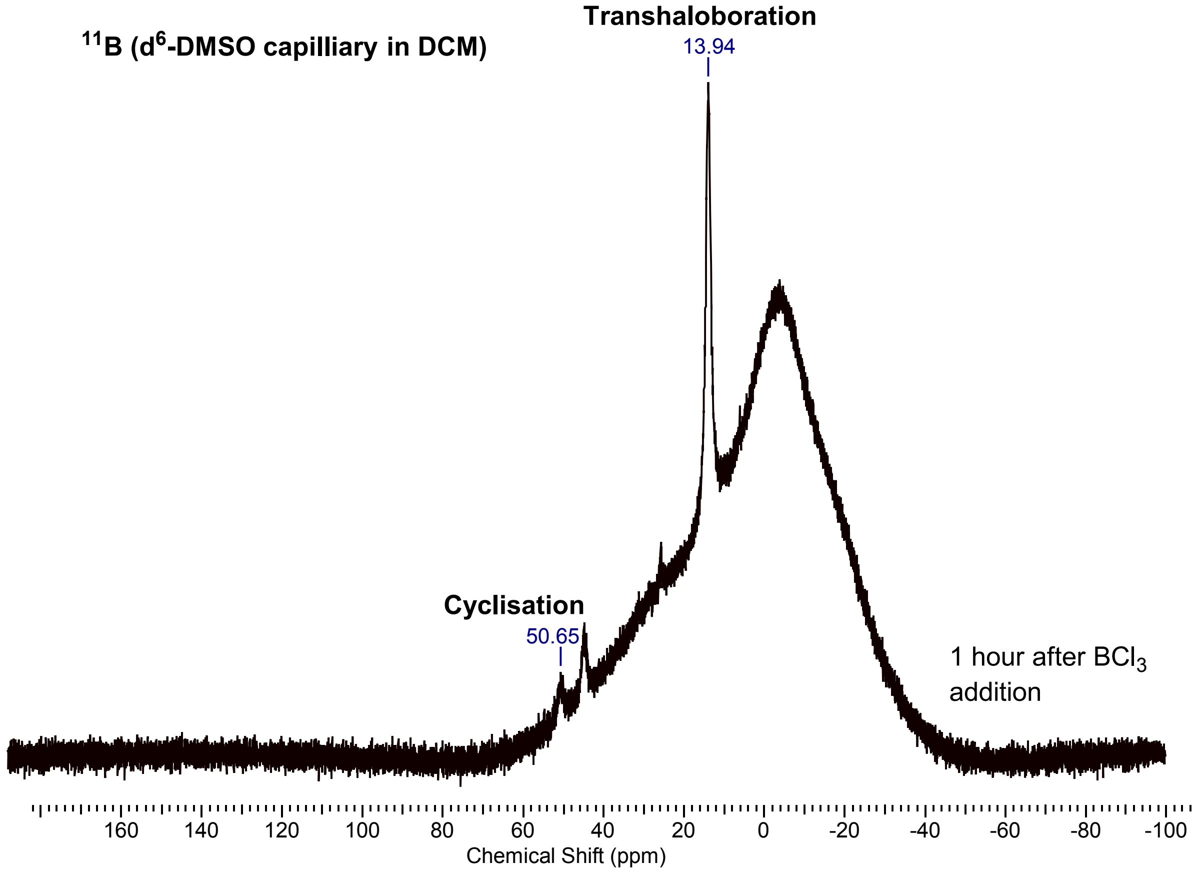

In situ Thioanisole + BCl_3 after 30 minutes- no MeCl formed.

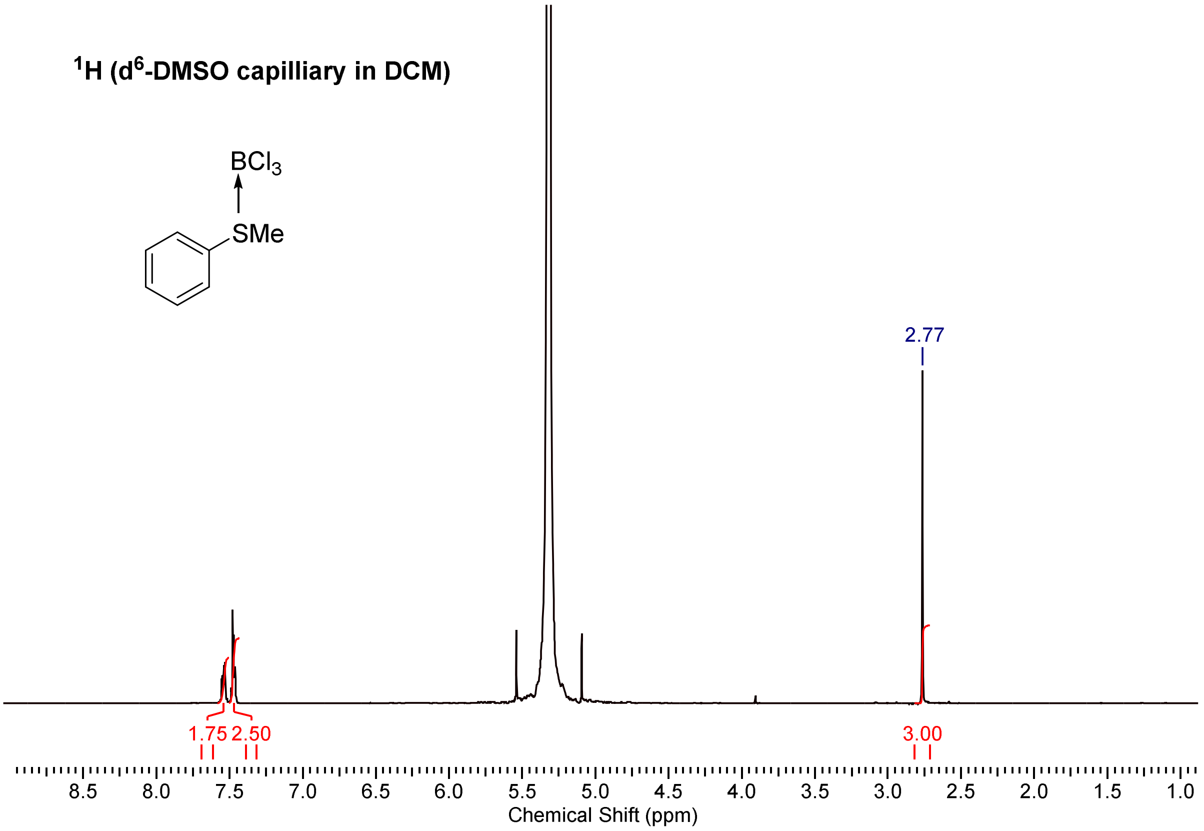

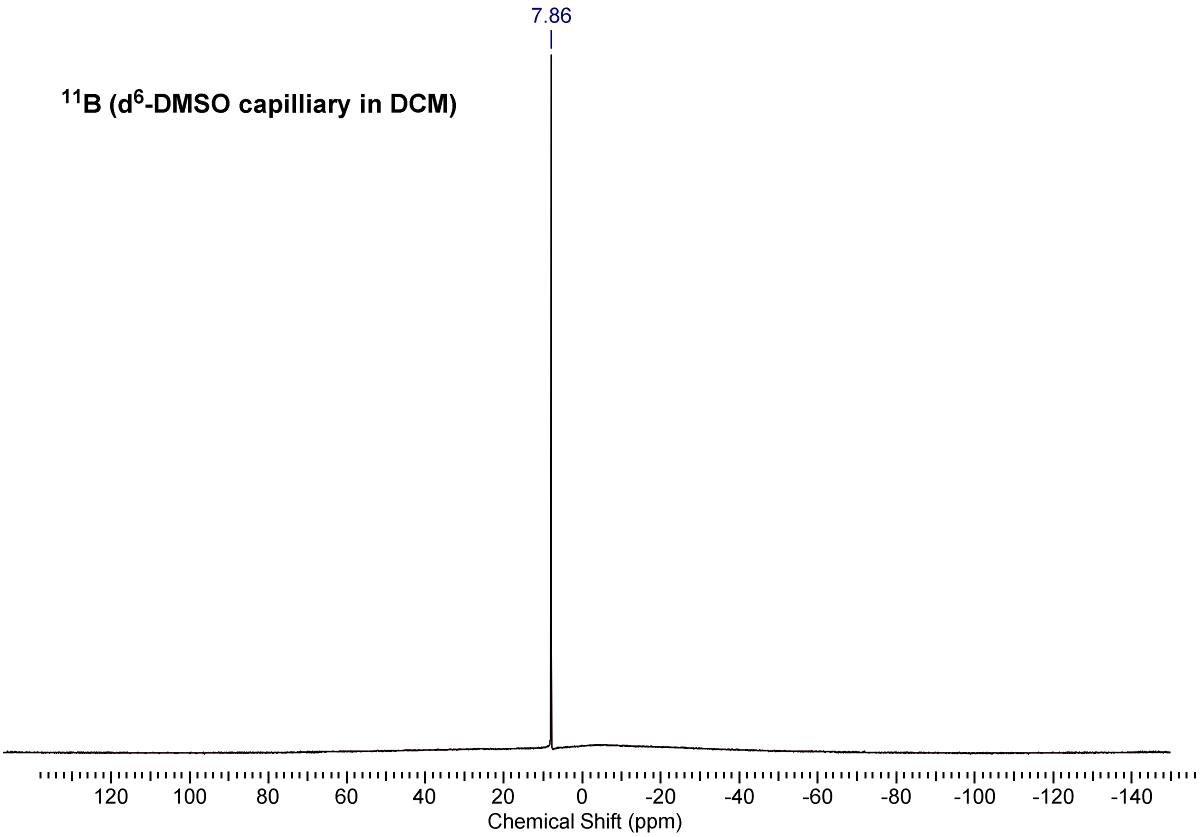

In situ reaction of NEt_3/$AlCl_3$ procedure to generate Methyl(2-(phenylethynyl)phenyl)sulfane (11a)

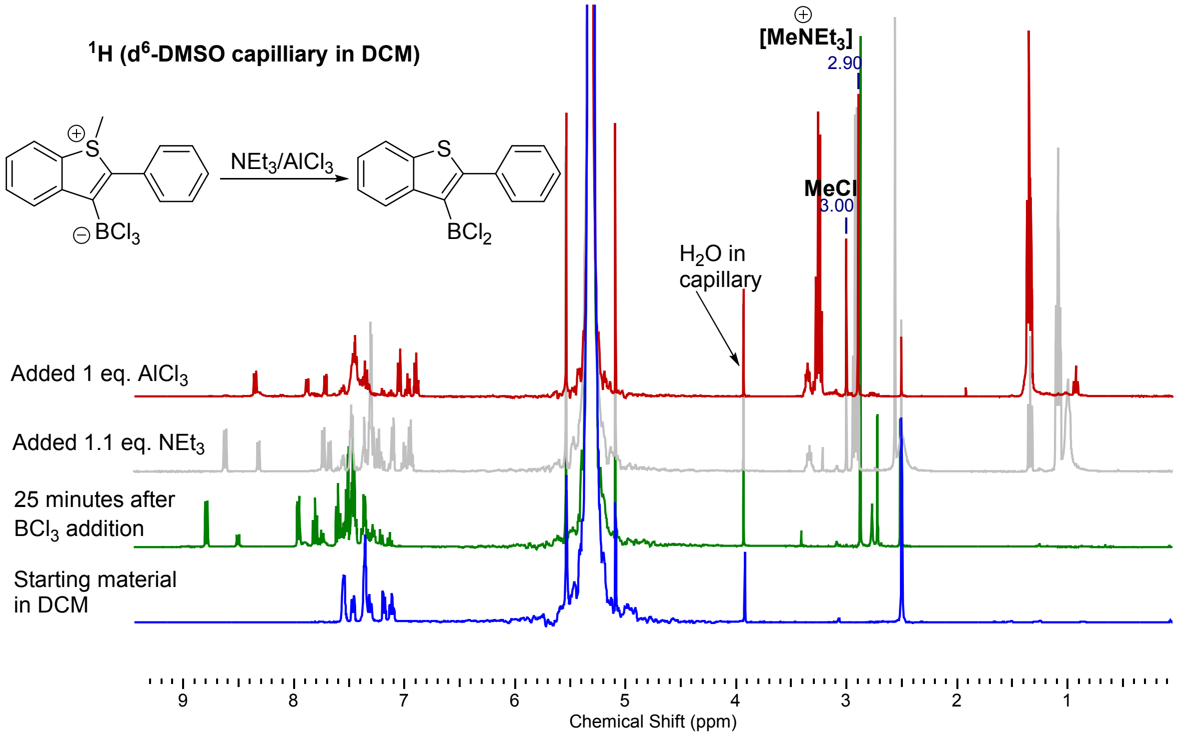

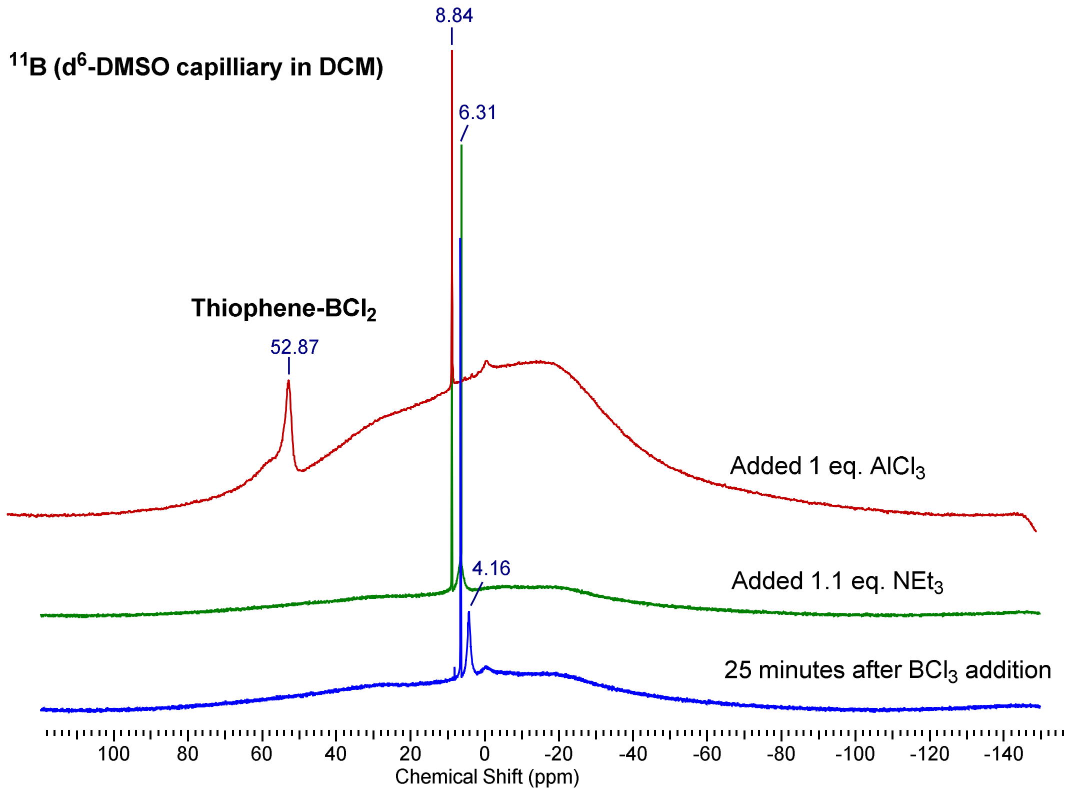

The shoulder at 55 ppm is tentatively attributed [benzothienylBCl(NEt_3)]$^+$.

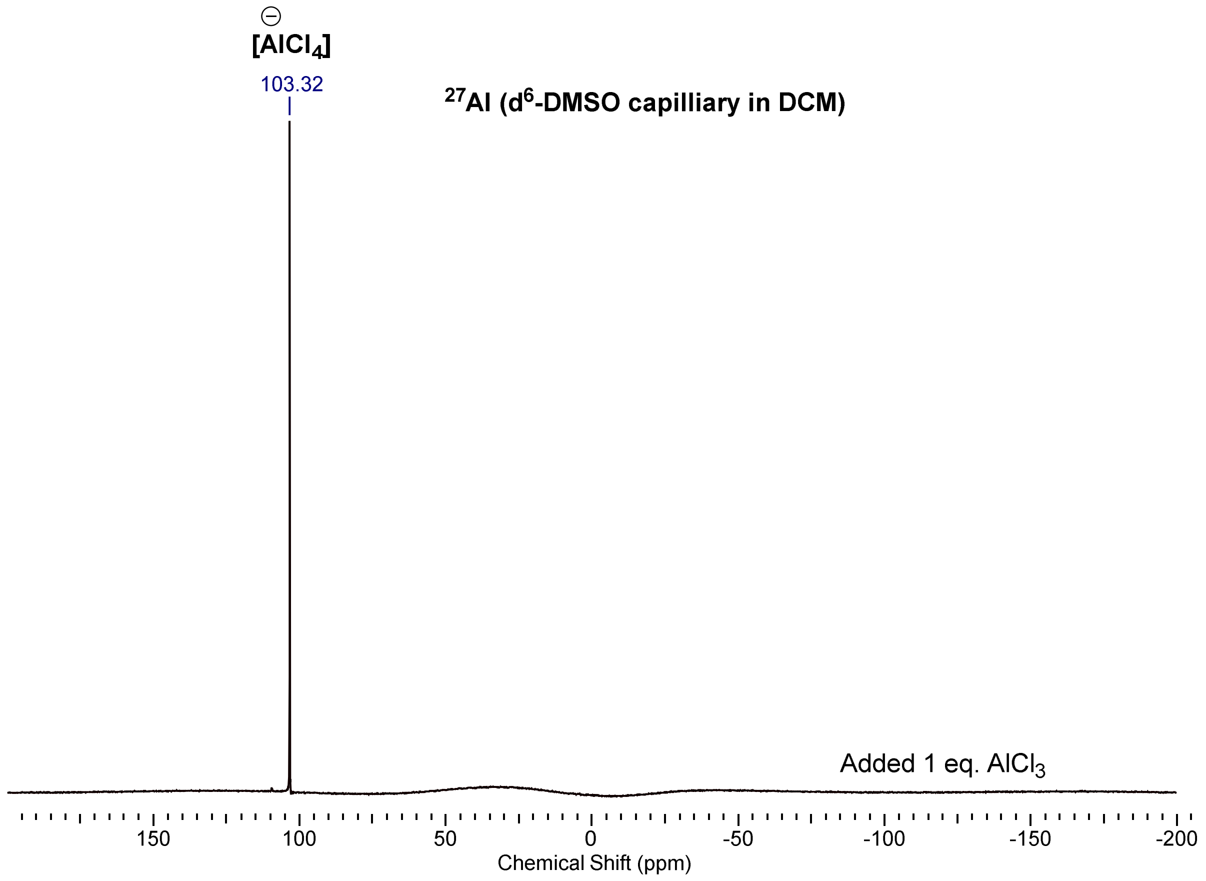

⊖
[AlCl4]
103.32
27Al (d6-DMSO capilliary in DCM)
Added 1 eq. AlCl3
150
100
50
0
-50
-100
-150
-200
Chemical Shift (ppm)

In situ reaction of $AlCl_3/NEt_3$ procedure to generate Methyl(2-(phenylethynyl)phenyl)sulfane (11a)

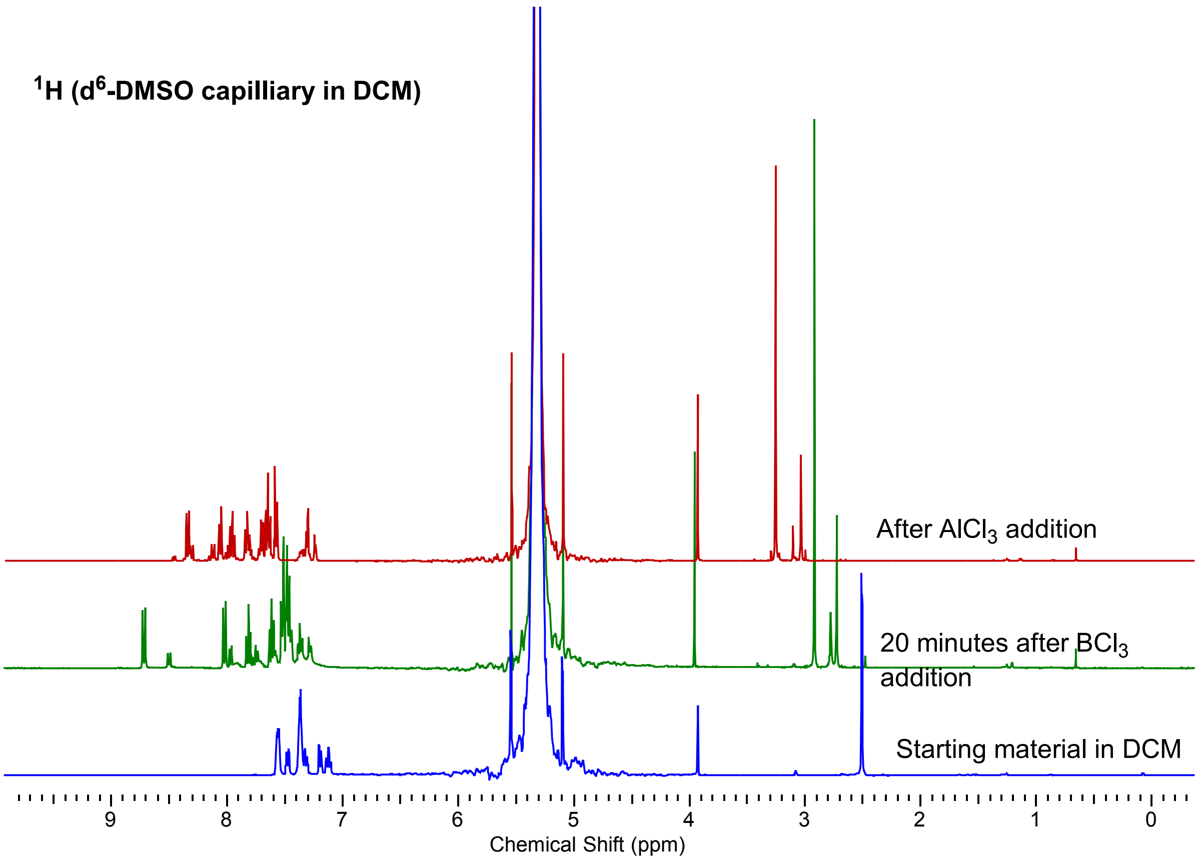

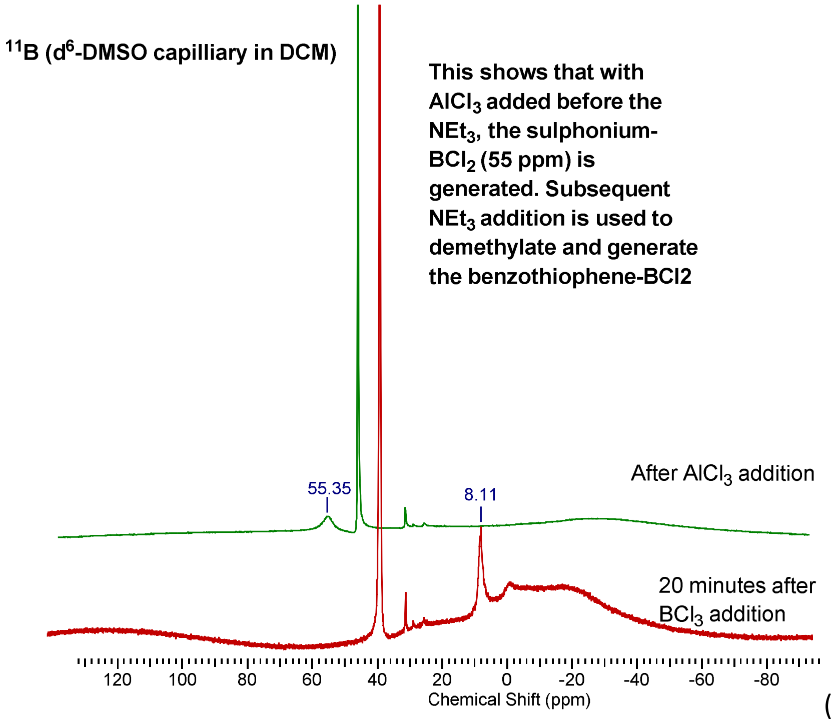

(NB the other resonance in the green spectra is BCl_3)

^{27}Al (d^6-DMSO capilliary in DCM)

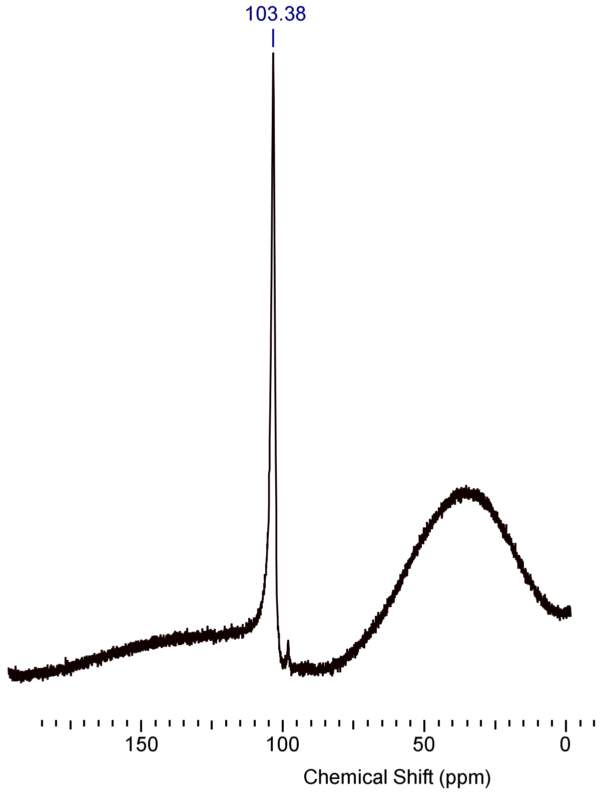

11. Spectral Data on Isolated Compounds

N,N-dimethyl-2-(phenylethynyl)aniline **(1)**

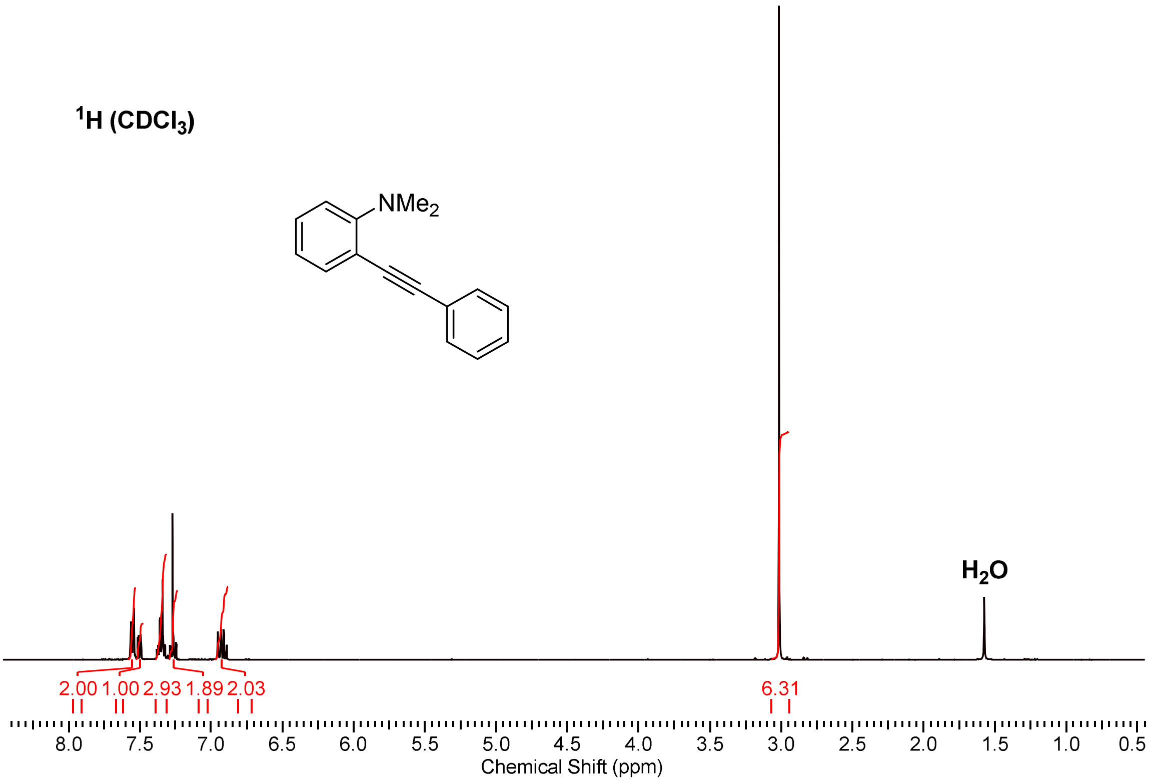

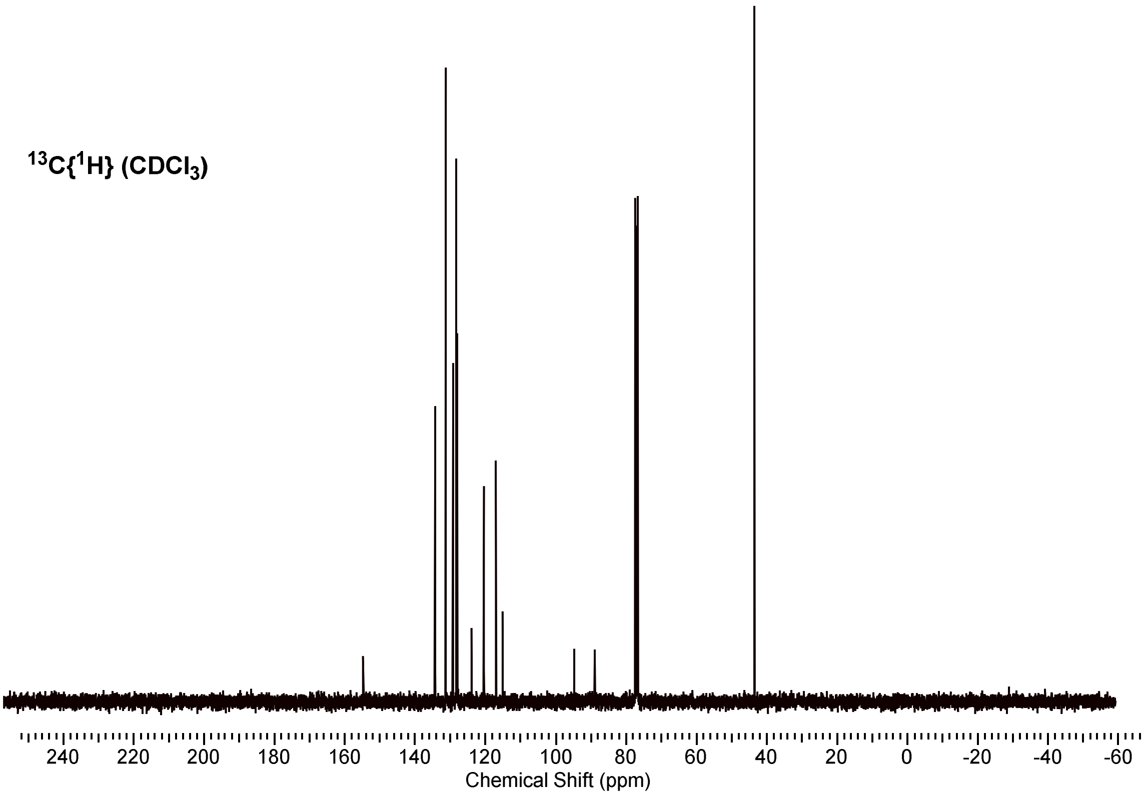

Nitrogen-directed *trans*-haloboration **(2)**

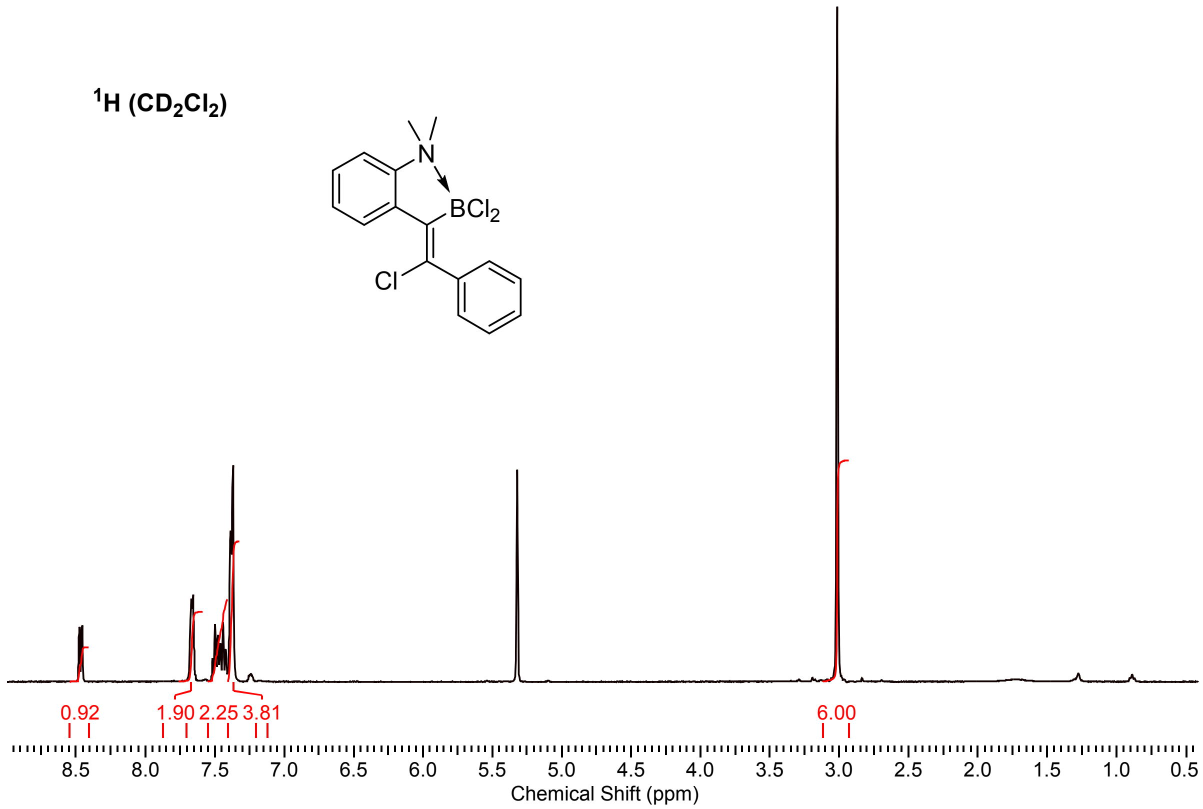

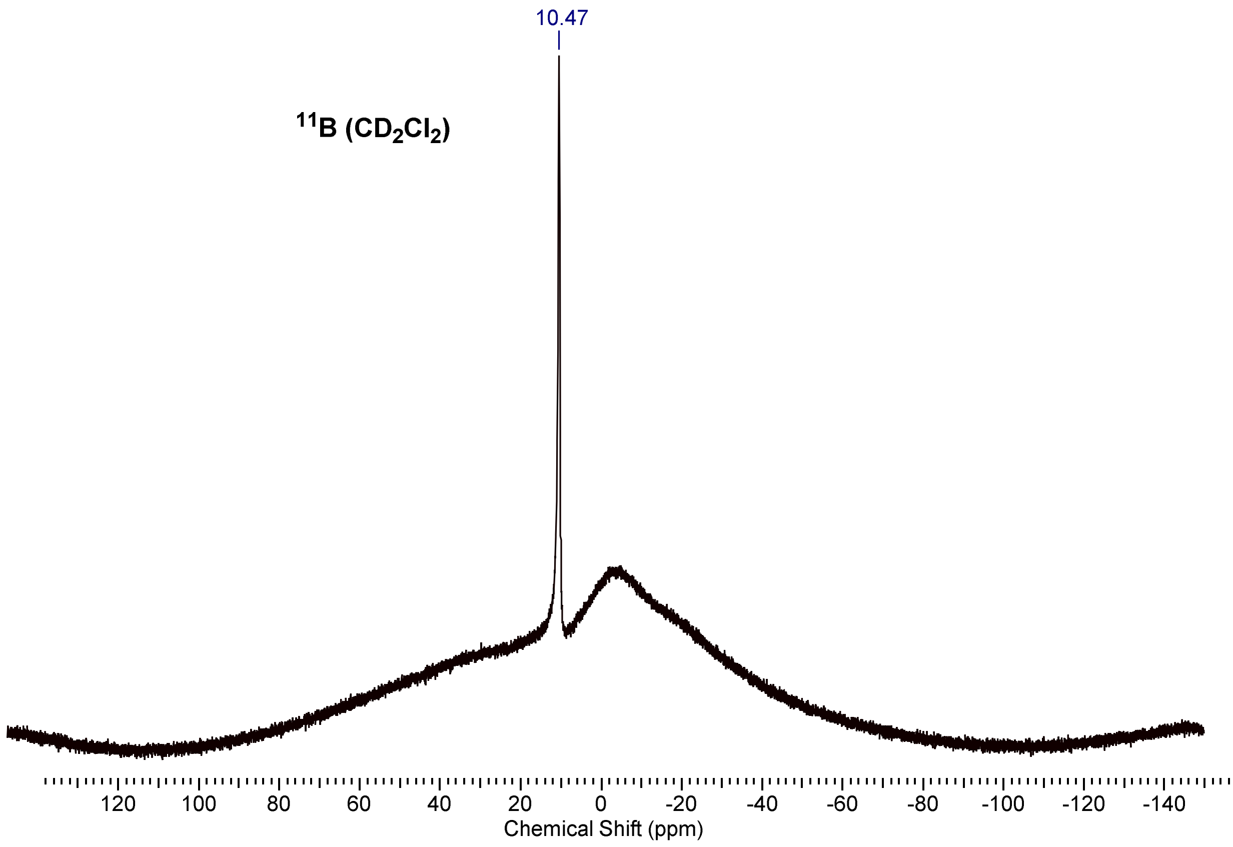

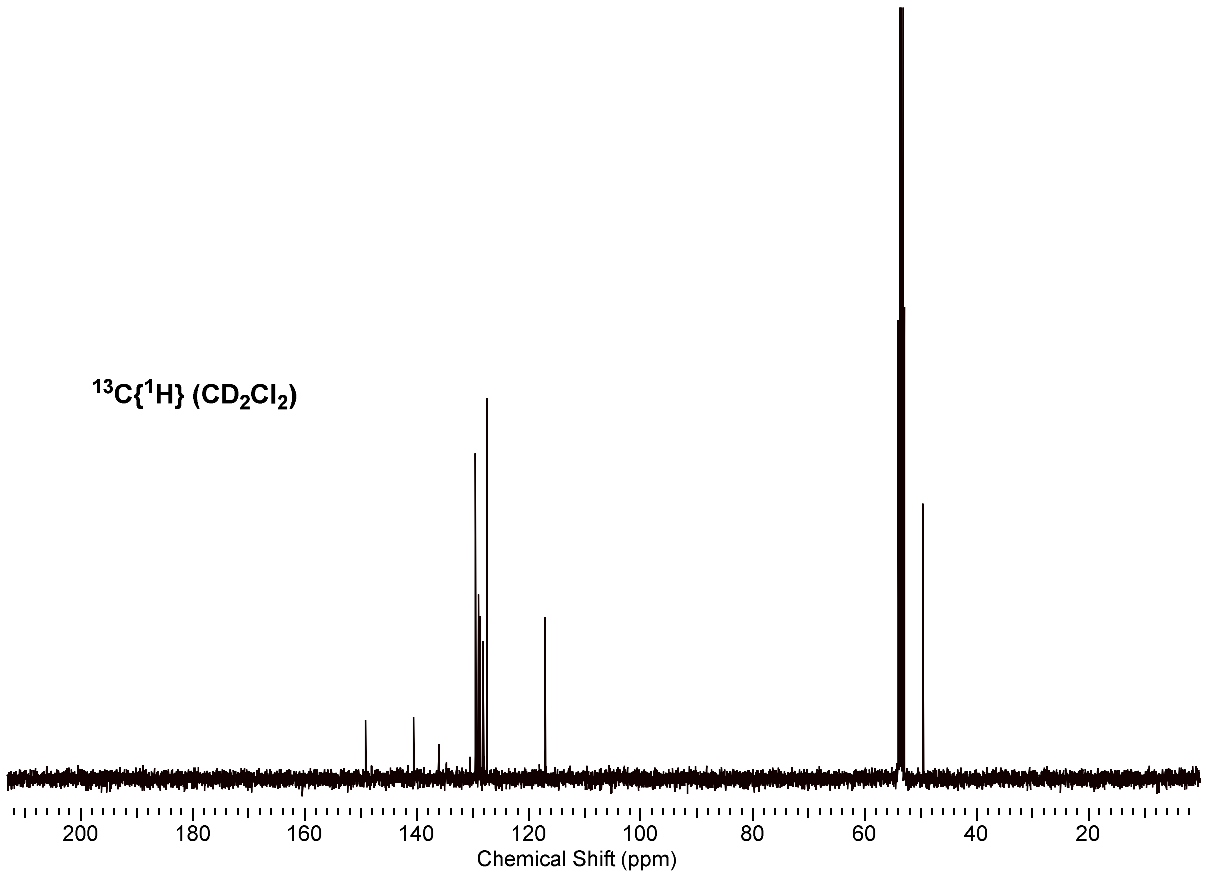

$^{13}C\{^1H\}$ (CD_2Cl_2)
200
180
160
140
120
100
80
60
40
20
Chemical Shift (ppm)

1-methoxy-2-(phenylethynyl)benzene **(3a)**

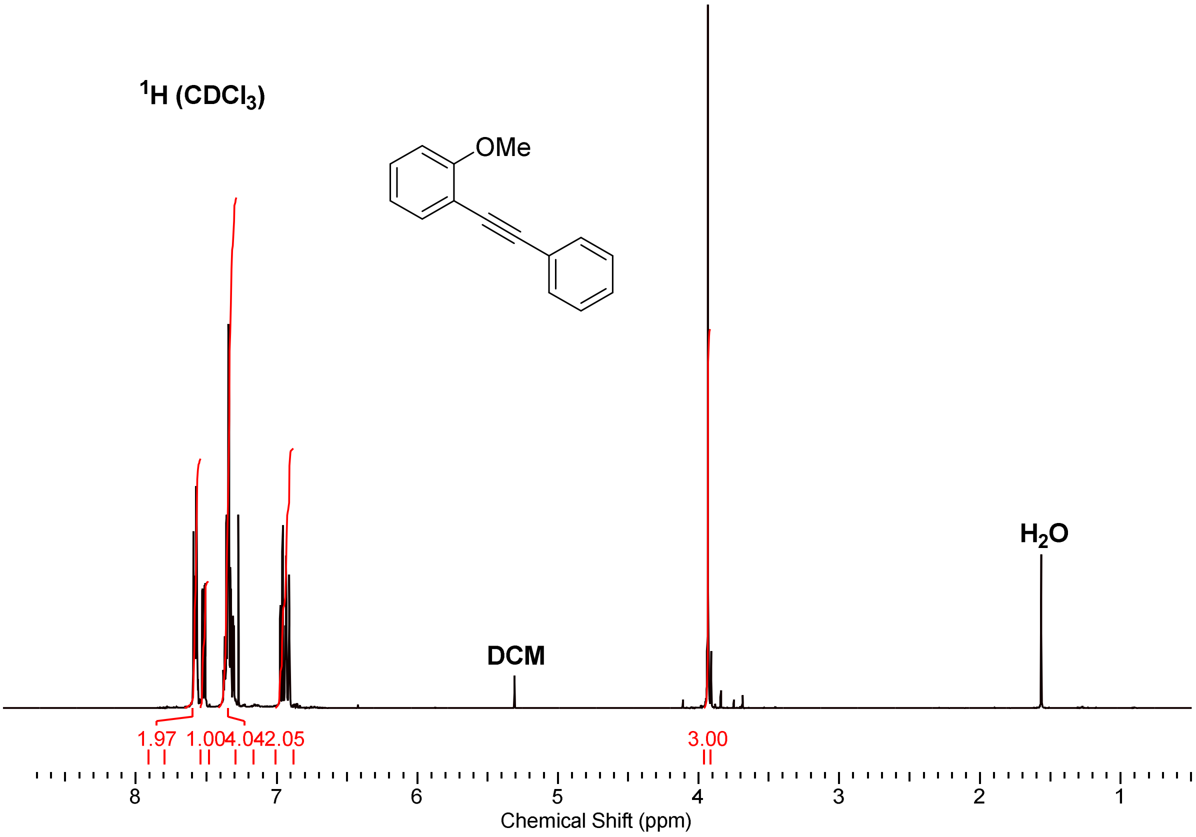

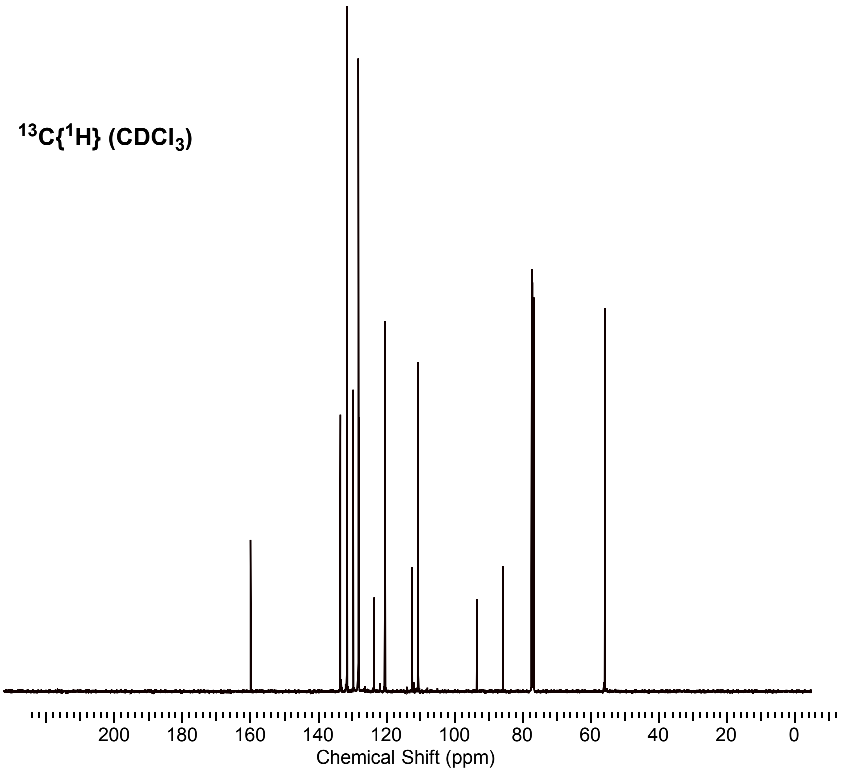

1-methoxy-4-methyl-2-(phenylethynyl)benzene **(3b)**

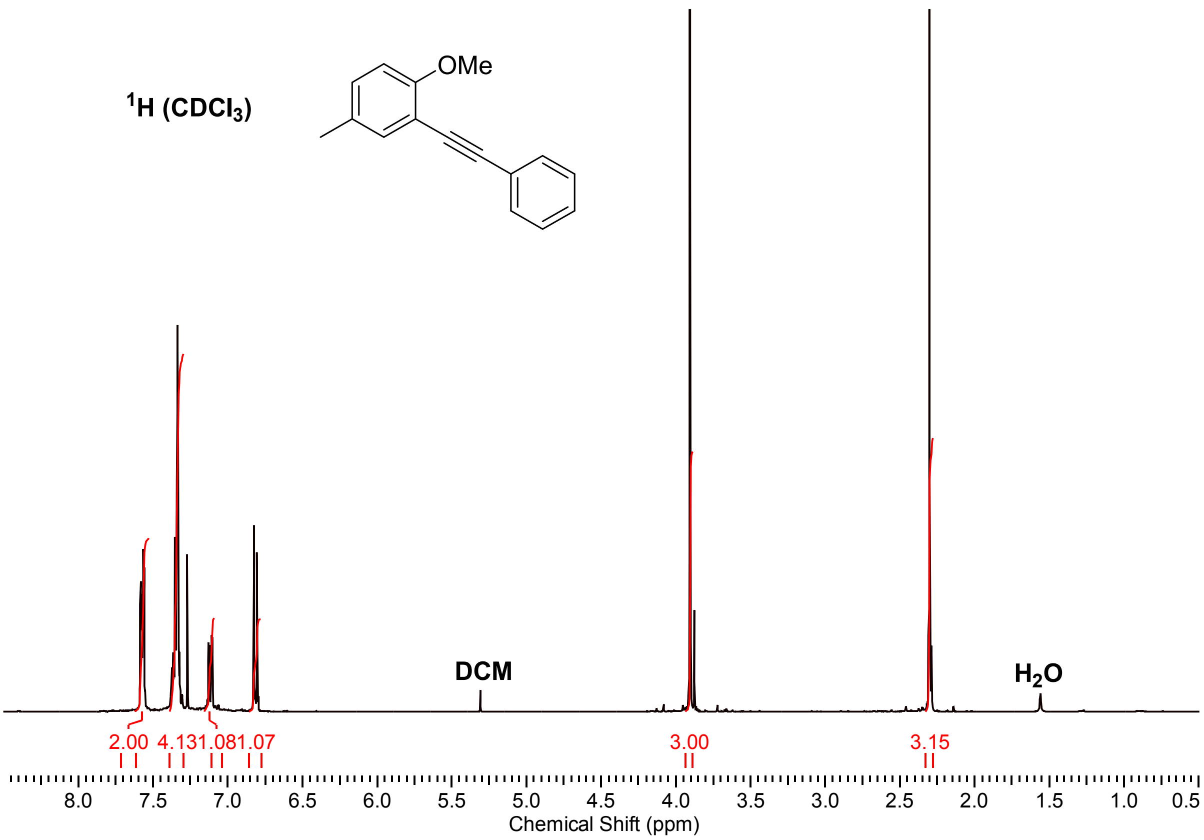

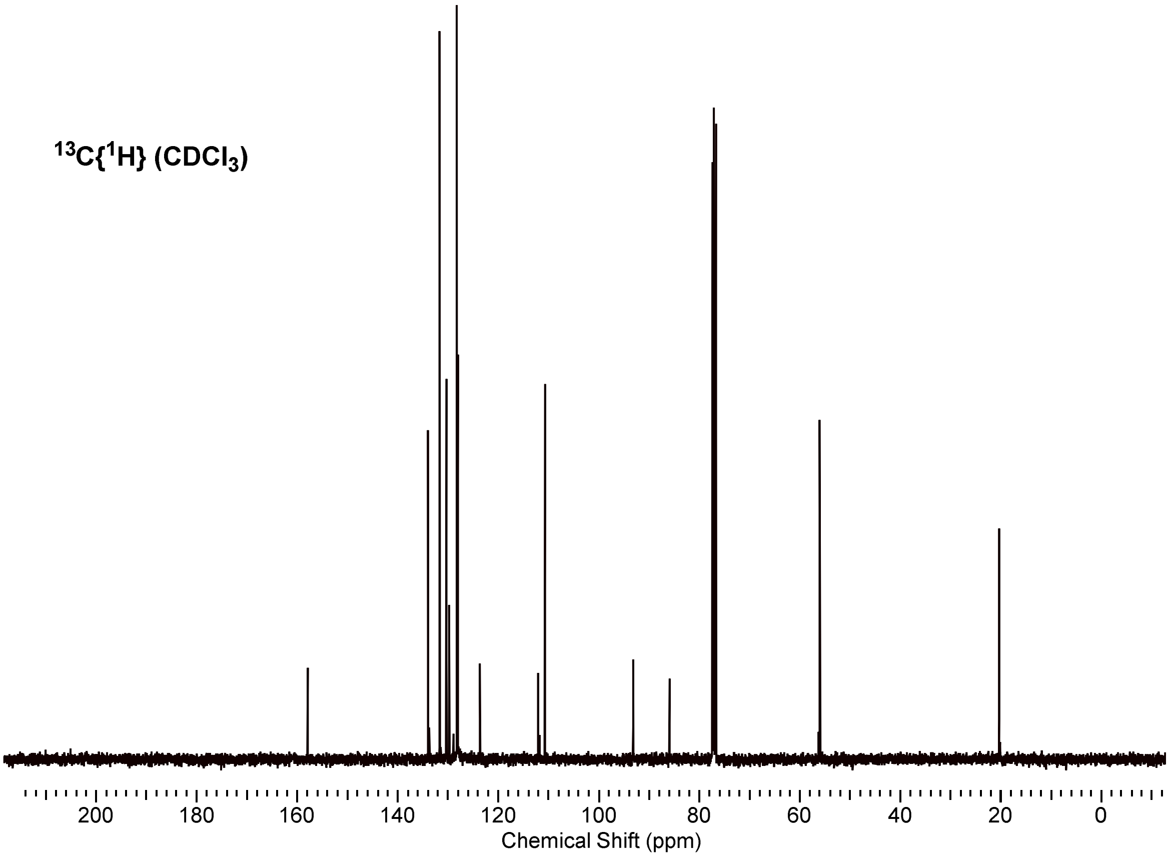

4-chloro-1-methoxy-2-(phenylethynyl)benzene (**3c**)

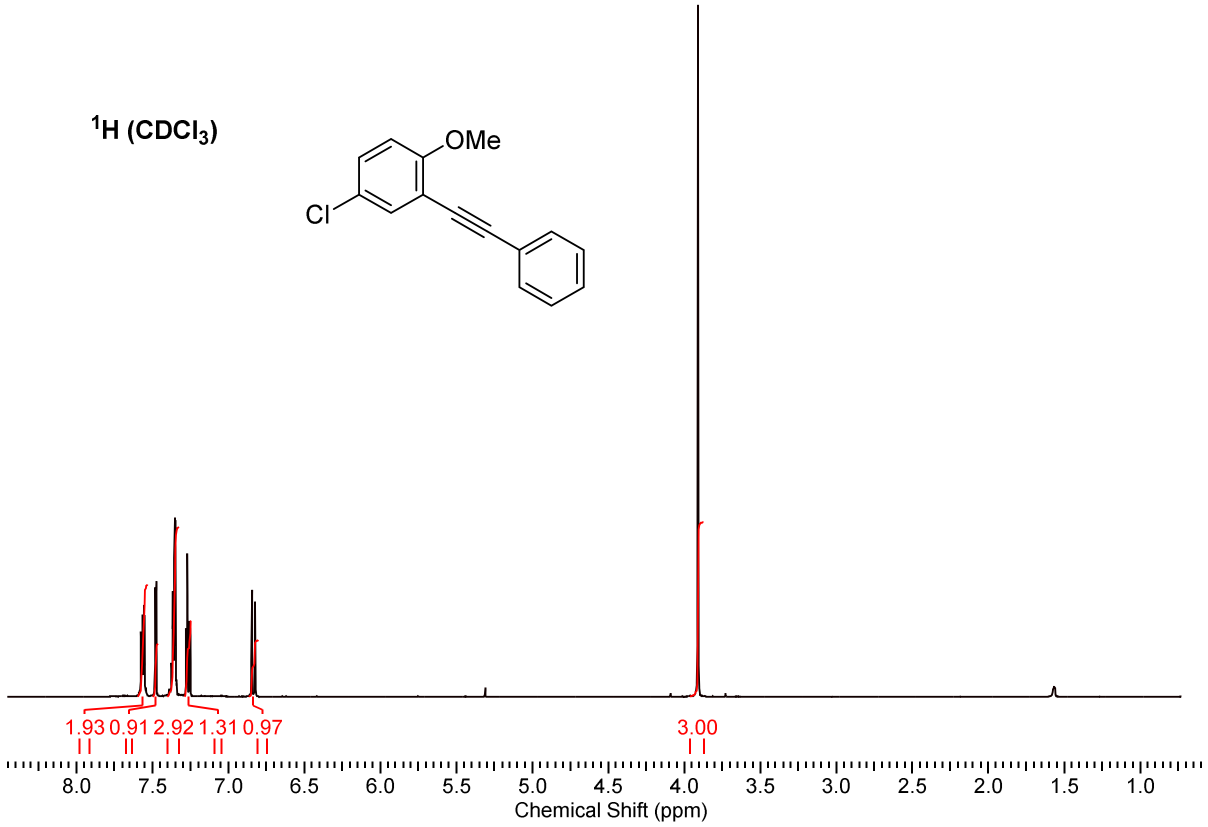

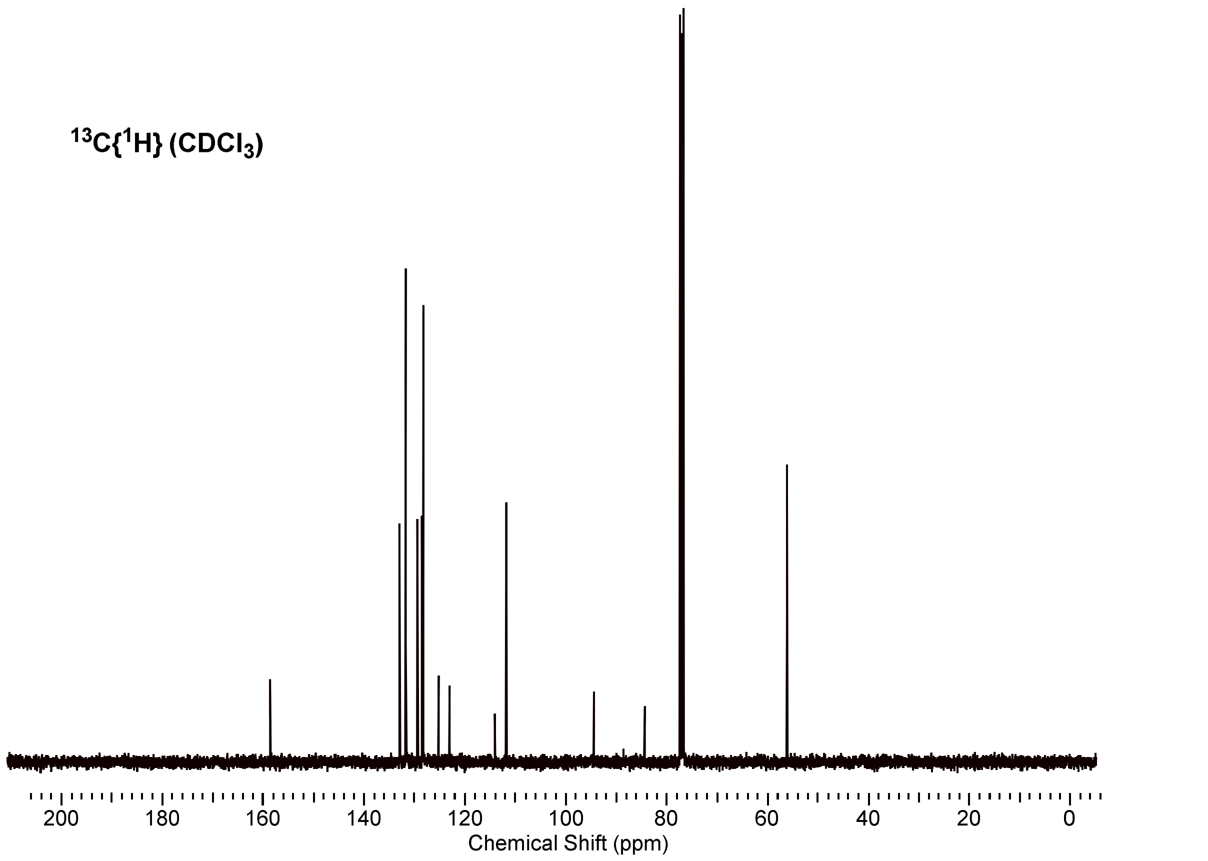

1-fluoro-2-methoxy-3-(phenylethynyl)benzene **(3d)**

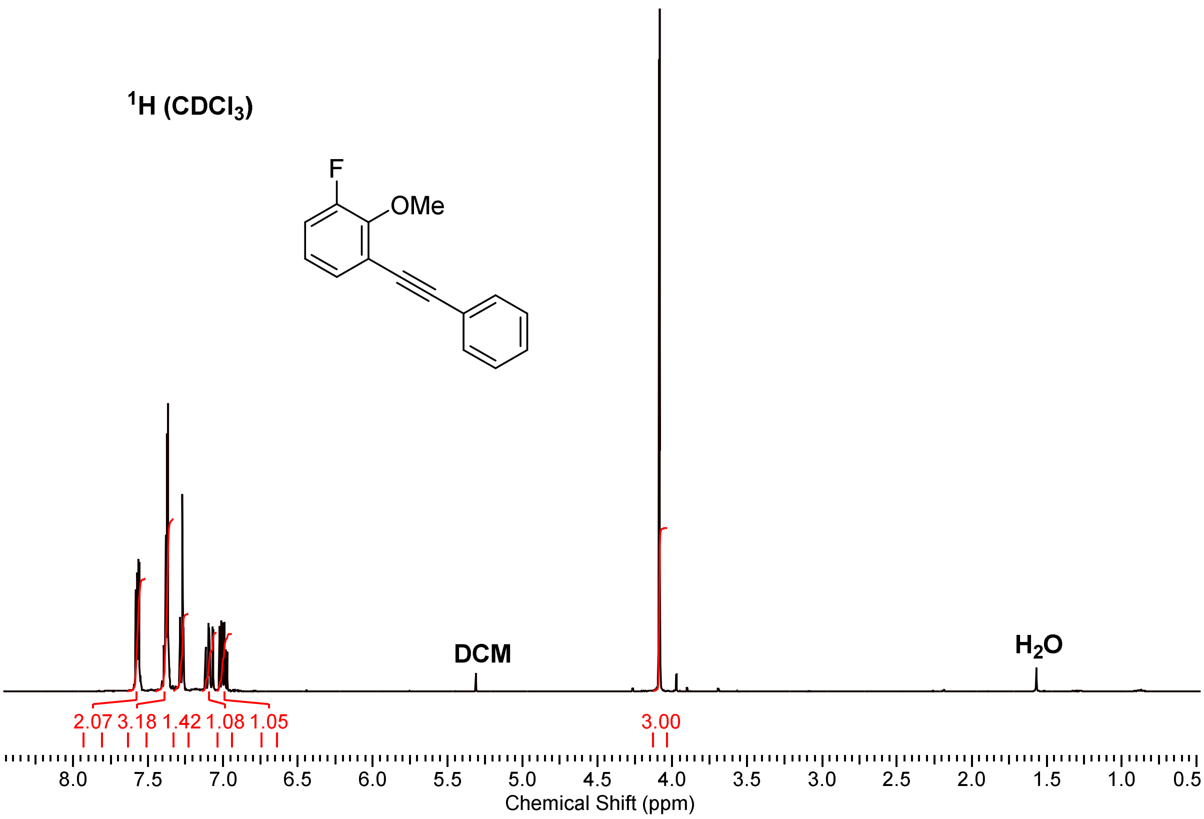

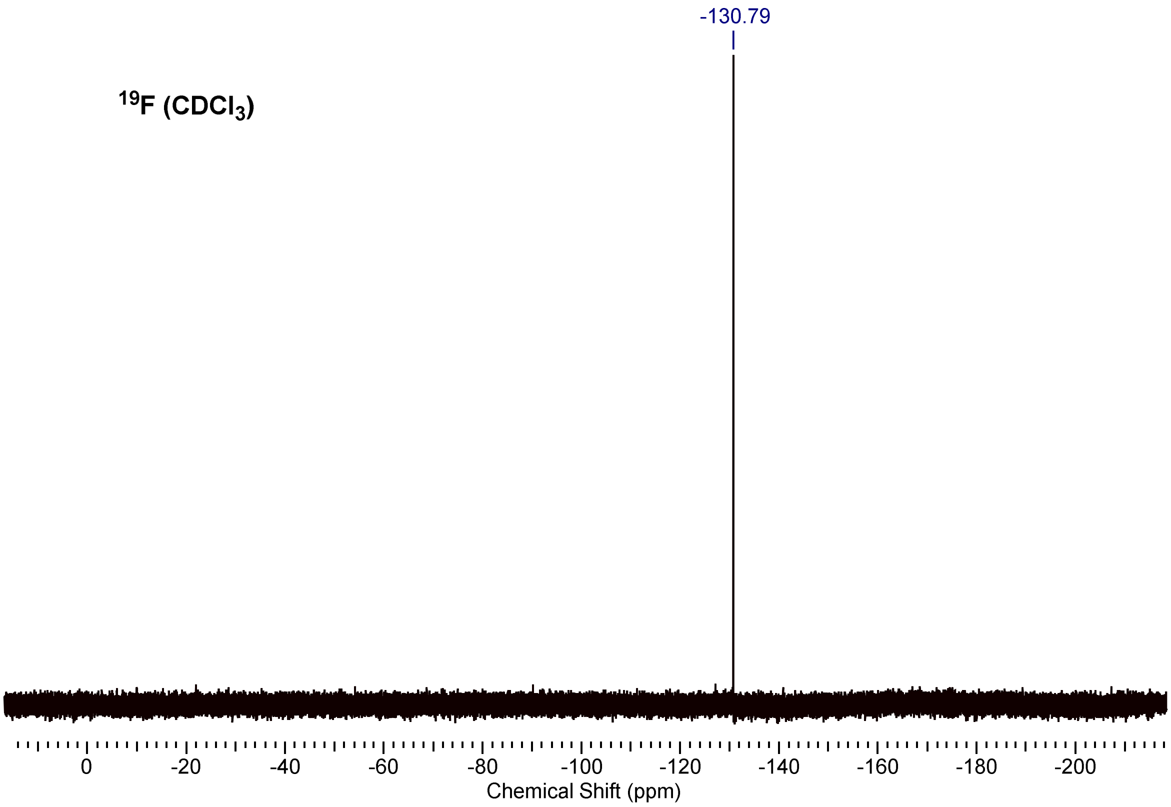

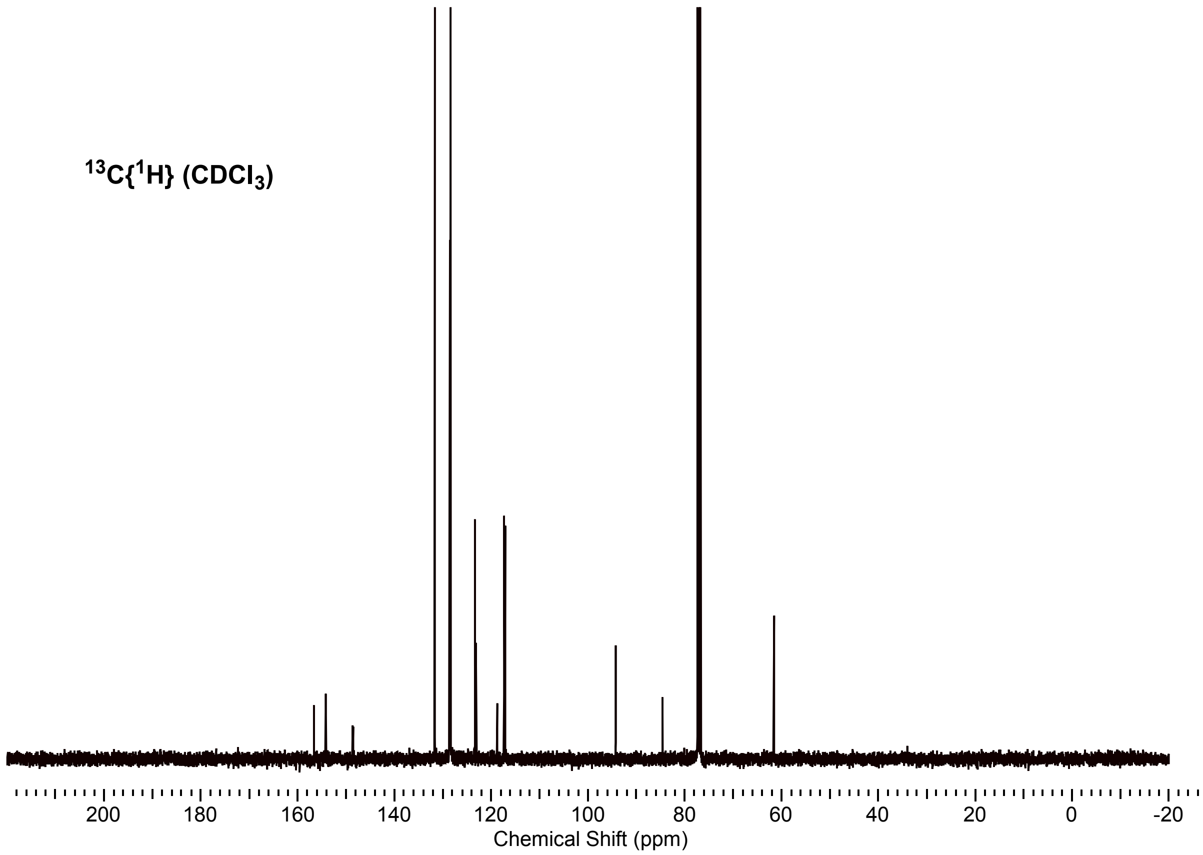

13C{1H} (CDCl3)
200
180
160
140
120
100
80
60
40
20
0
-20
Chemical Shift (ppm)

1-methoxy-3-methyl-2-(phenylethynyl)benzene **(3e)**

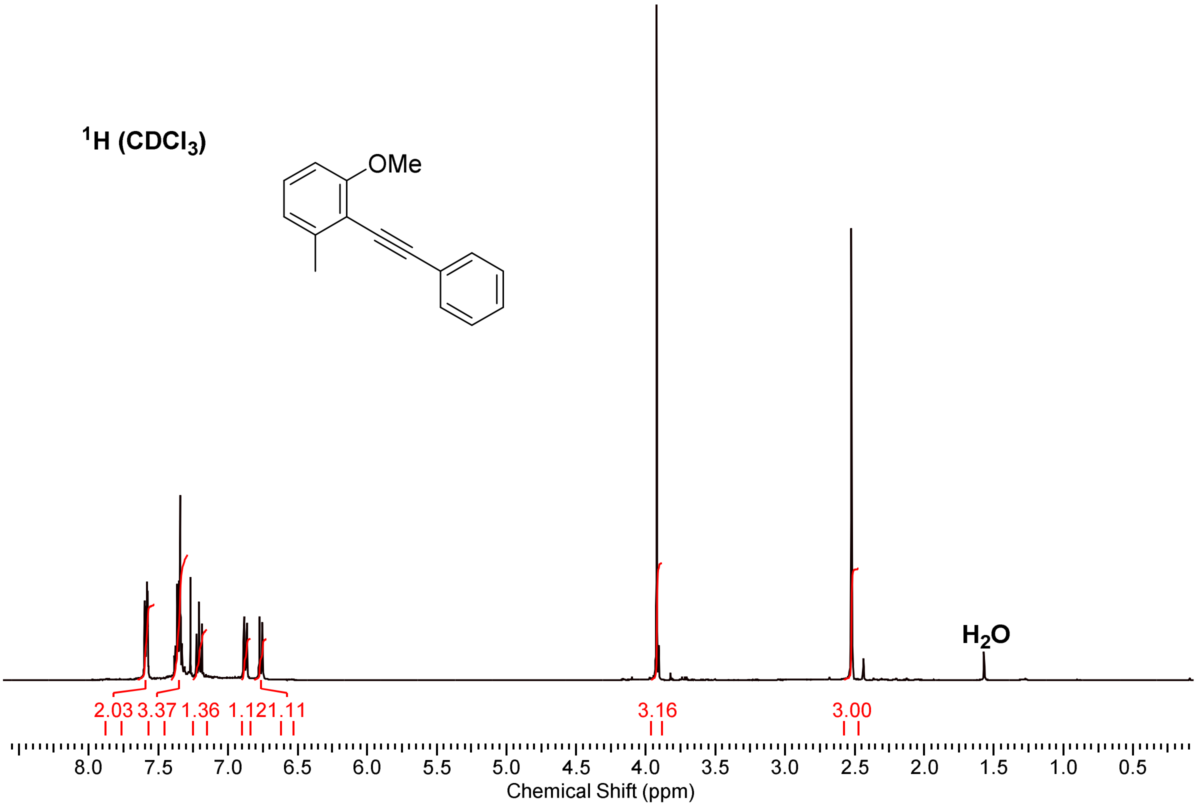

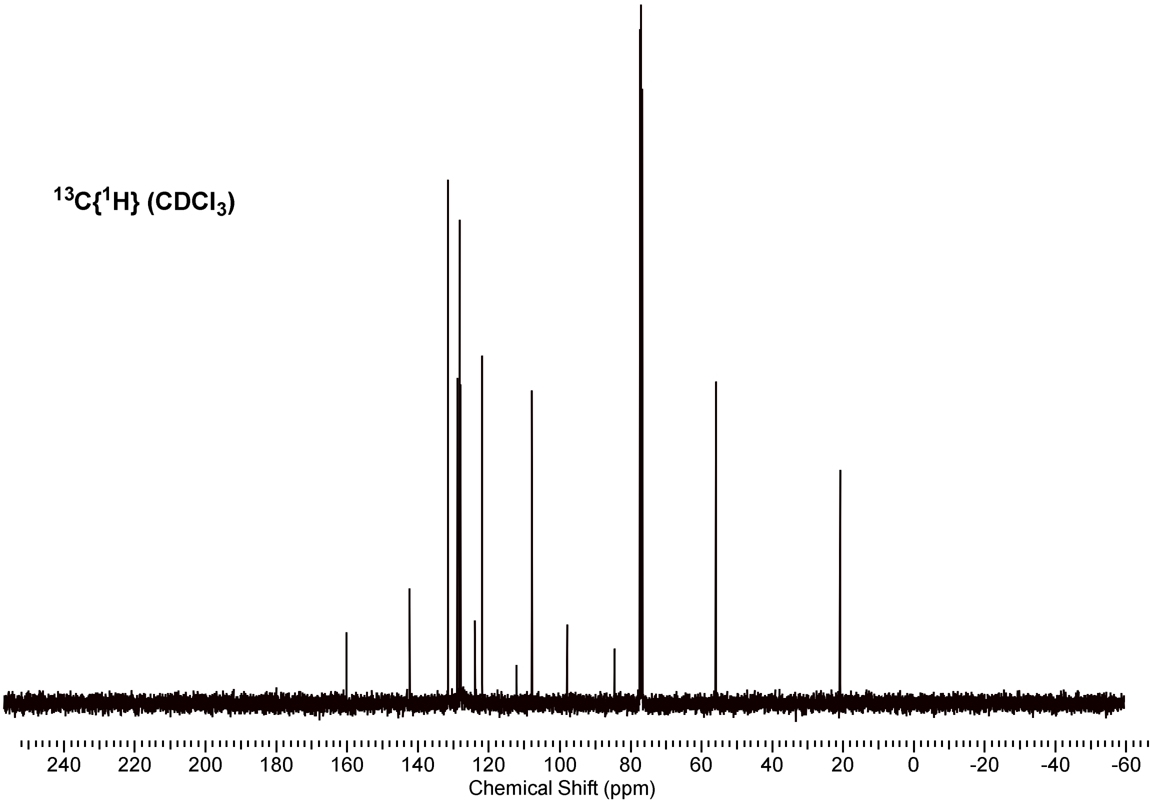

1-methoxy-2-(3-phenylprop-1-yn-1-yl)benzene **(3f)**

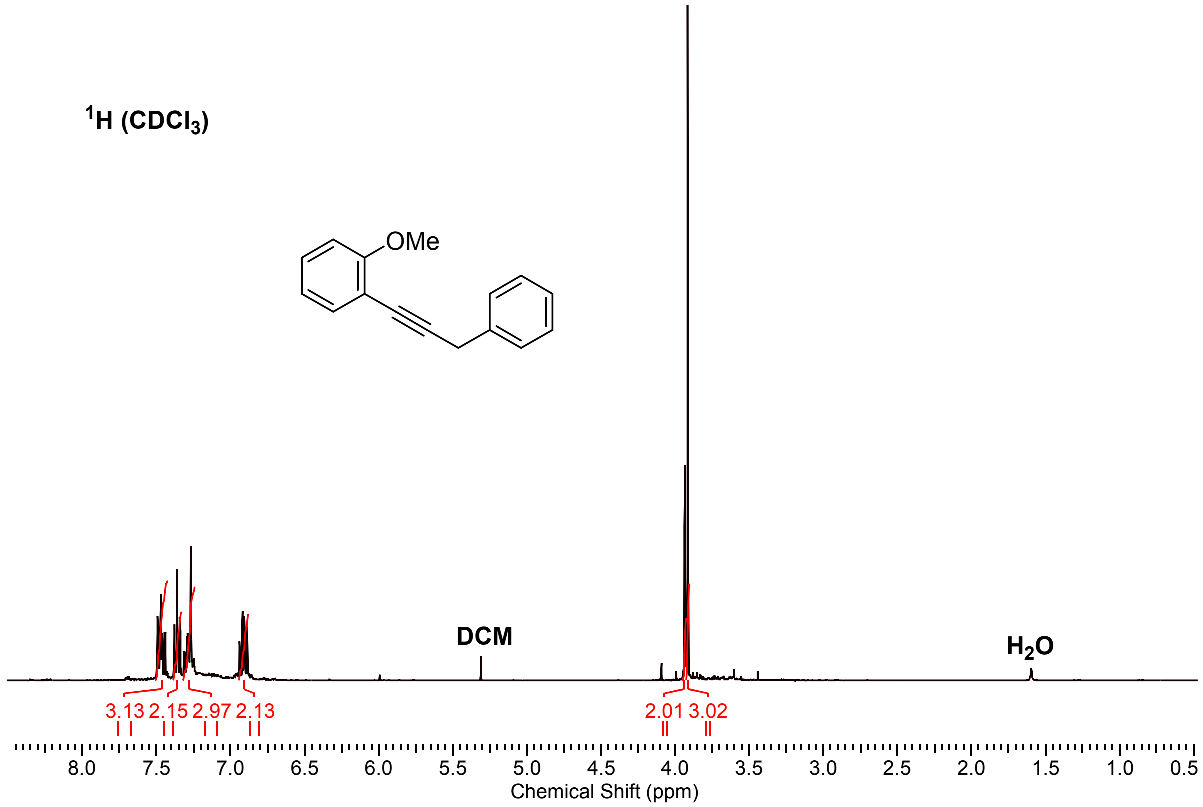

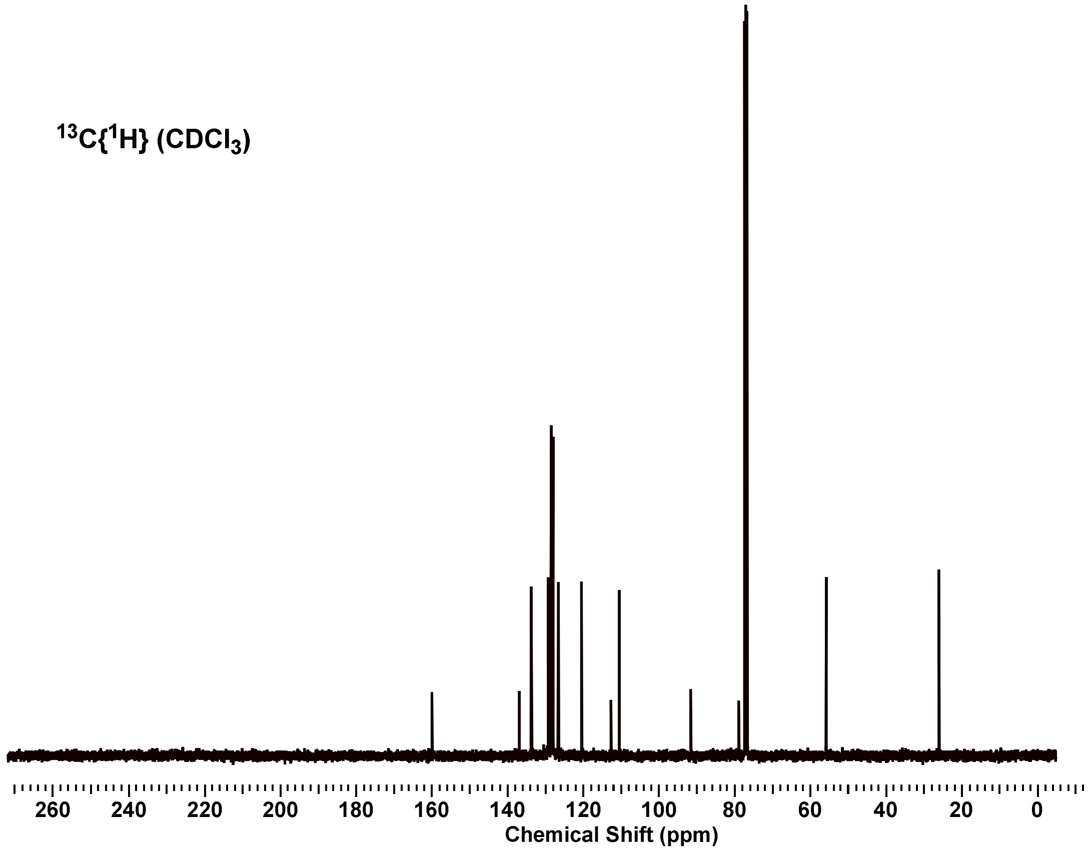

1-methoxy-2-(prop-1-yn-1-yl)benzene **(3g)**

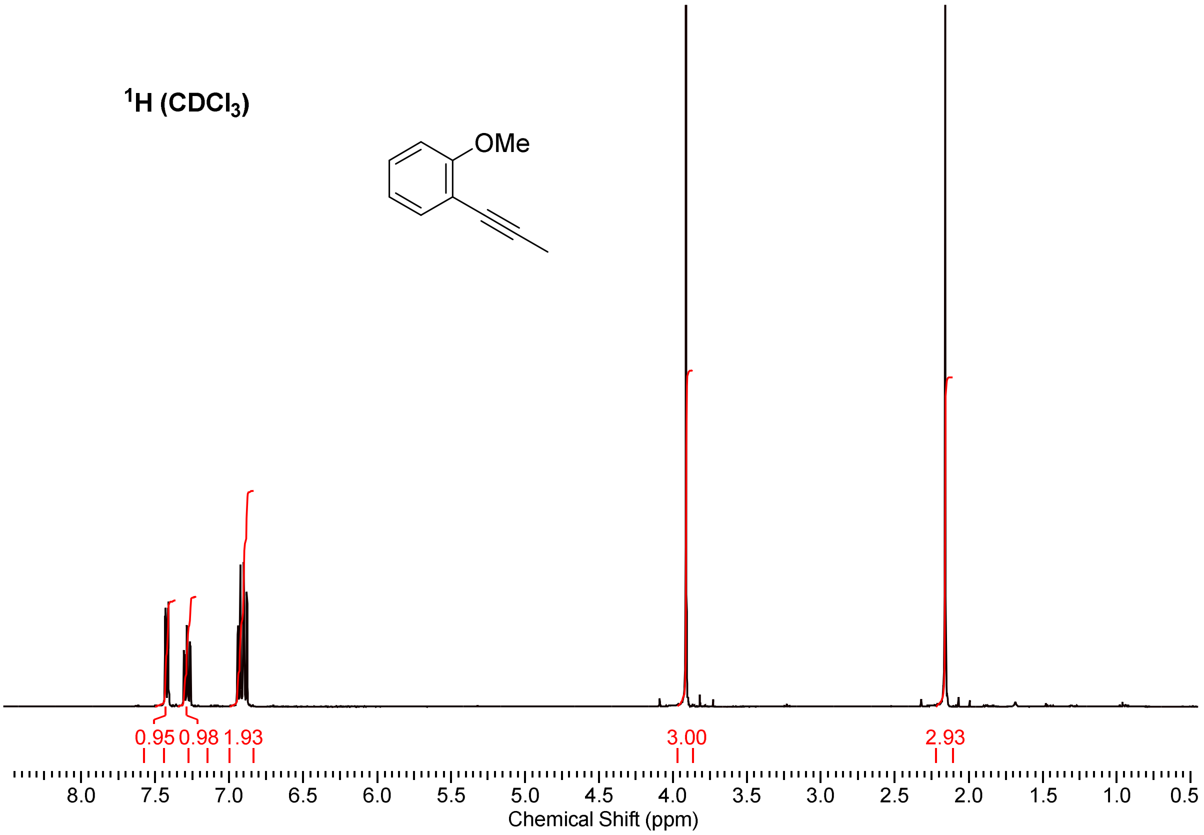

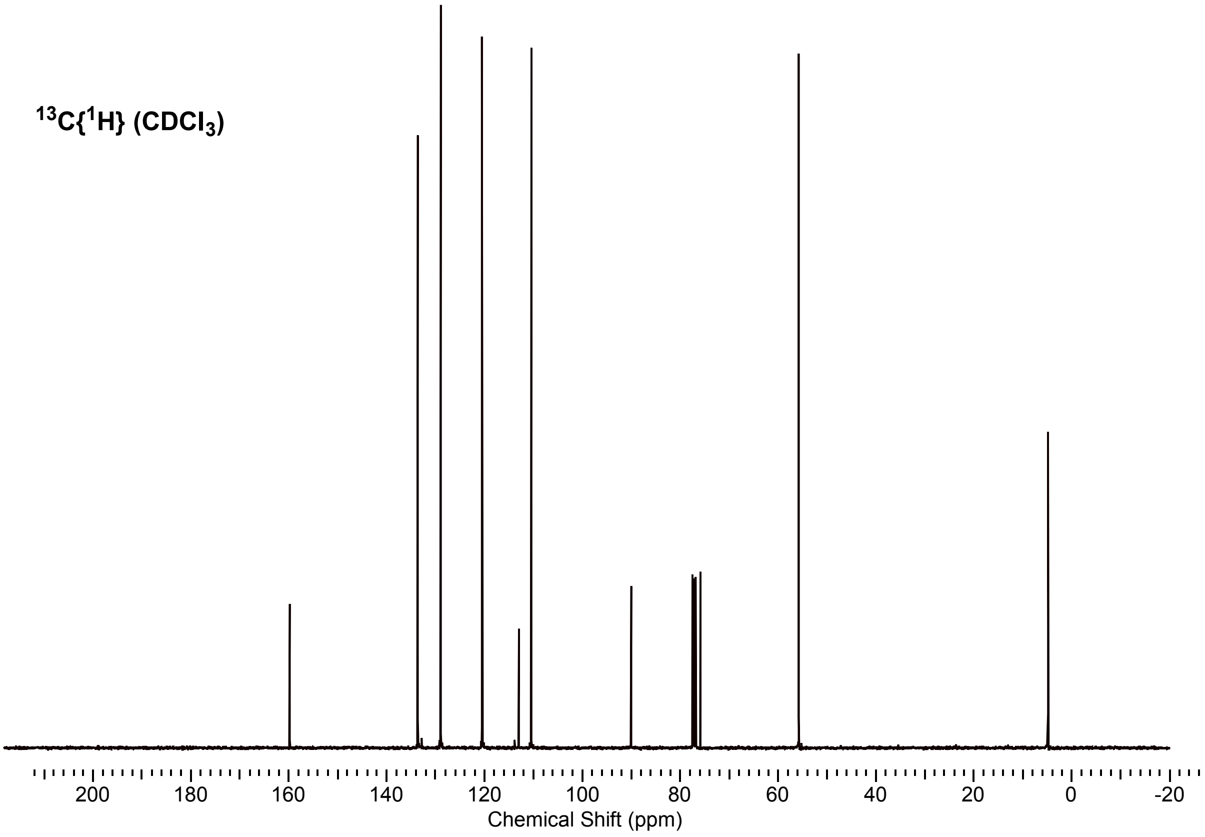

Ethyl 4-((2-methoxyphenyl)ethynyl)benzoate **(3h)**

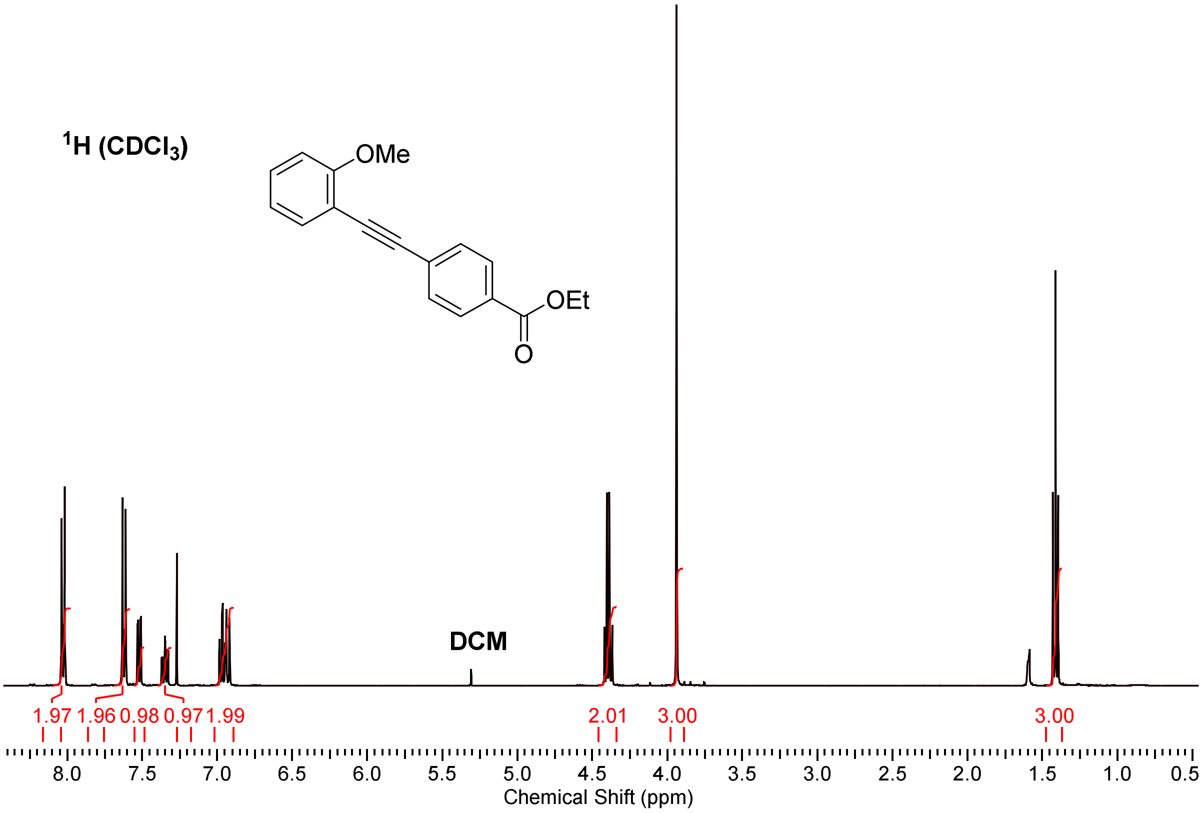

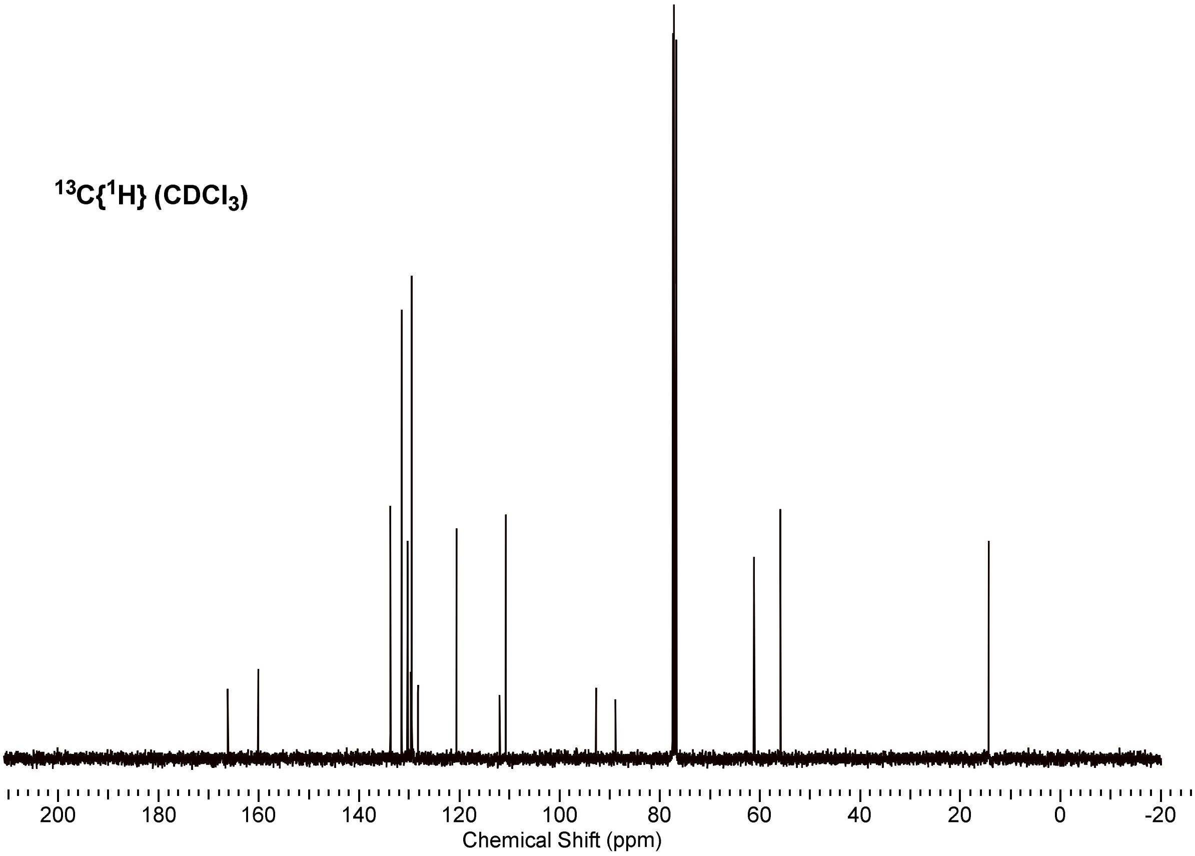

2-methoxy-4-nitro-1-(phenylethynyl)benzene **(3i)**

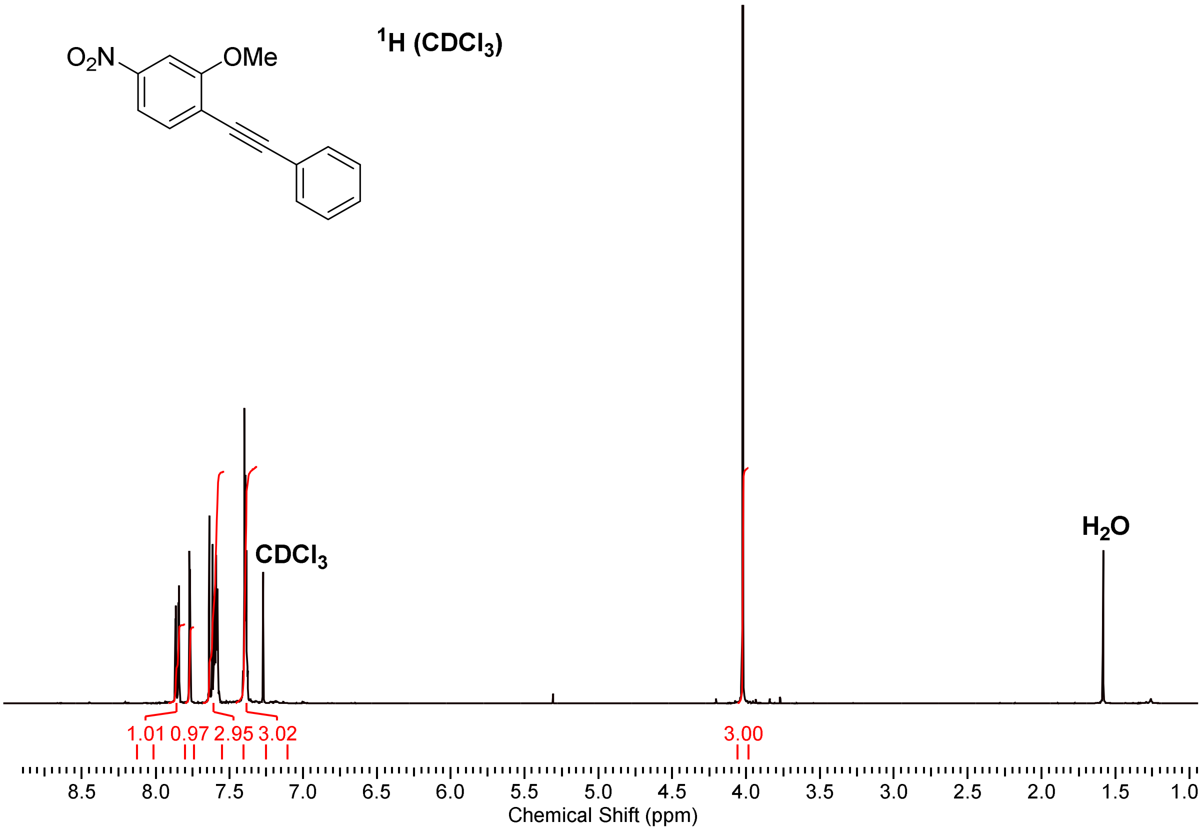

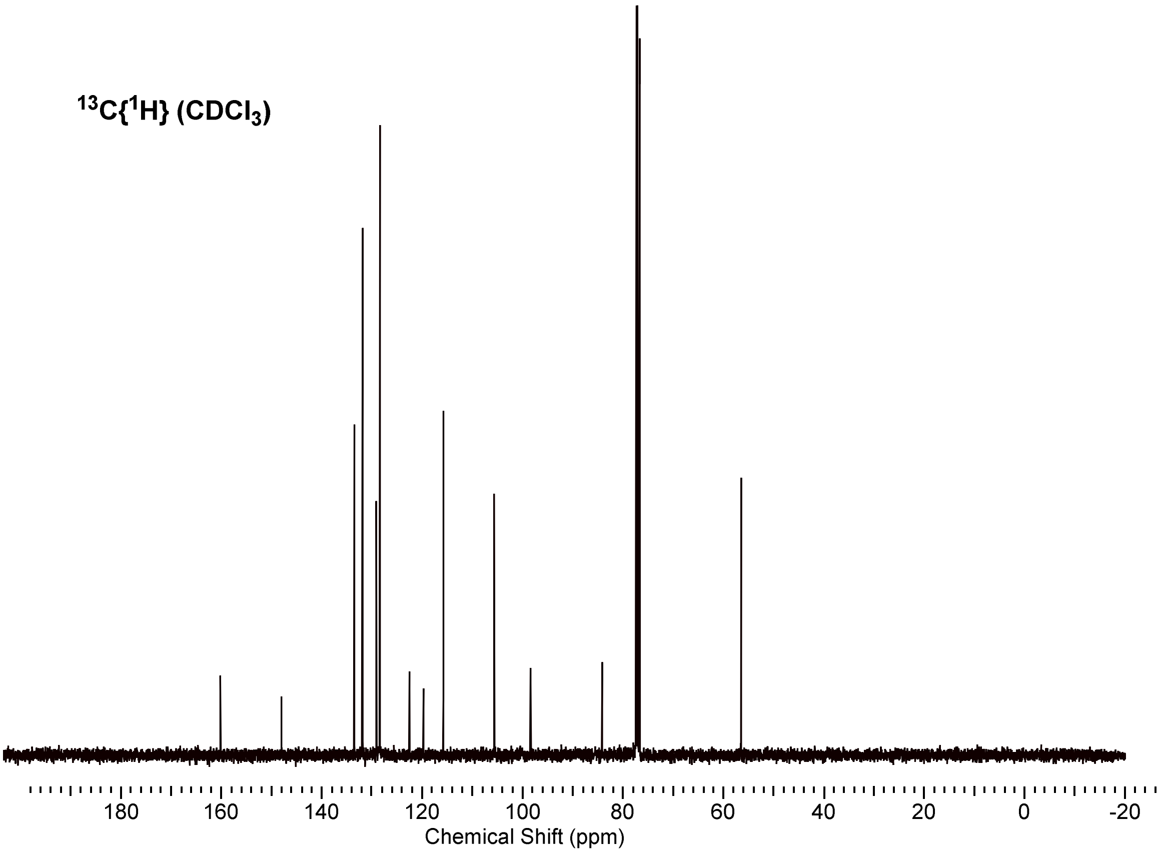

1-((2-methoxyphenyl)ethynyl)naphthalene **(3j)**

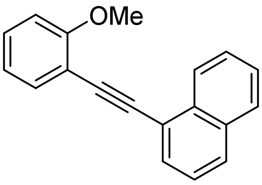

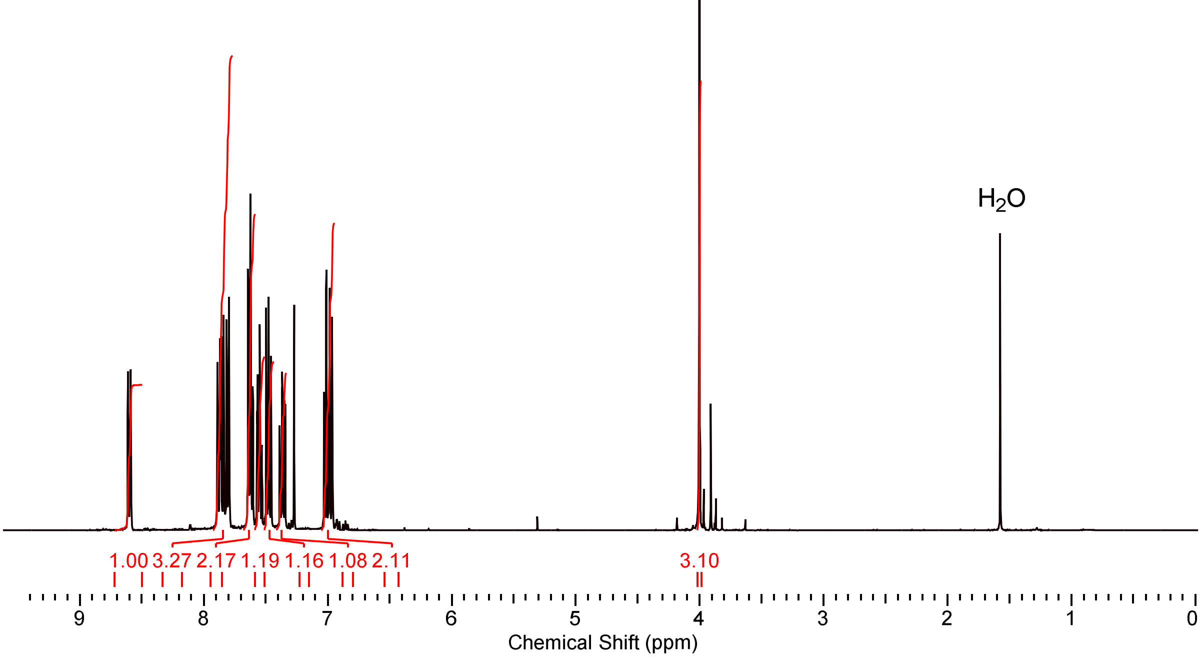

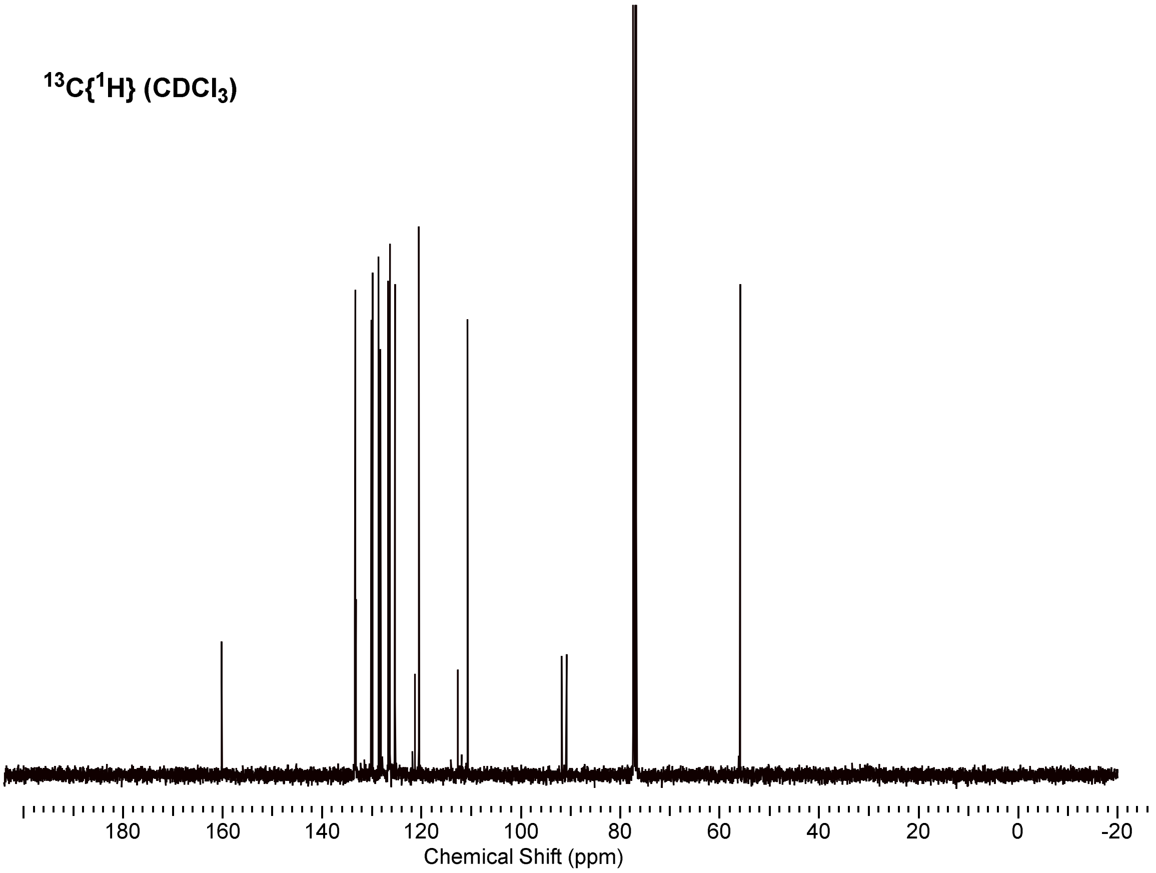

Methyl(2-(phenylethynyl)phenyl)sulfane **(9a)**

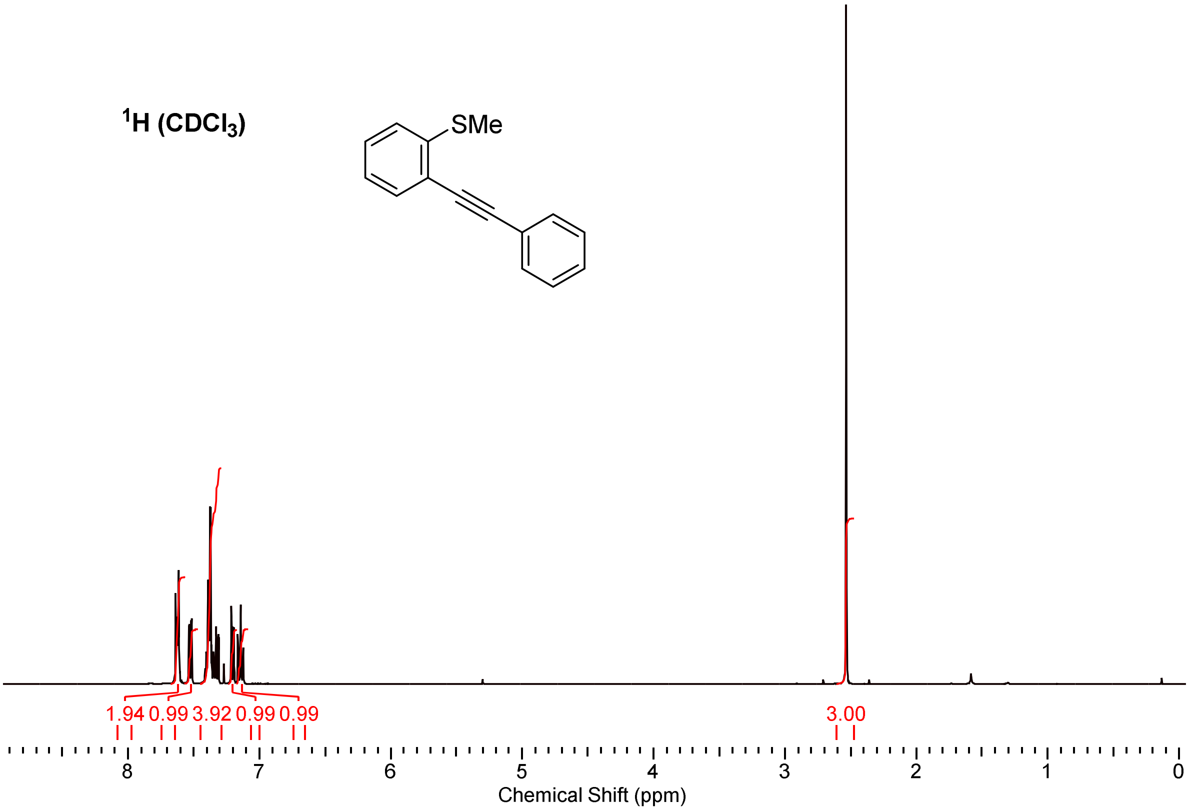

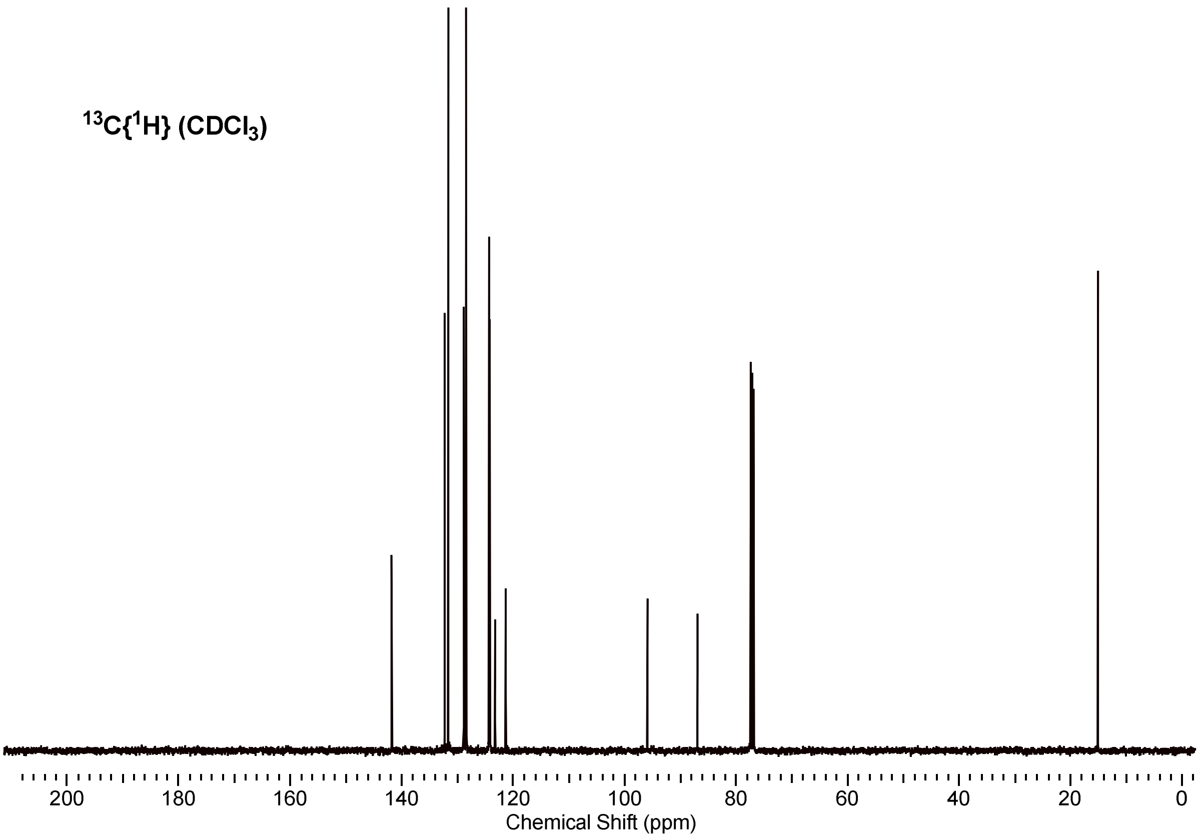

Methyl(2-(naphthalen-1-ylethynyl)phenyl)sulfane **(9b)**

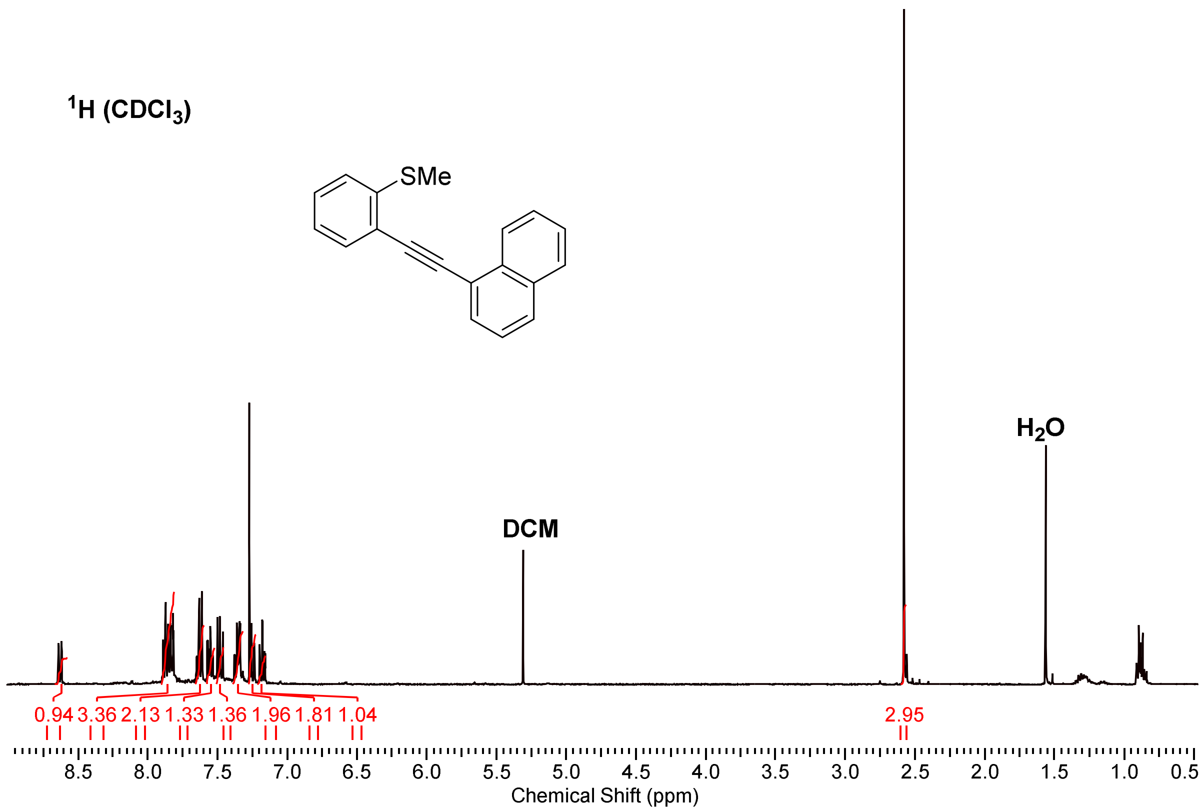

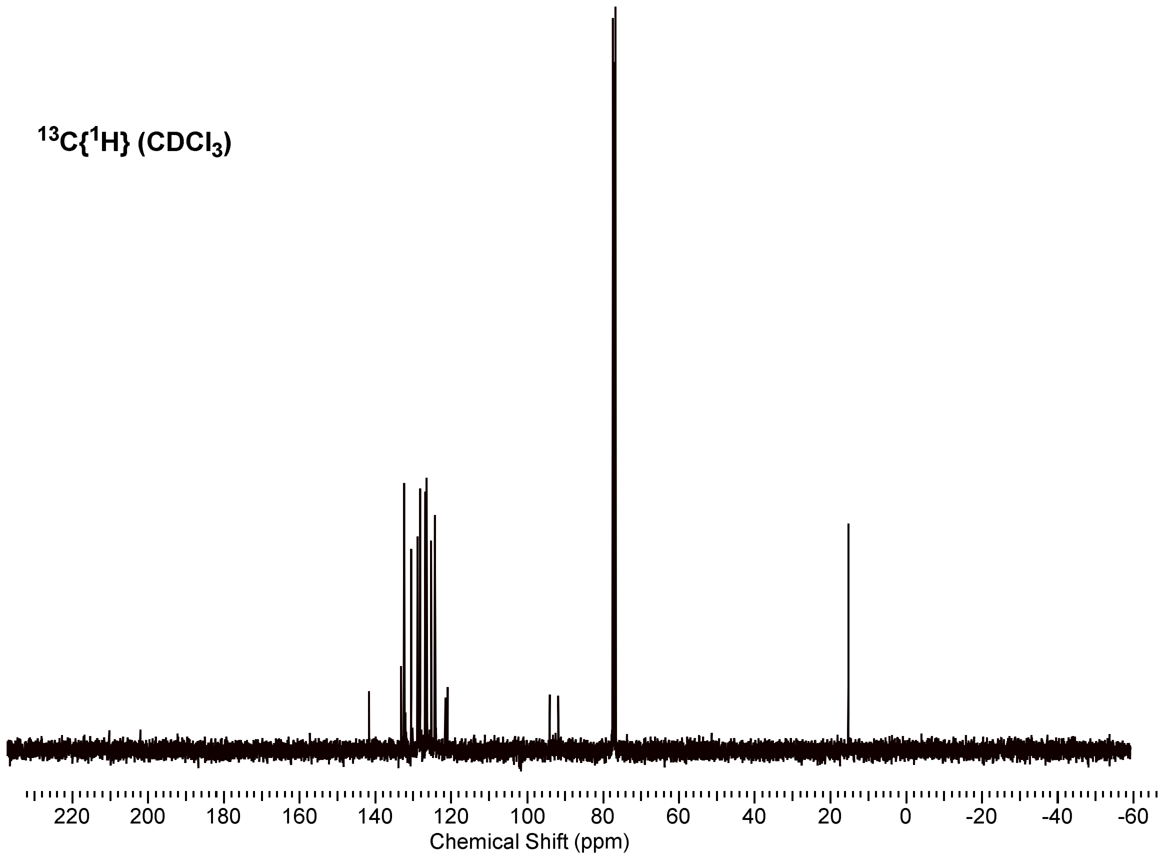

(2-(mesitylethynyl)phenyl)(methyl)sulfane **(9c)**

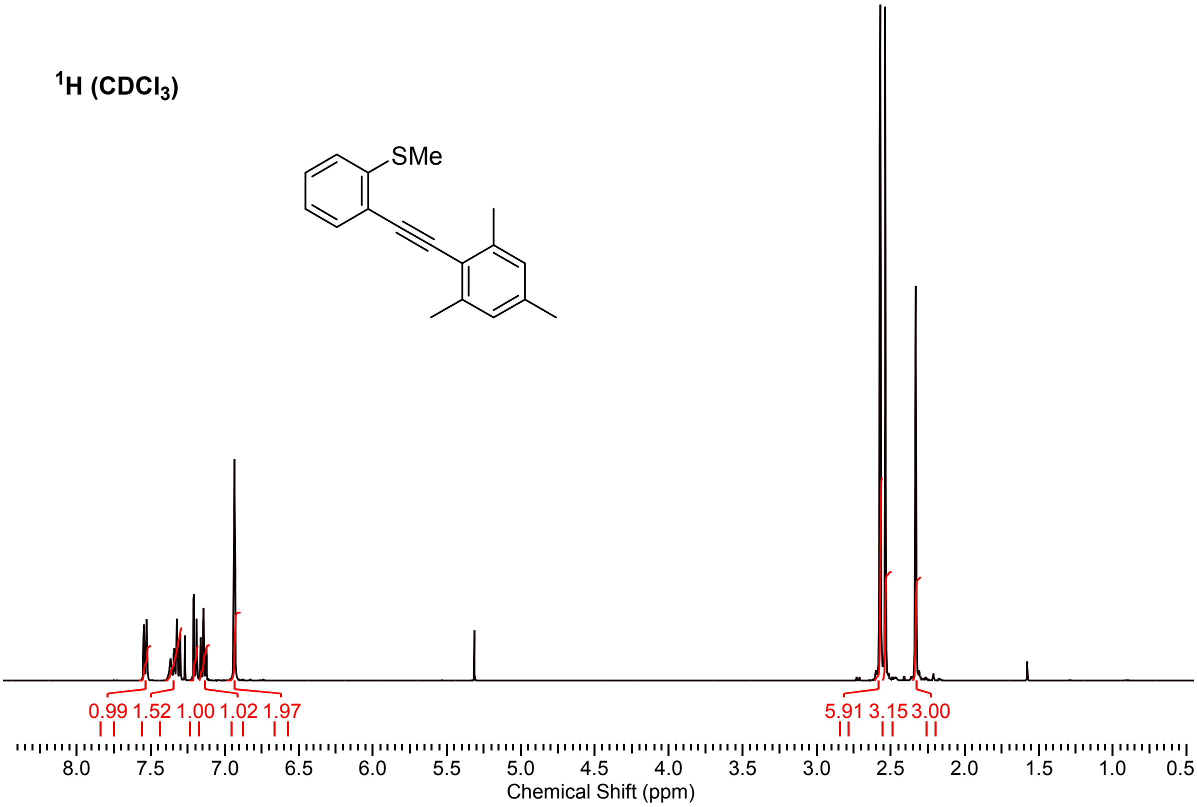

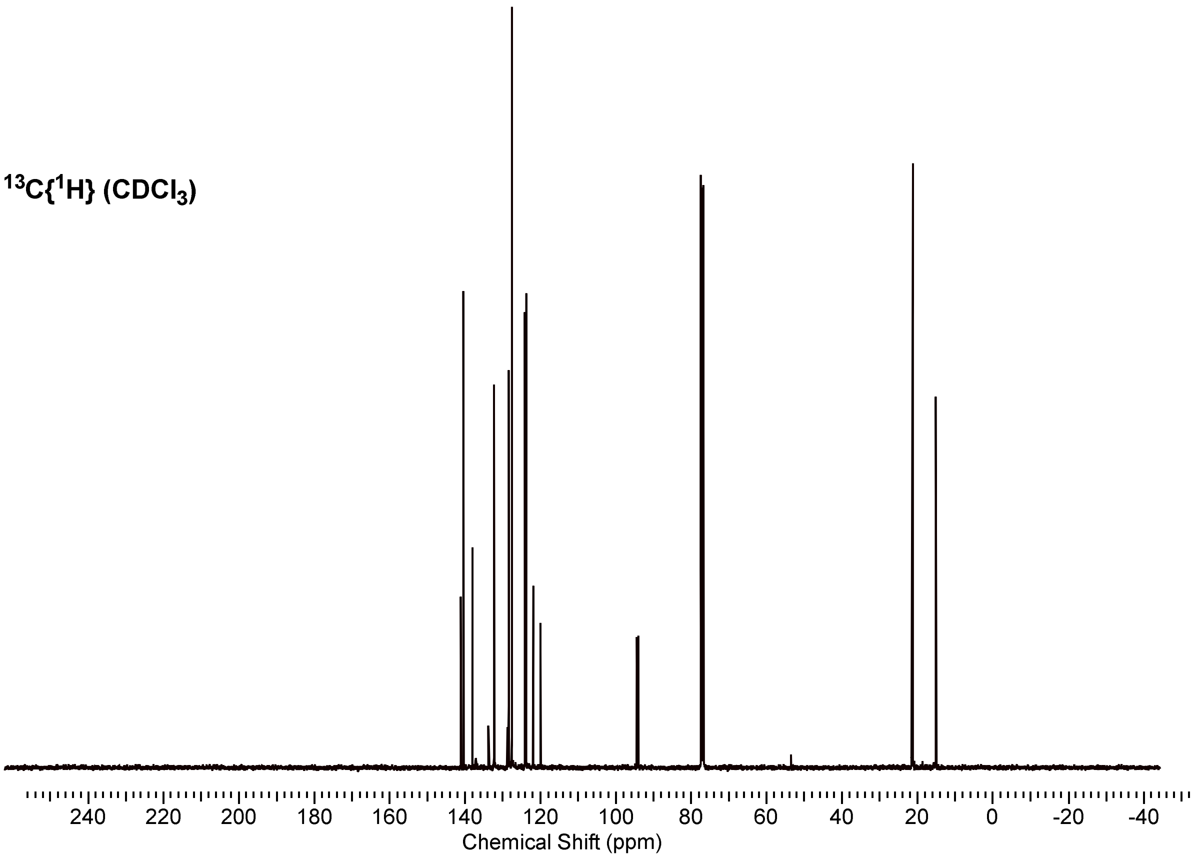

3-((2-(methylthio)phenyl)ethynyl)thiophene **(9d)**

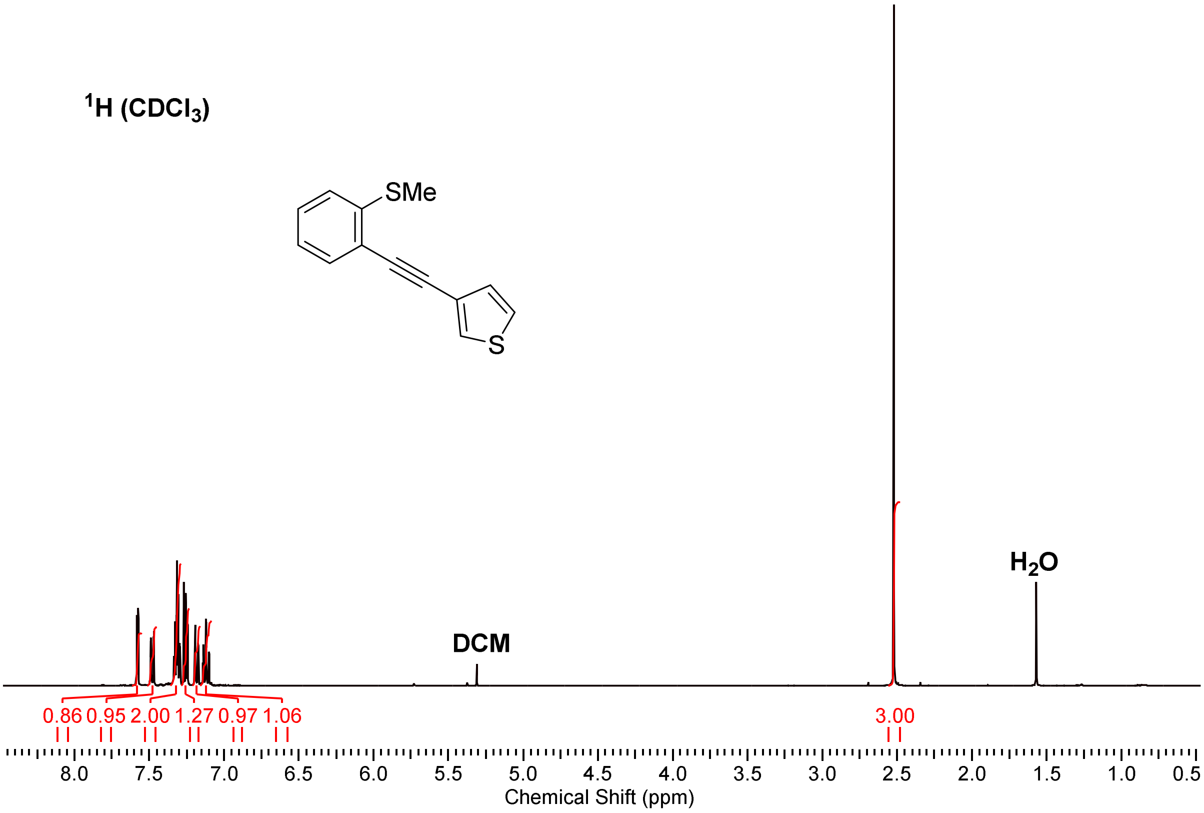

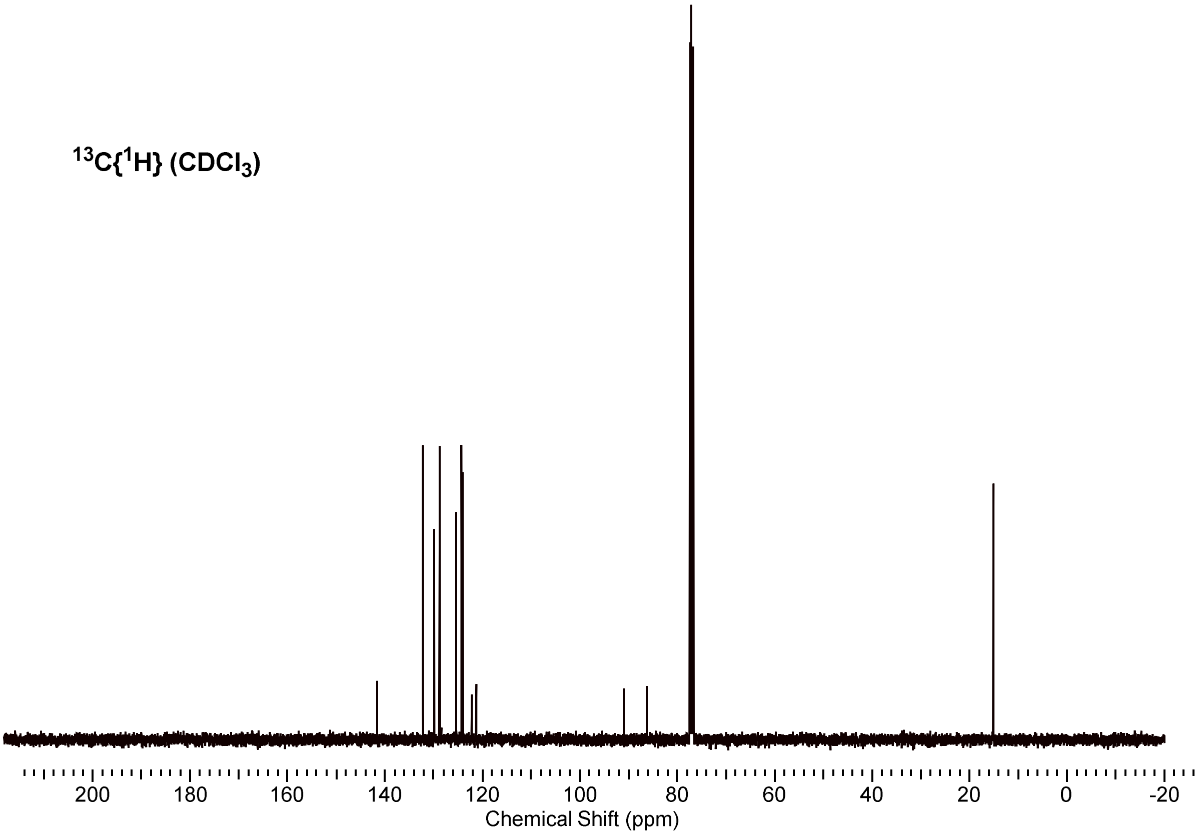

4,4,5,5-tetramethyl-2-(2-phenylbenzofuran-3-yl)-1,3,2-dioxaborolane **(4a)**

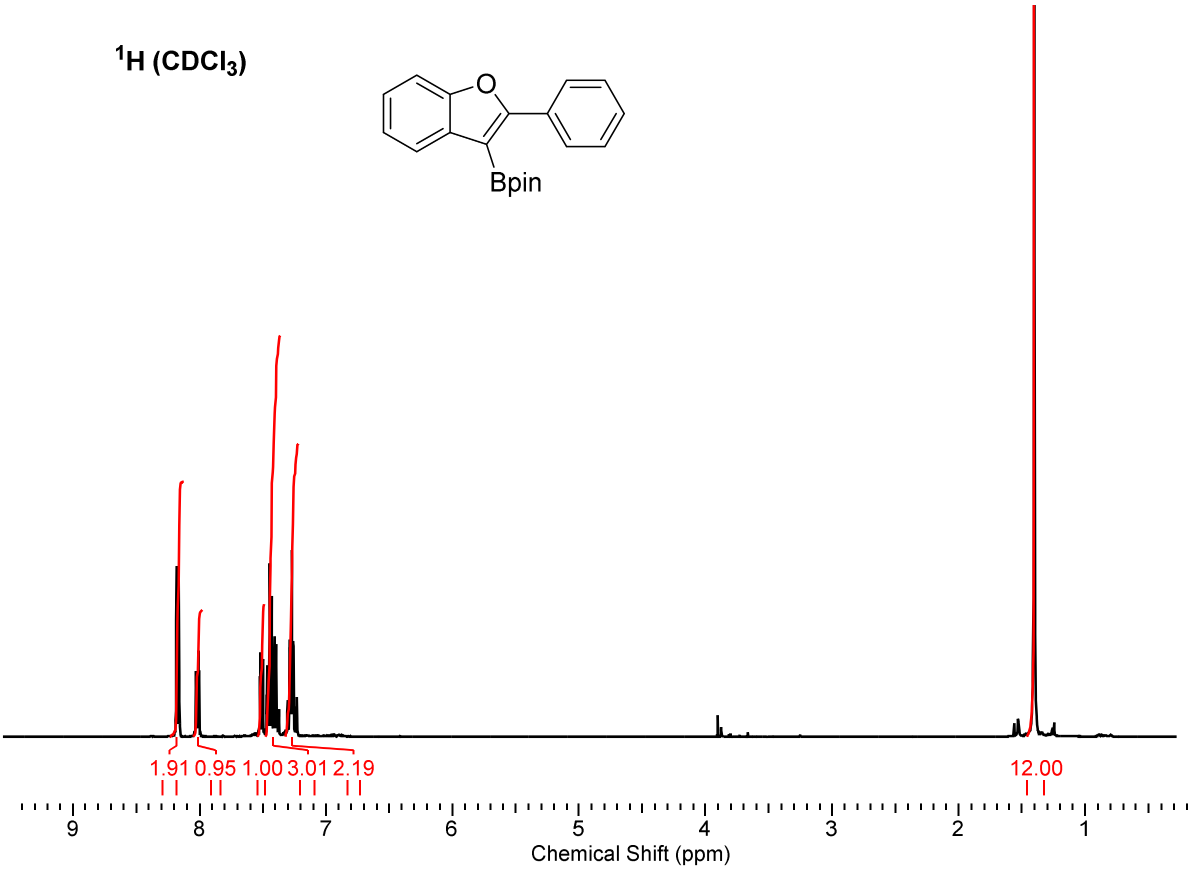

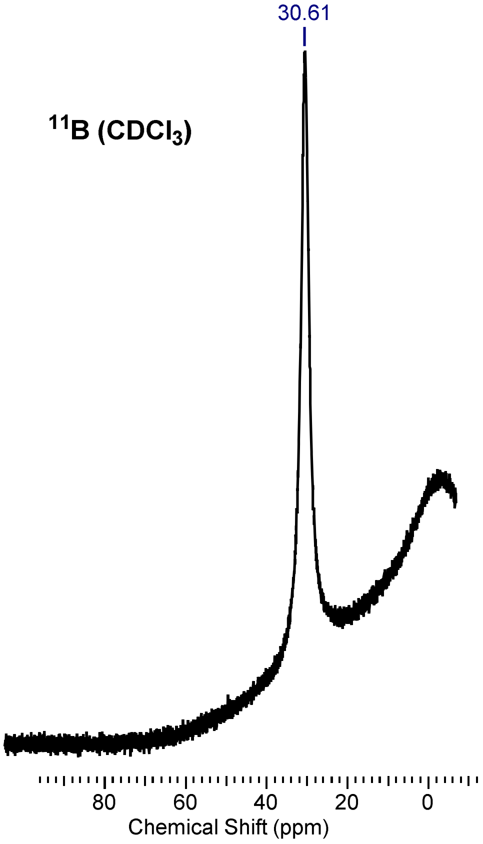

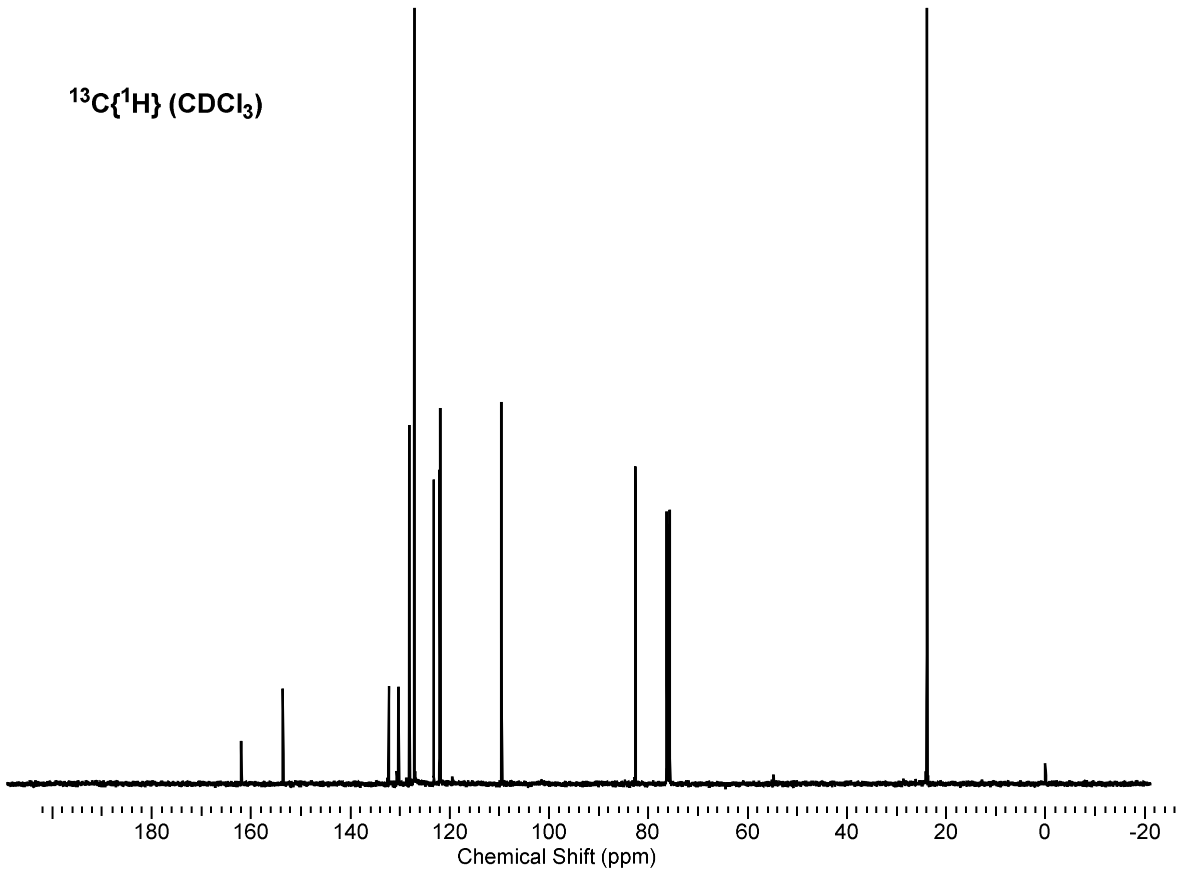
$^{13}C\{^1H\}$ (CDCl$_3$)
180 160 140 120 100 80 60 40 20 0 -20
Chemical Shift (ppm)

4,4,5,5-tetramethyl-2-(5-methyl-2-phenylbenzofuran-3-yl)-1,3,2-dioxaborolane **(4b)**

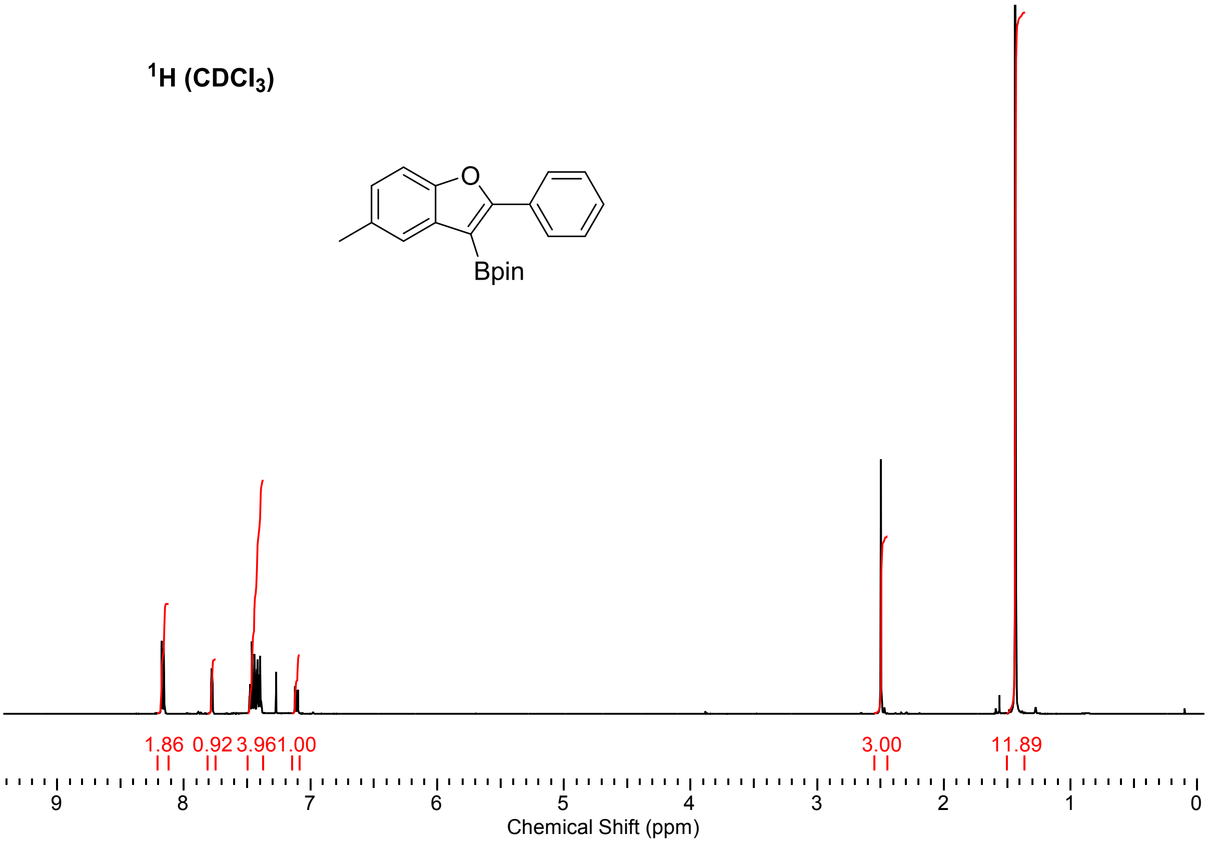

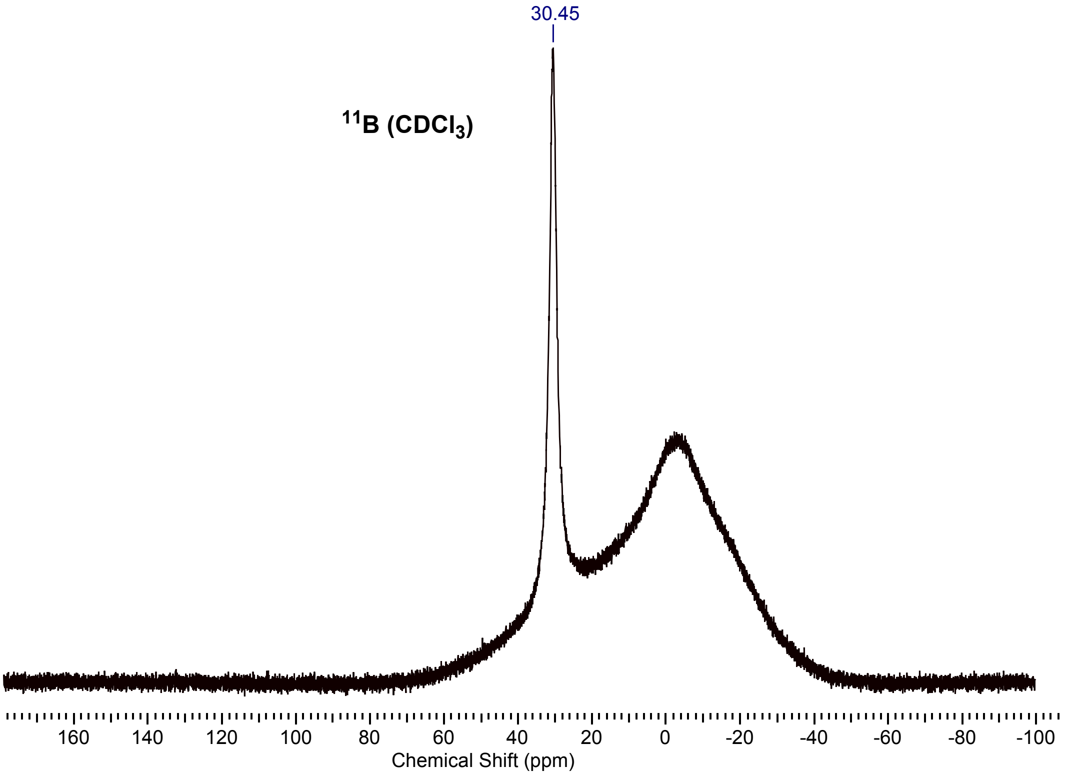

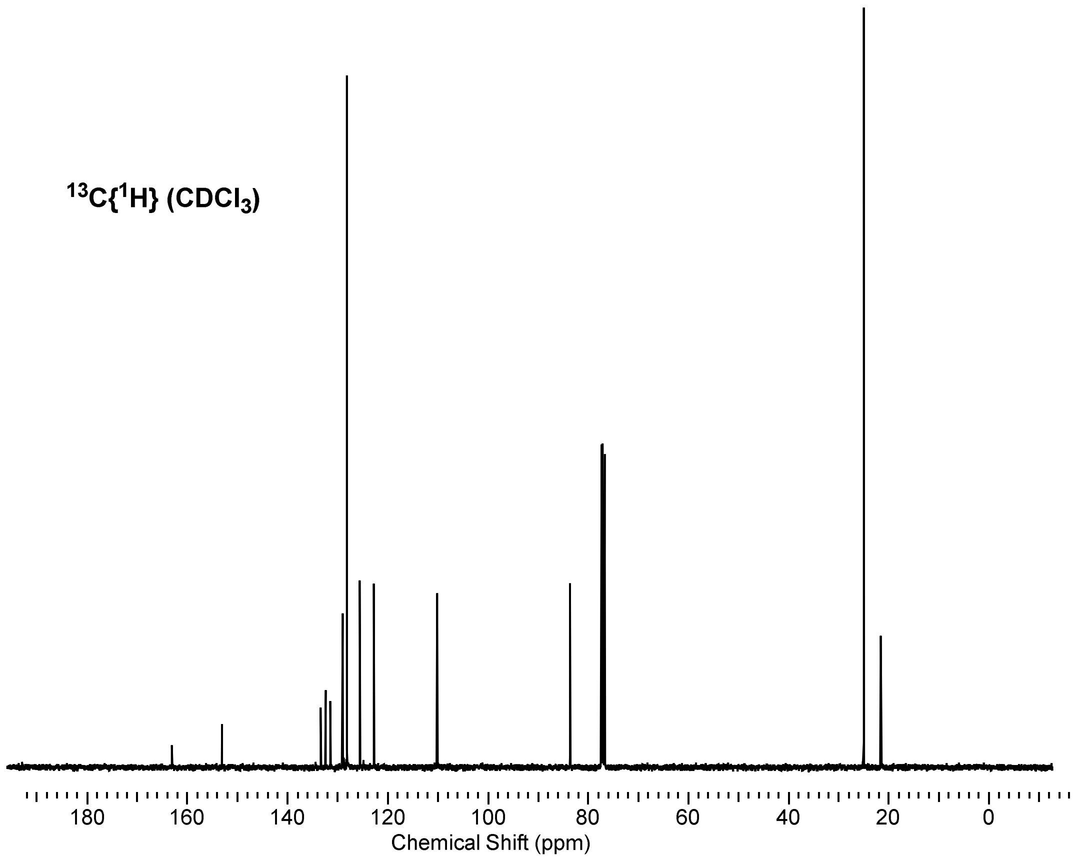
$^{13}C\{^1H\}$ ($CDCl_3$)
180
160
140
120
100
80
60
40
20
0
Chemical Shift (ppm)

2-(5-chloro-2-phenylbenzofuran-3-yl)-4,4,5,5-tetramethyl-1,3,2-dioxaborolane **(4c)**

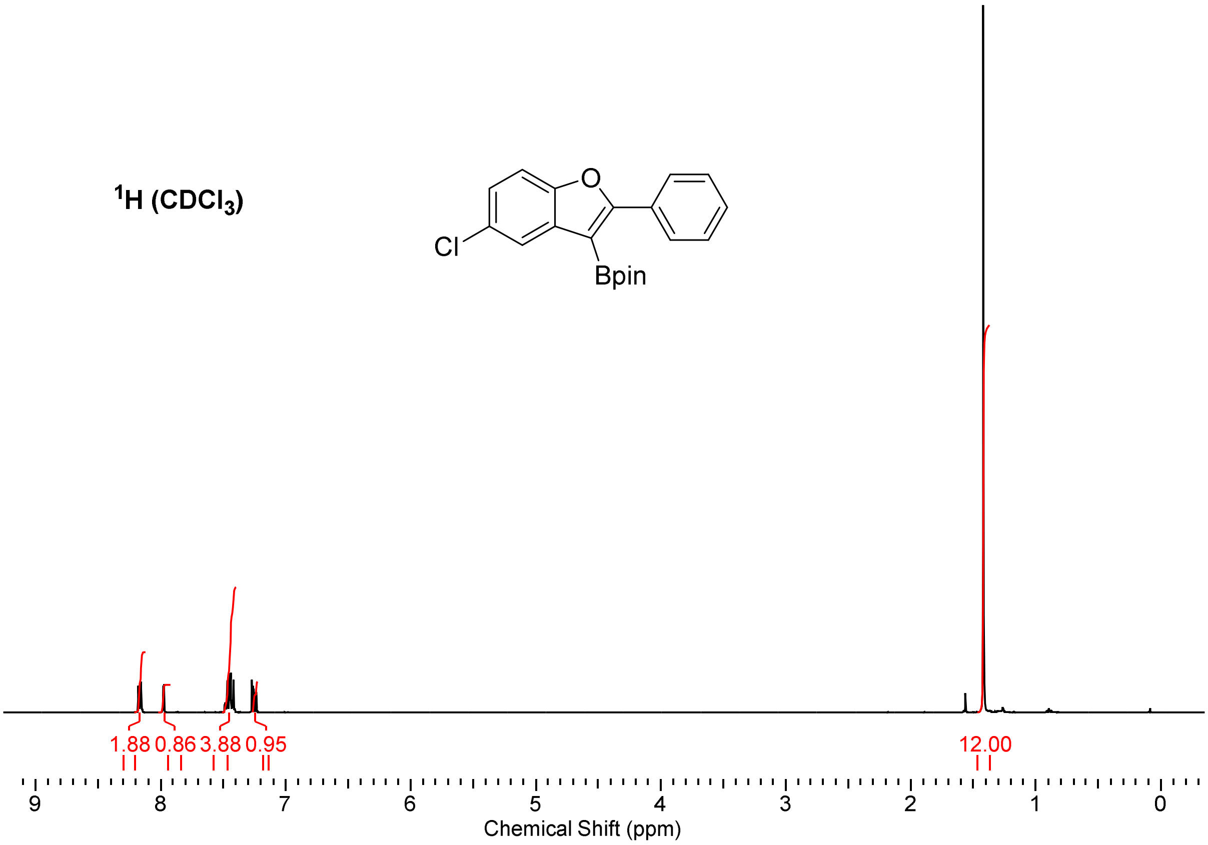

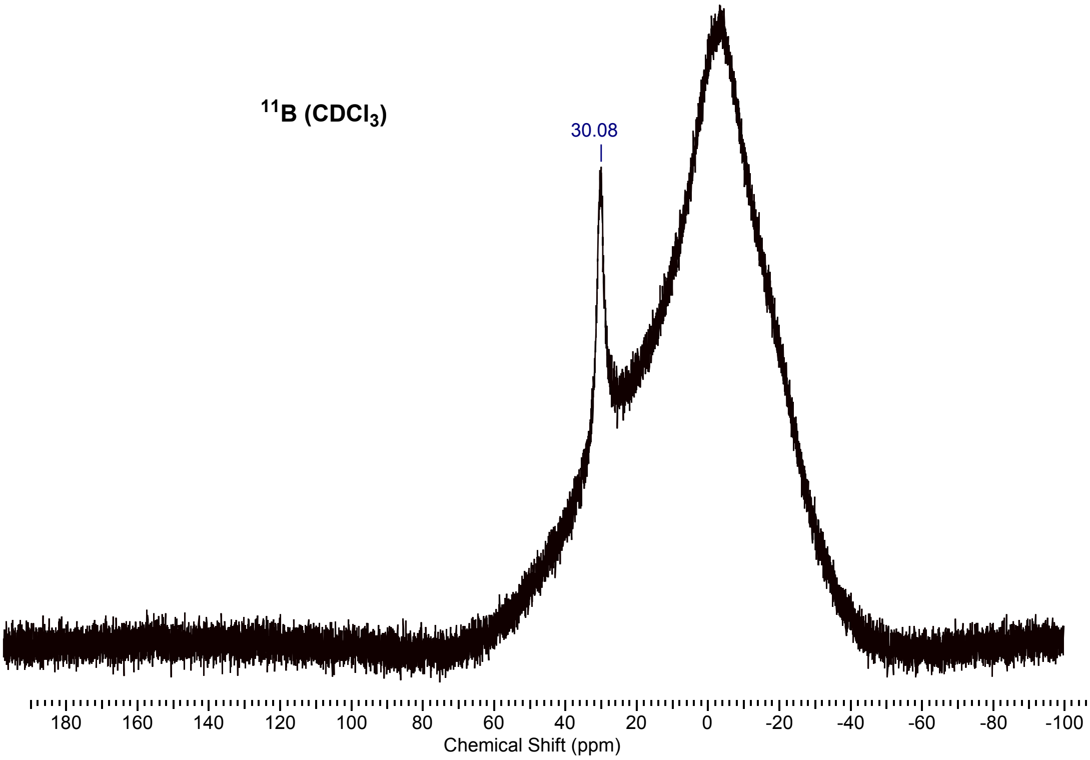

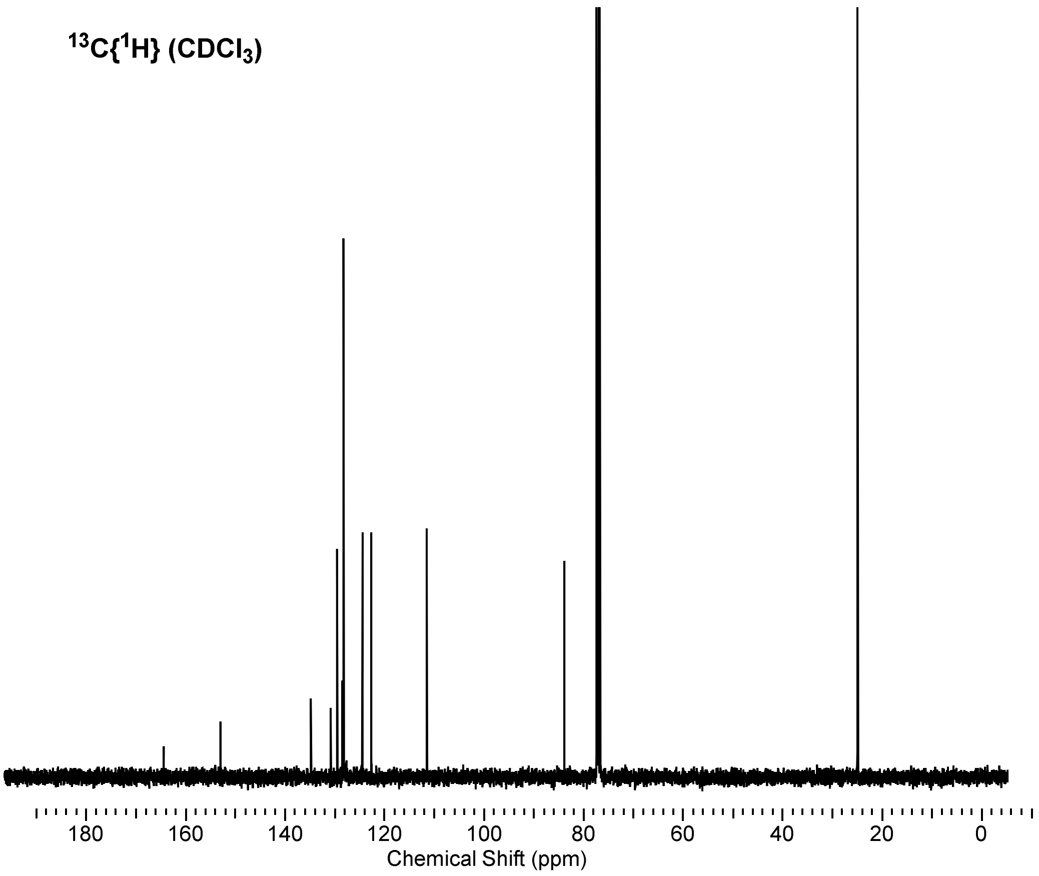

$^{13}C\{^1H\}$ ($CDCl_3$)
180
160
140
120
100
80
60
40
20
0
Chemical Shift (ppm)

2-(7-fluoro-2-phenylbenzofuran-3-yl)-4,4,5,5-tetramethyl-1,3,2-dioxaborolane **(4d)**

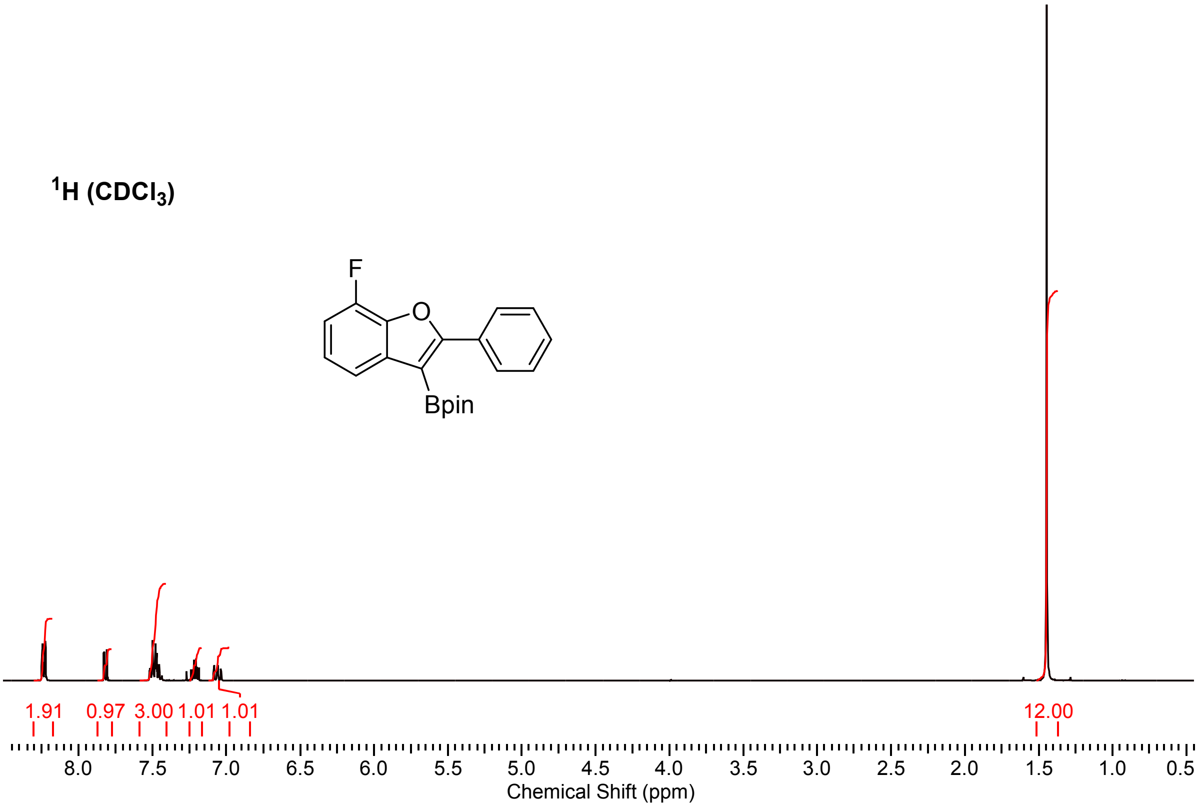

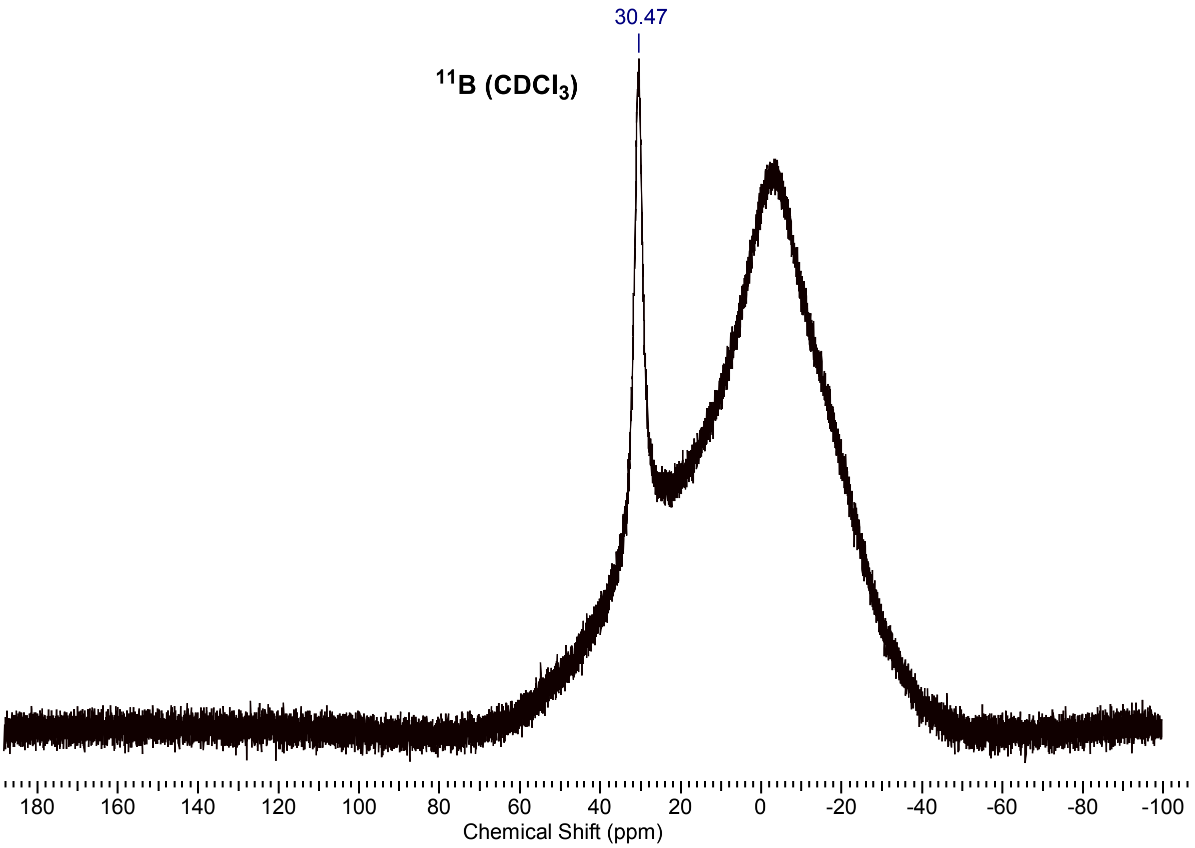

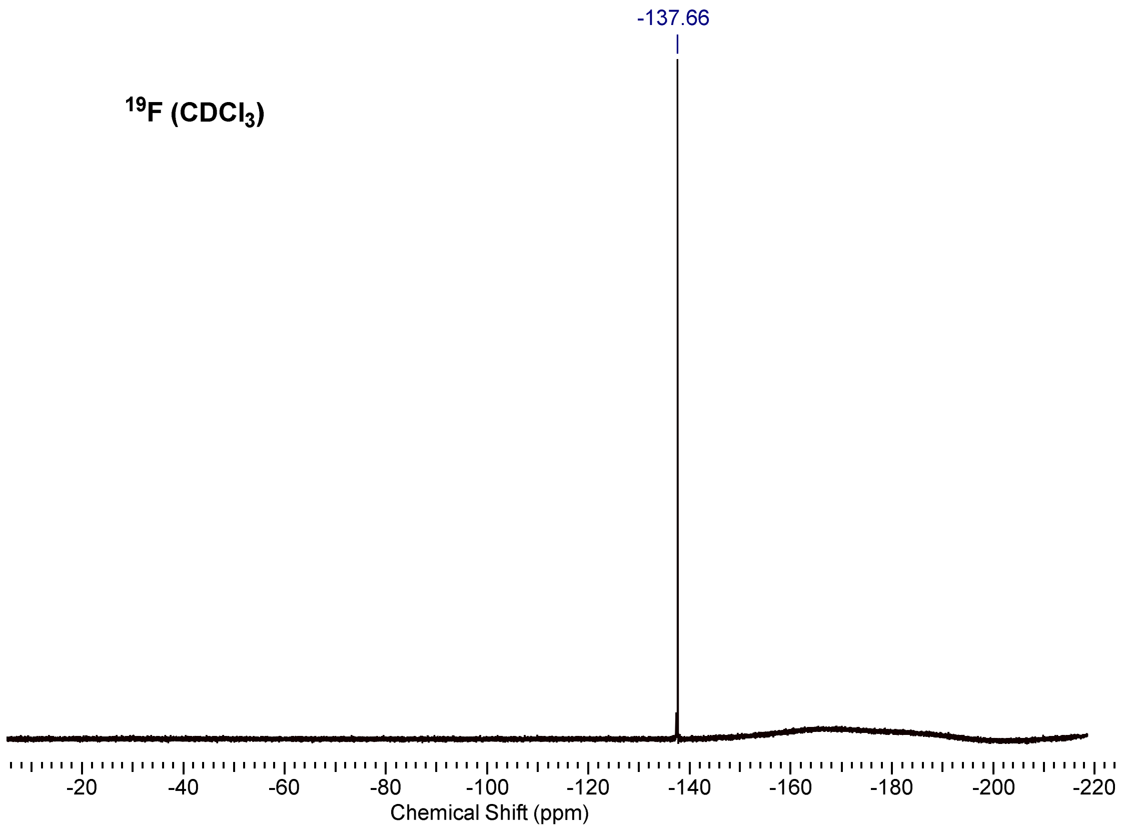
-137.66
19F (CDCl3)
-20
-40
-60
-80
-100
-120
-140
-160
-180
-200
-220
Chemical Shift (ppm)

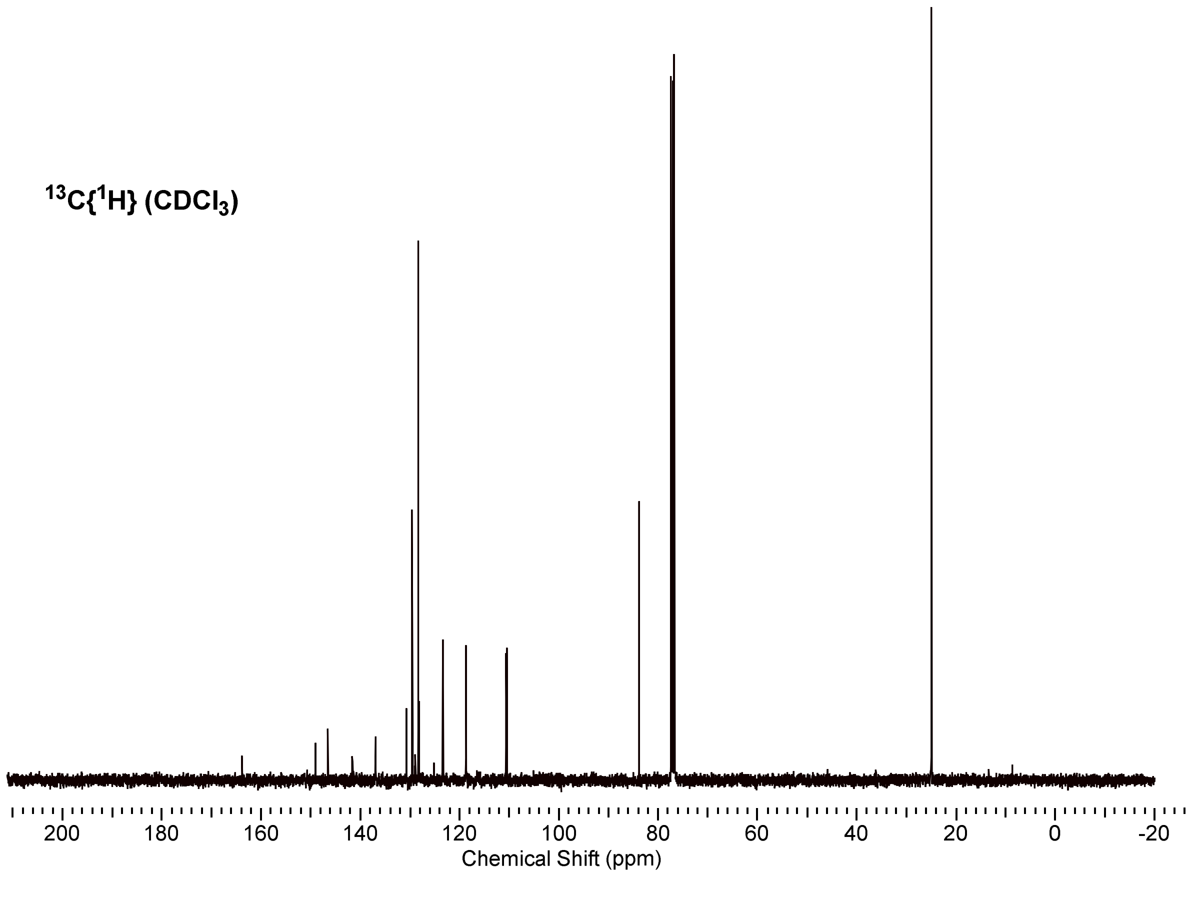
13C{1H} (CDCl3)
200
180
160
140
120
100
80
60
40
20
0
-20
Chemical Shift (ppm)

4,4,5,5-tetramethyl-2-(4-methyl-2-phenylbenzofuran-3-yl)-1,3,2-dioxaborolane **(4e)**

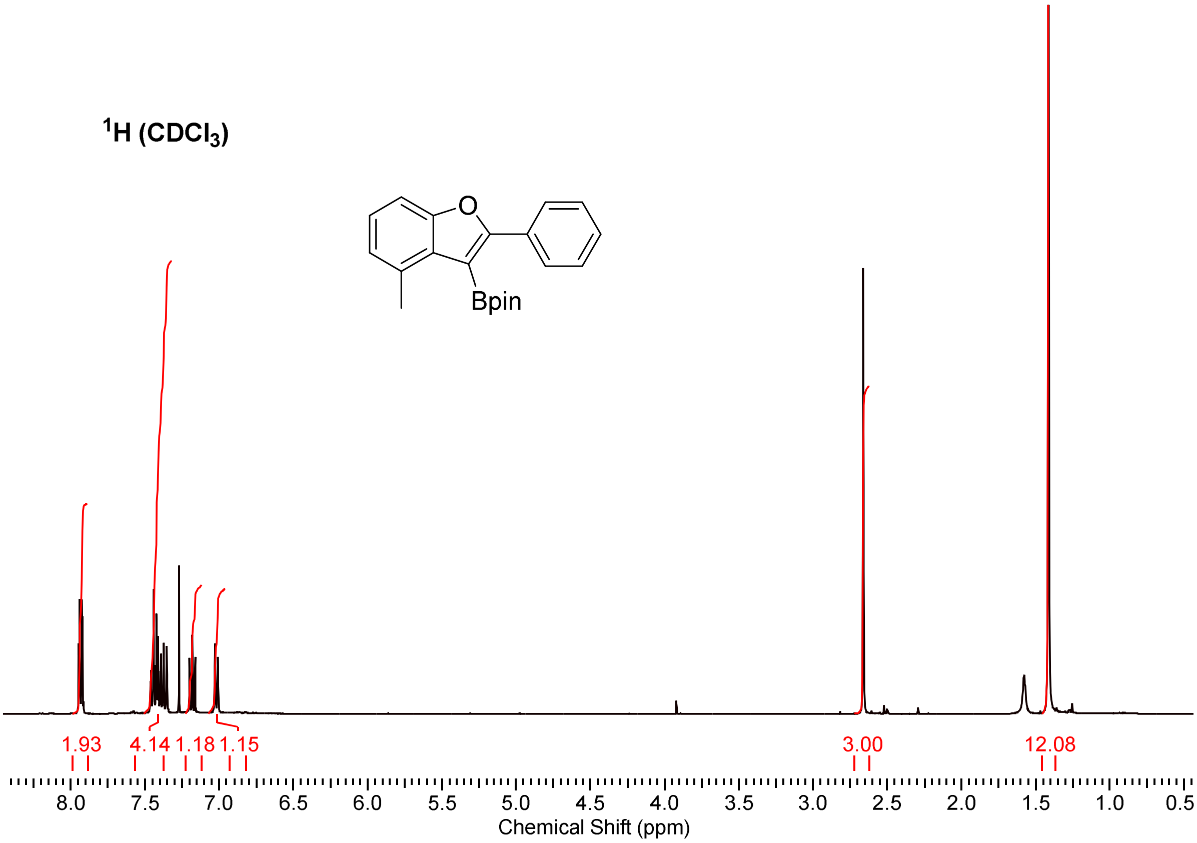

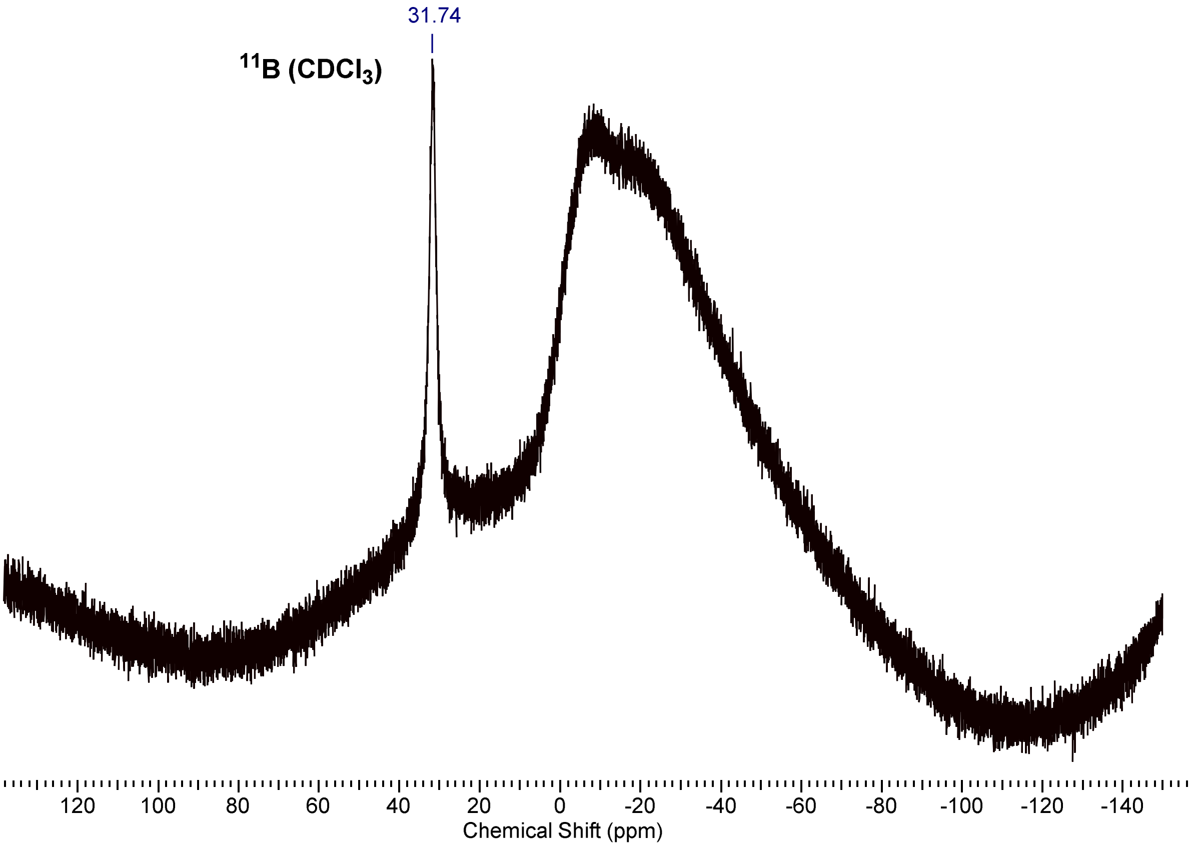

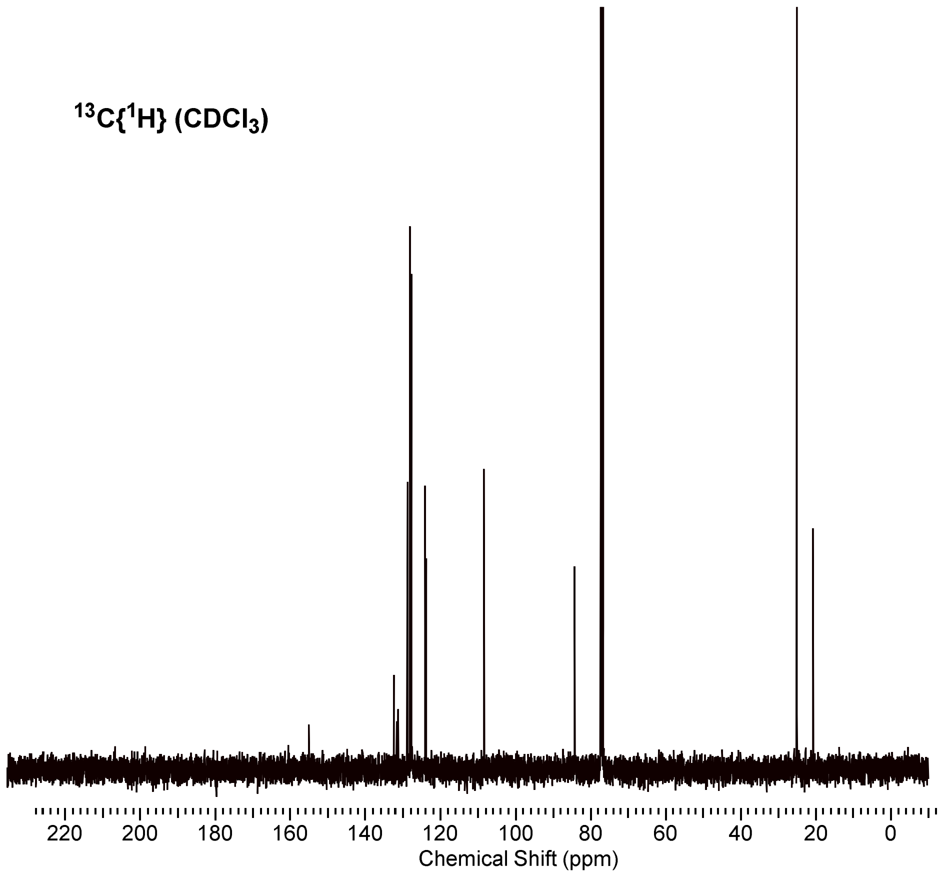

$^{13}C\{^1H\}$ (CDCl$_3$)
220 200 180 160 140 120 100 80 60 40 20 0
Chemical Shift (ppm)

2-(2-benzylbenzofuran-3-yl)-4,4,5,5-tetramethyl-1,3,2-dioxaborolane **(4f)**

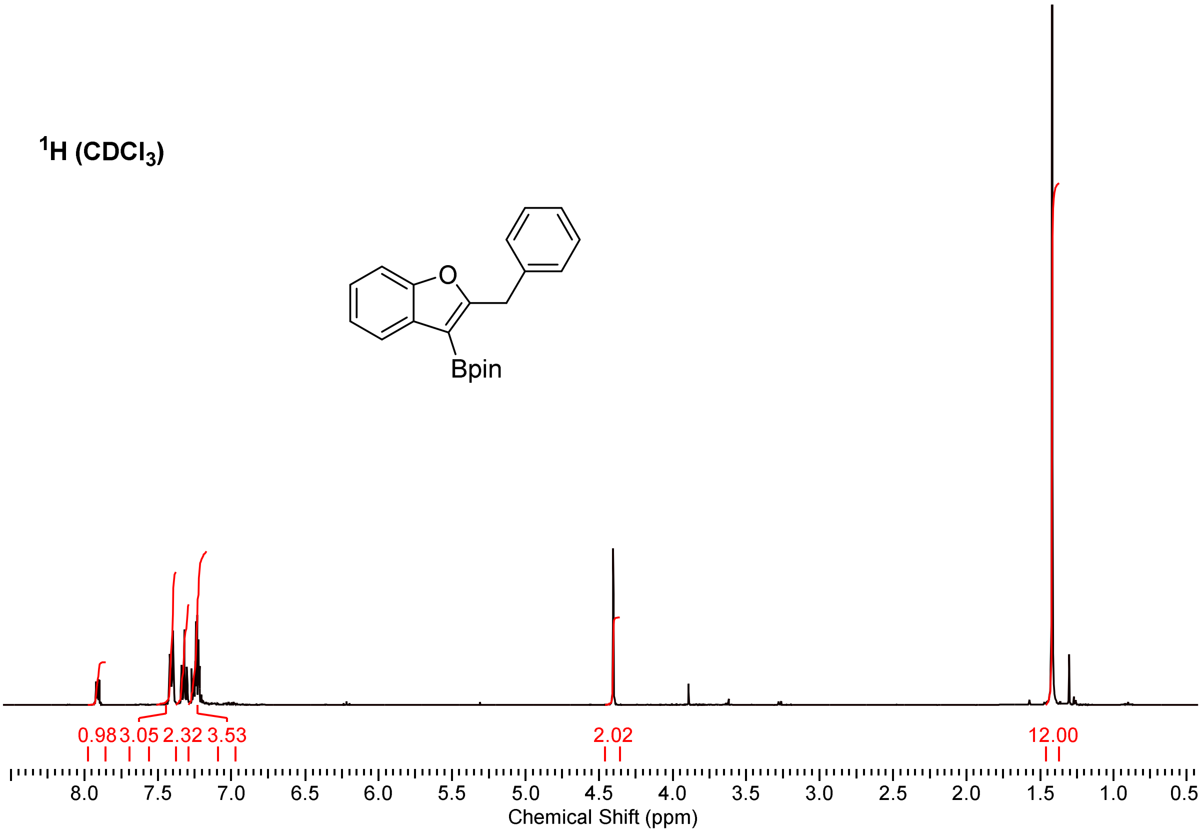

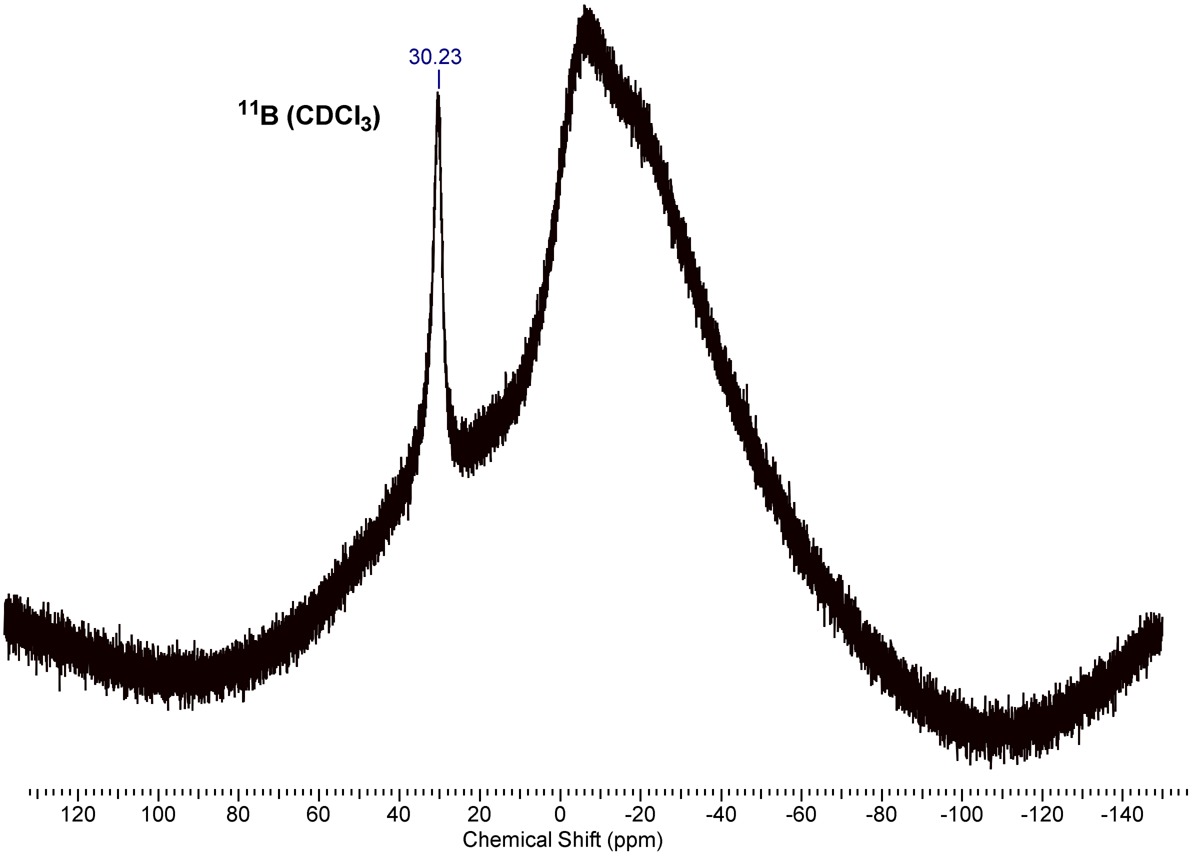

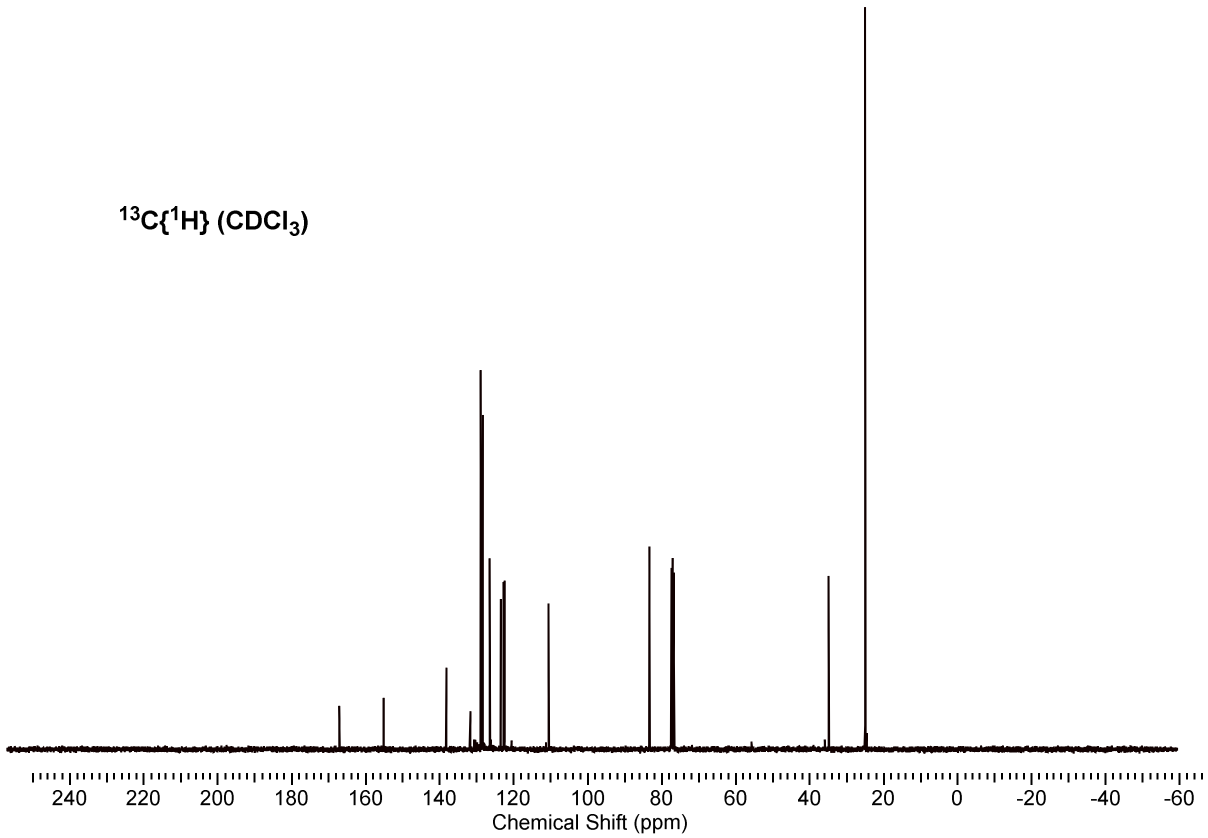

$^{13}C\{^1H\}$ ($CDCl_3$)
240 220 200 180 160 140 120 100 80 60 40 20 0 -20 -40 -60
Chemical Shift (ppm)

4,4,5,5-tetramethyl-2-(2-methylbenzofuran-3-yl)-1,3,2-dioxaborolane **(4g)**

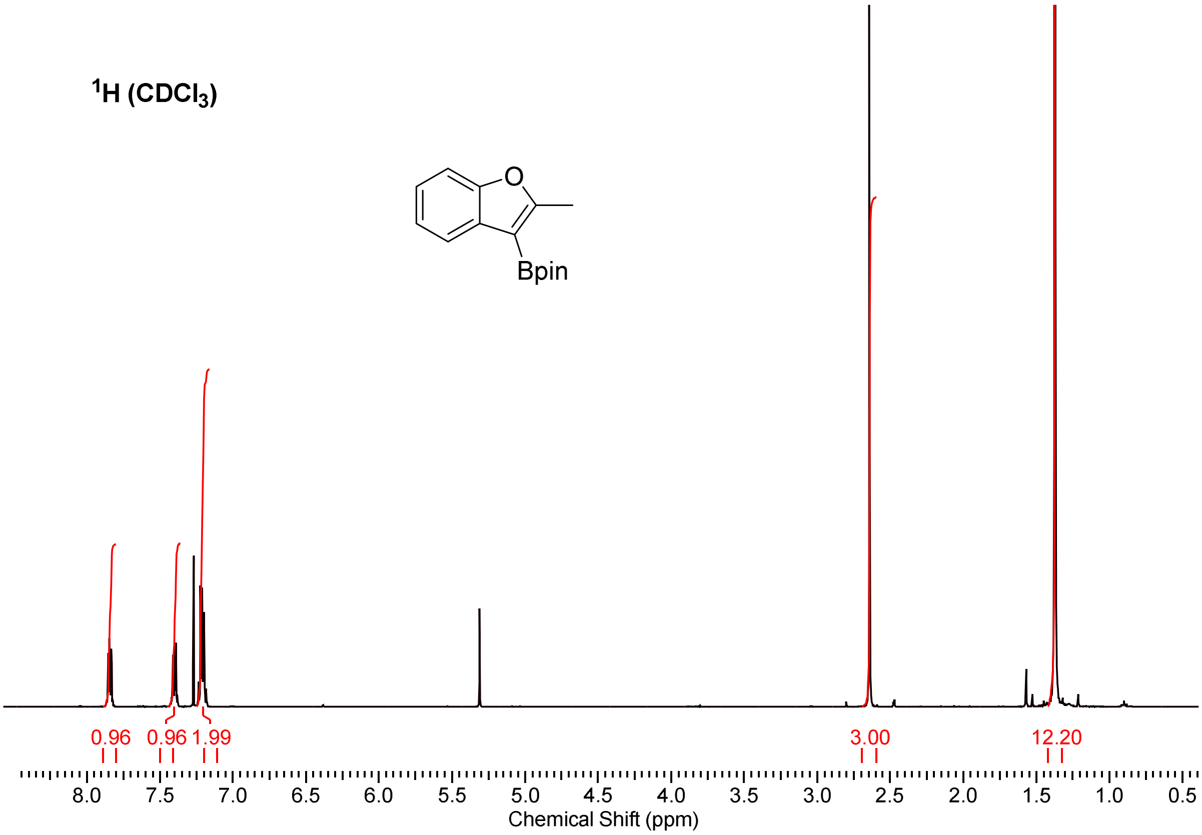

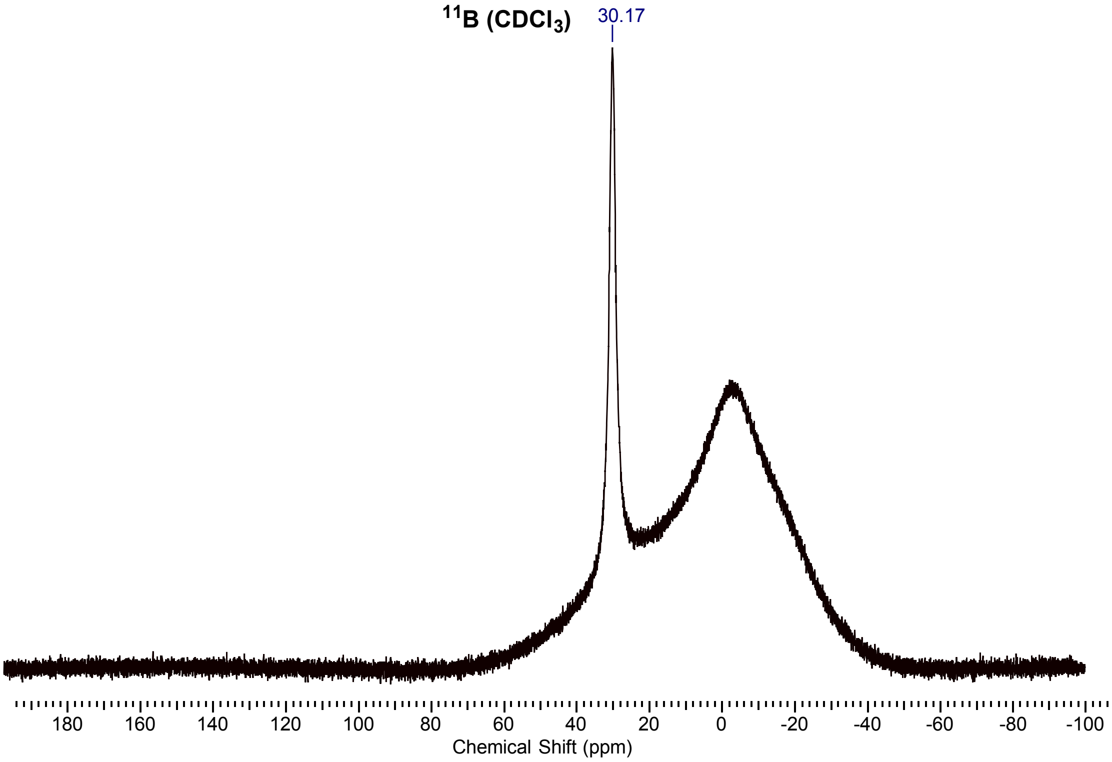

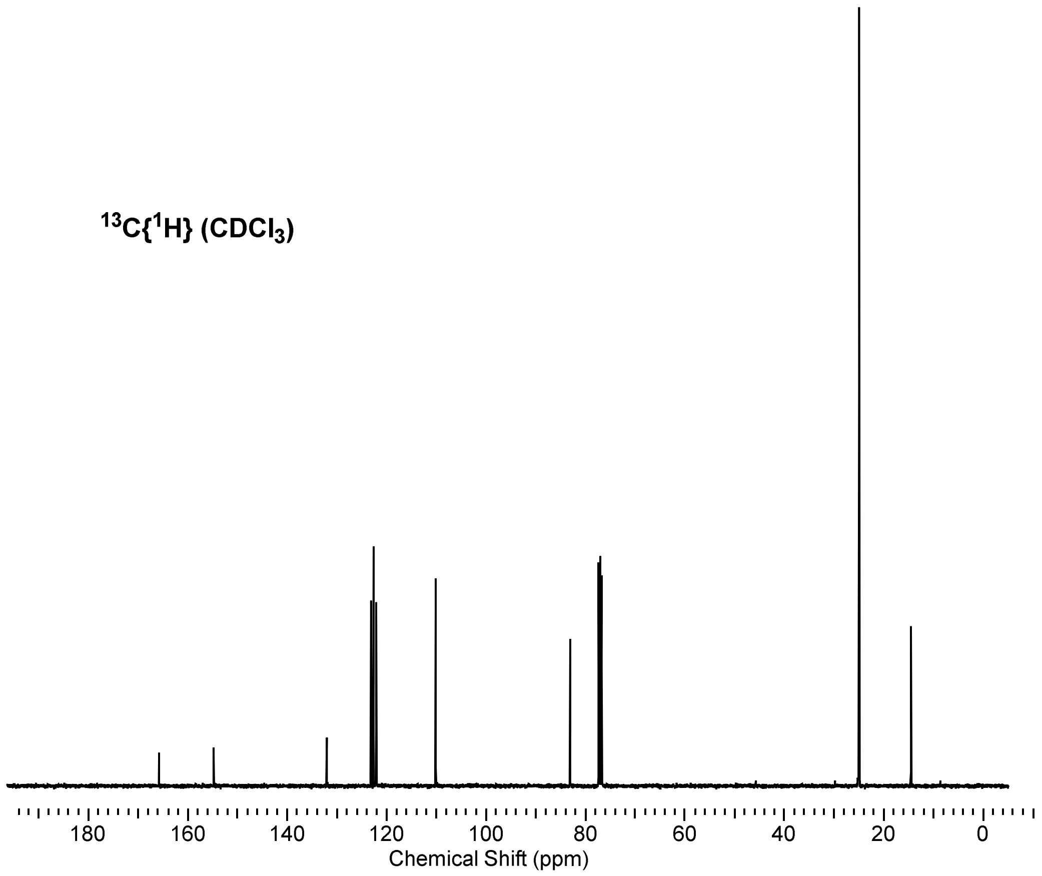
13C{1H} (CDCl3)
180
160
140
120
100
80
60
40
20
0
Chemical Shift (ppm)

Ethyl 4-(3-(4,4,5,5-tetramethyl-1,3,2-dioxaborolan-2-yl)benzofuran-2-yl)benzoate **(4h)**

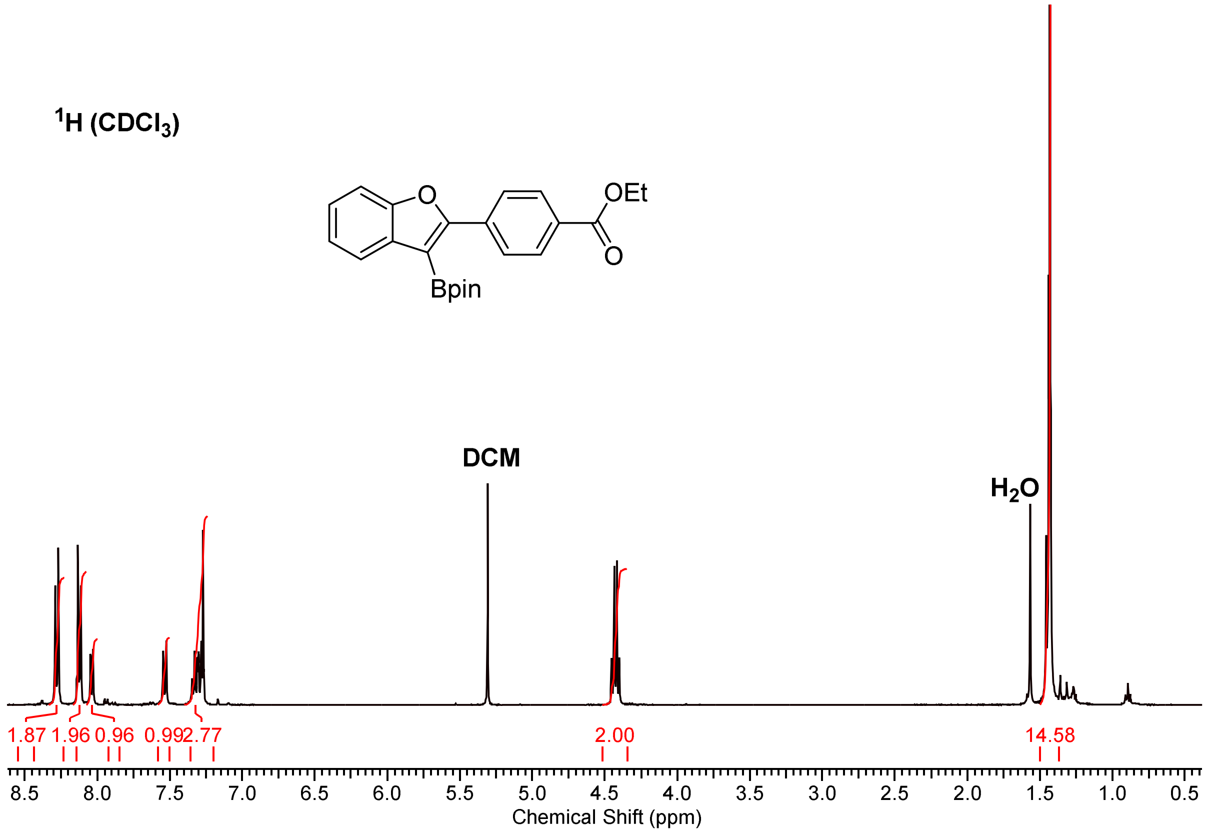

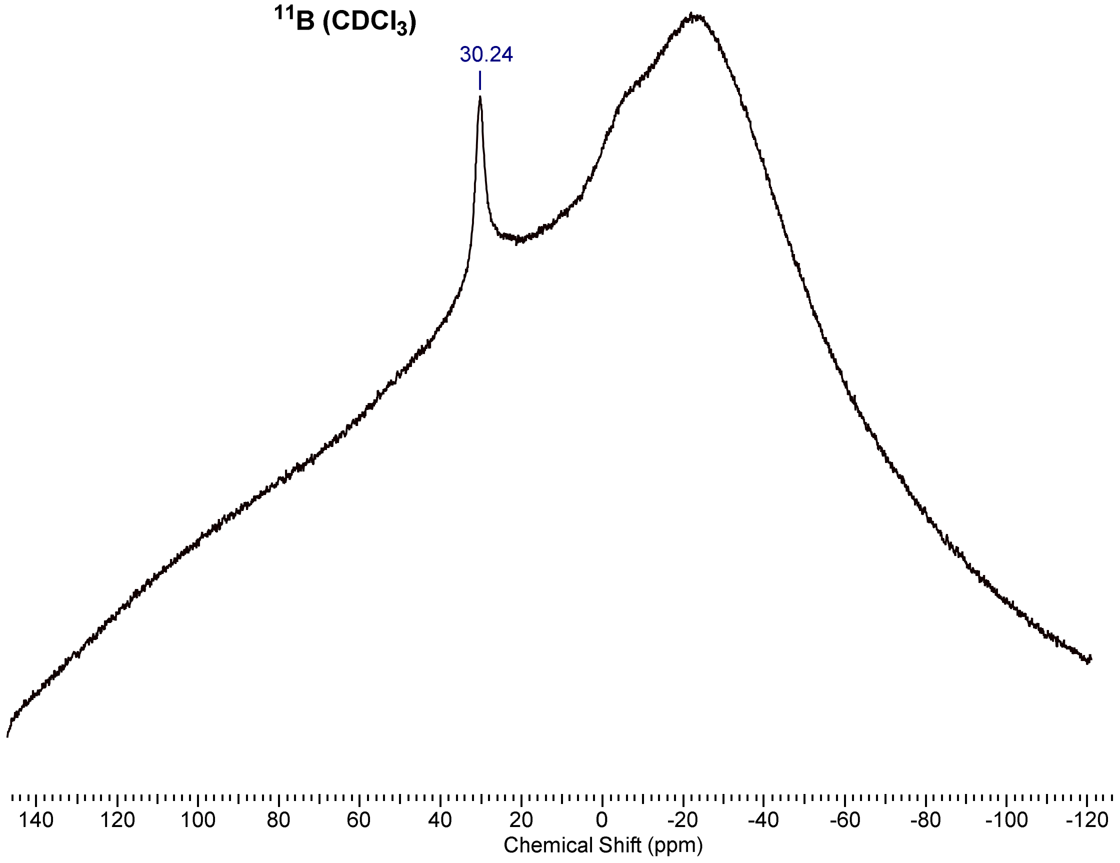

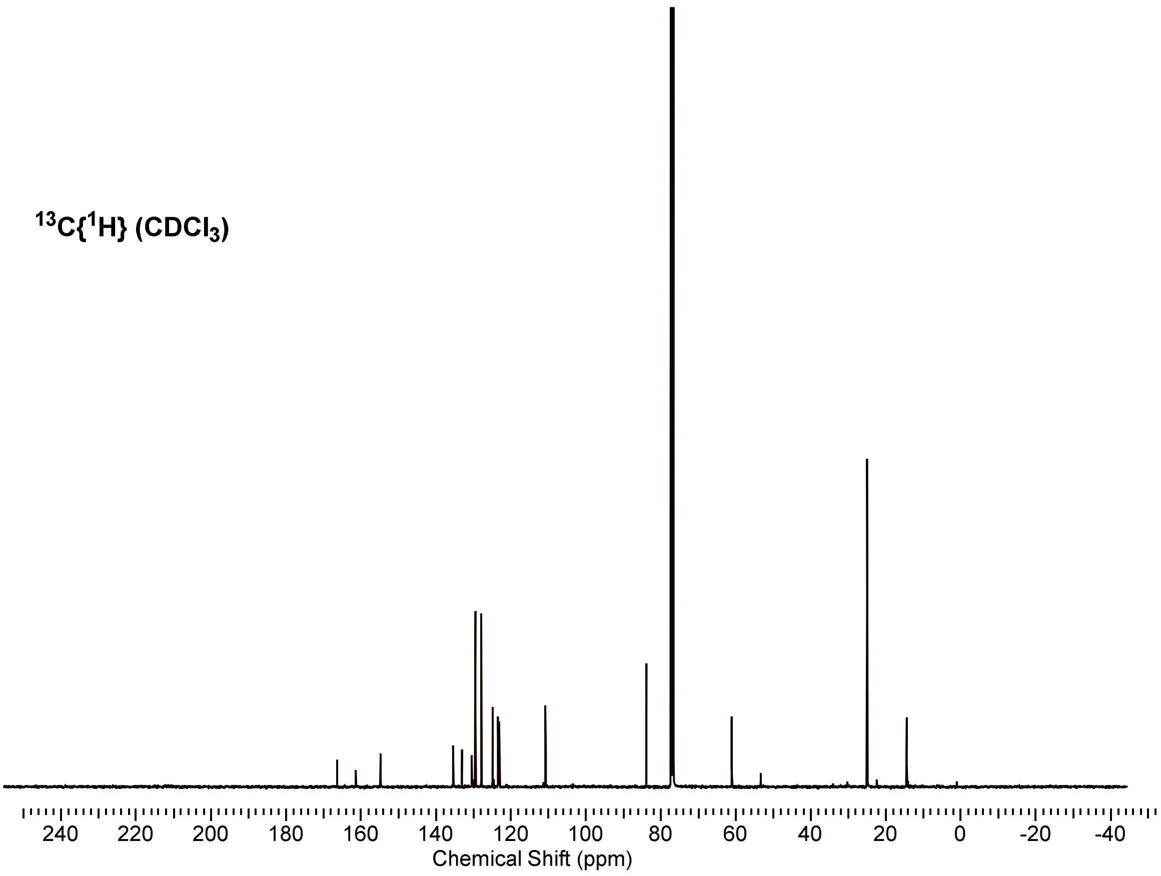

$^{13}C\{^1H\}$ ($CDCl_3$)
240 220 200 180 160 140 120 100 80 60 40 20 0 -20 -40
Chemical Shift (ppm)

Oxygen-directed *trans*-haloboration **5**

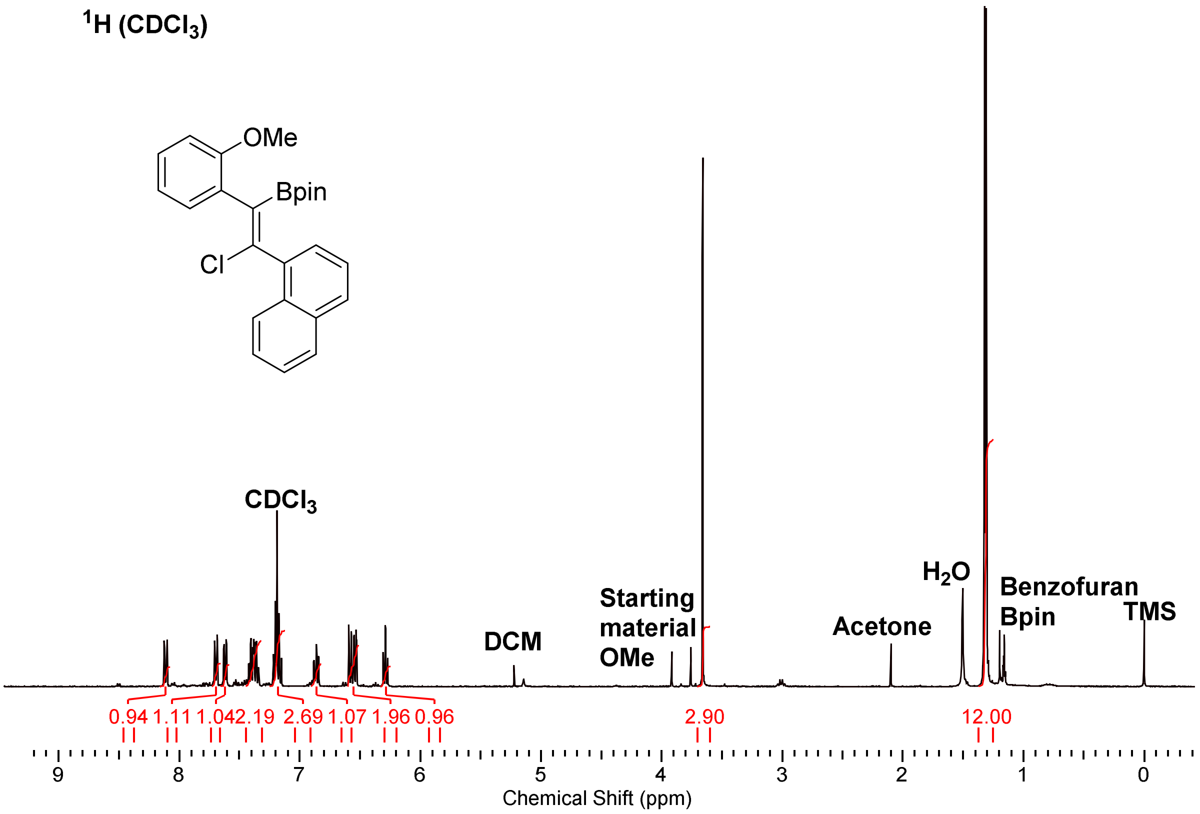

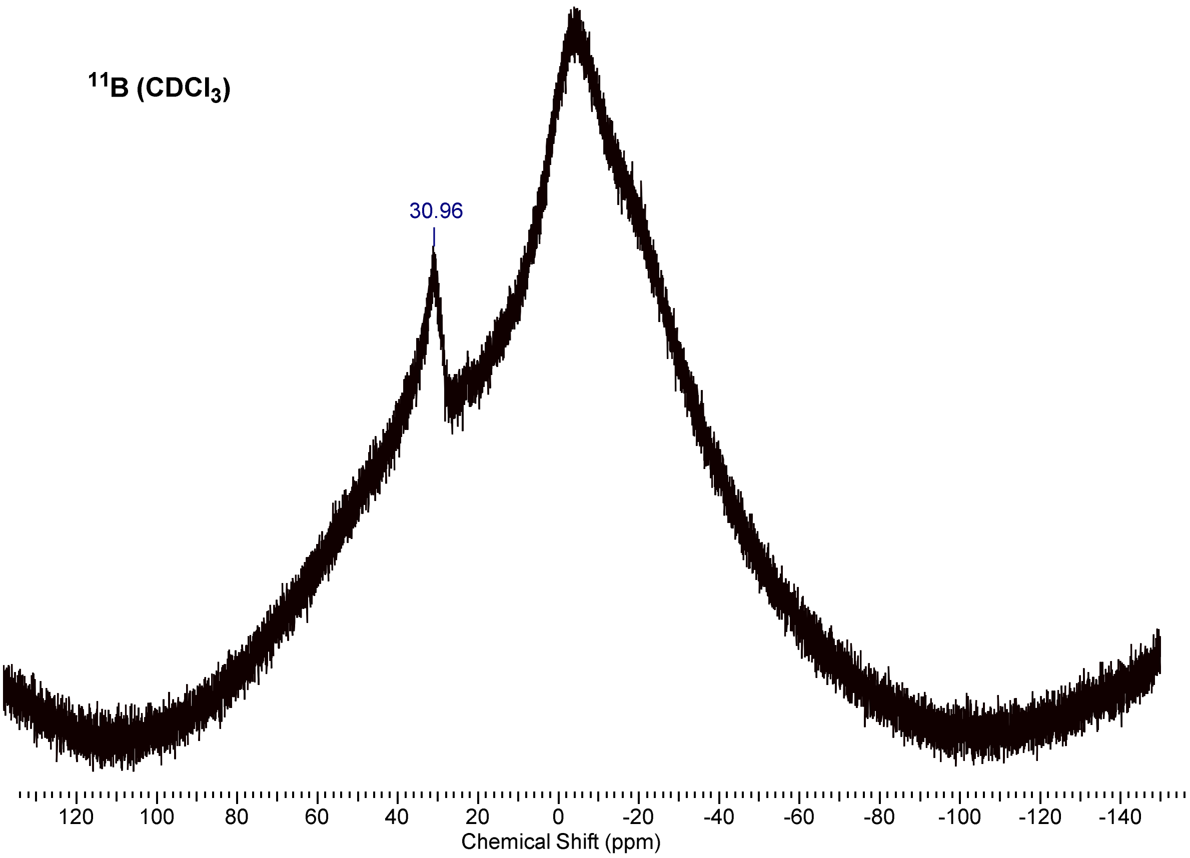

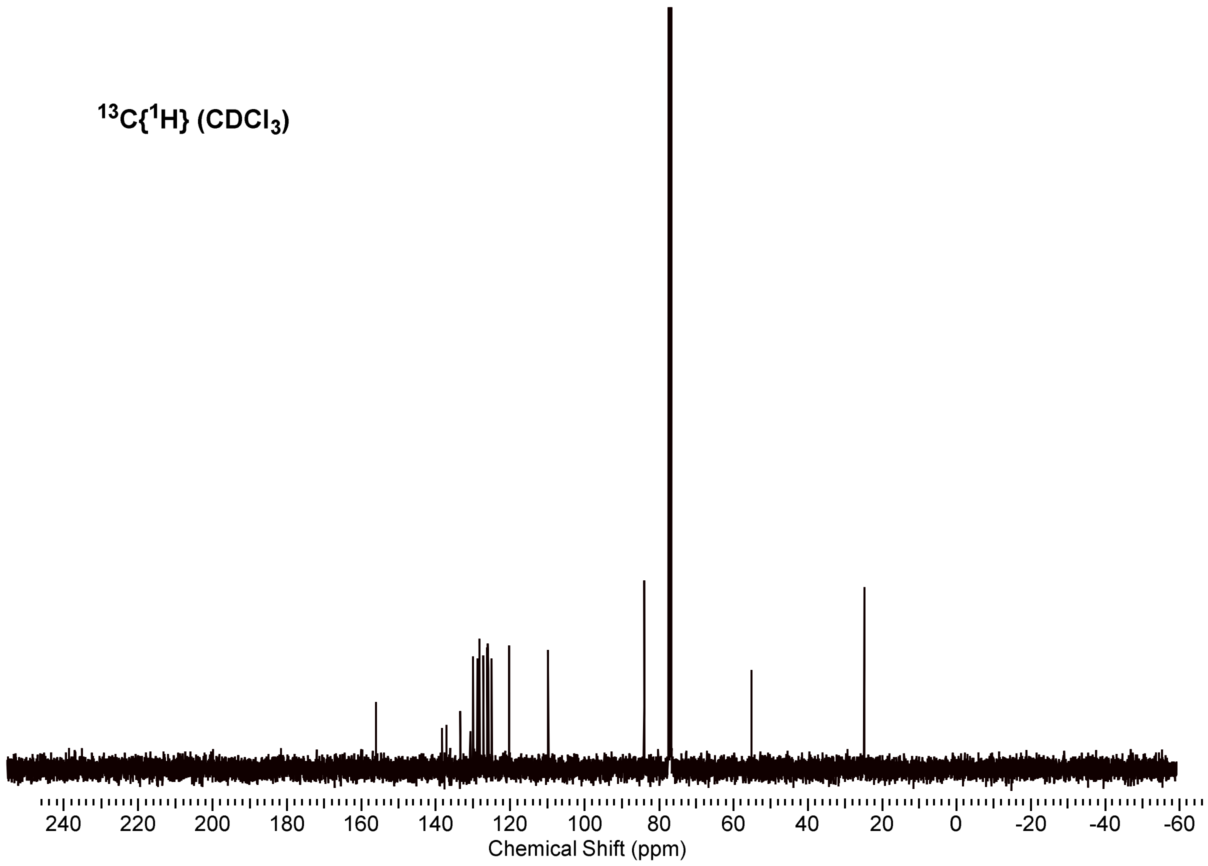

$^{13}C\{^1H\}$ ($CDCl_3$)
240 220 200 180 160 140 120 100 80 60 40 20 0 -20 -40 -60
Chemical Shift (ppm)

((2,5-dimethoxy-1,4-phenylene)bis(ethyne-2,1-diyl))dibenzene **(6)**

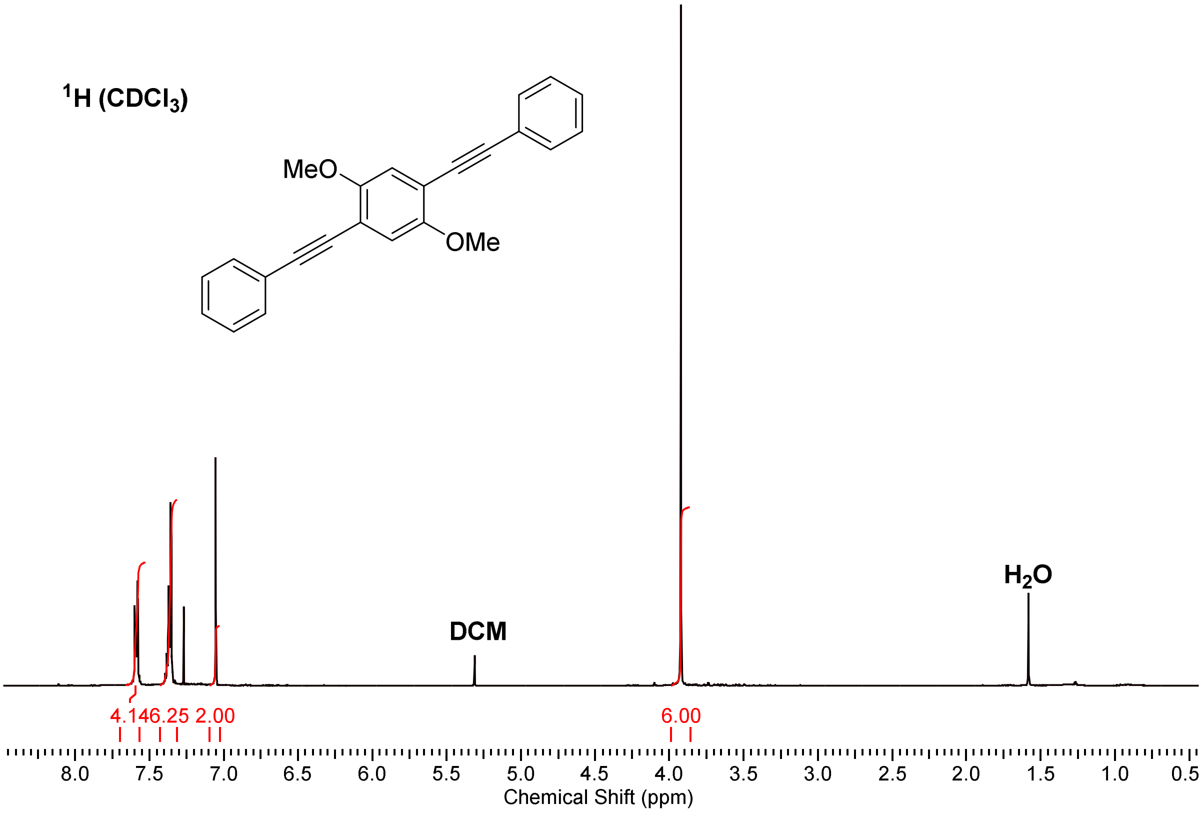

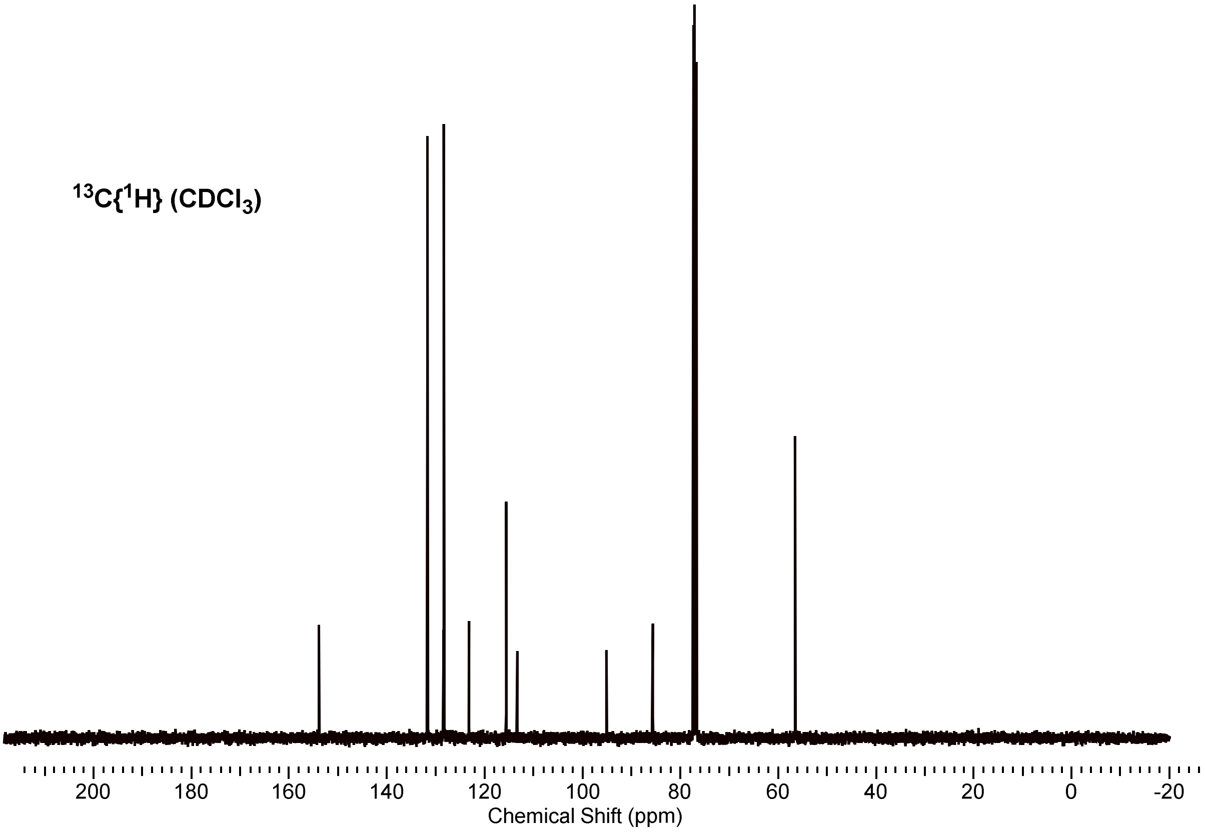

2,2’-(2,6-diphenylbenzo[1,2-b4,5-b’]difuran-3,7-diyl)bis(4,4,5,5-tetramethyl-1,3,2-dioxaborolane) **(7)**

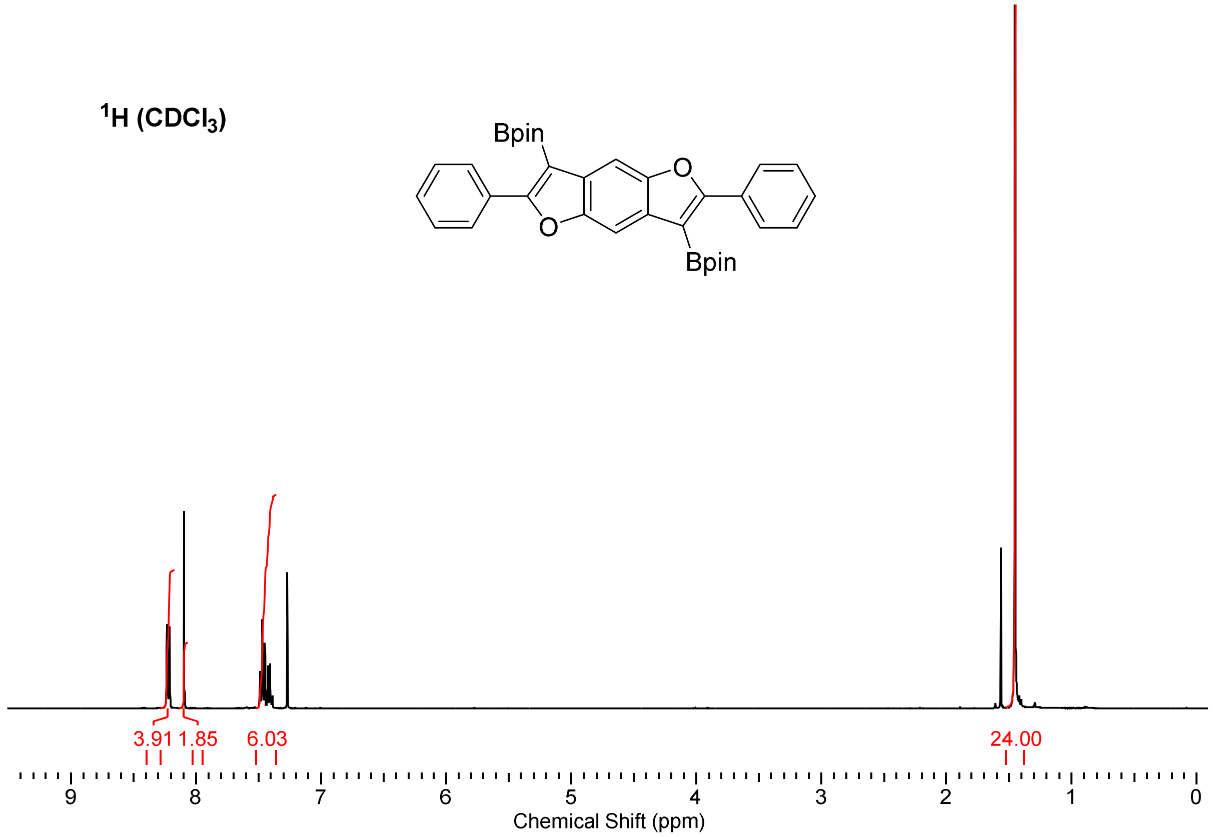

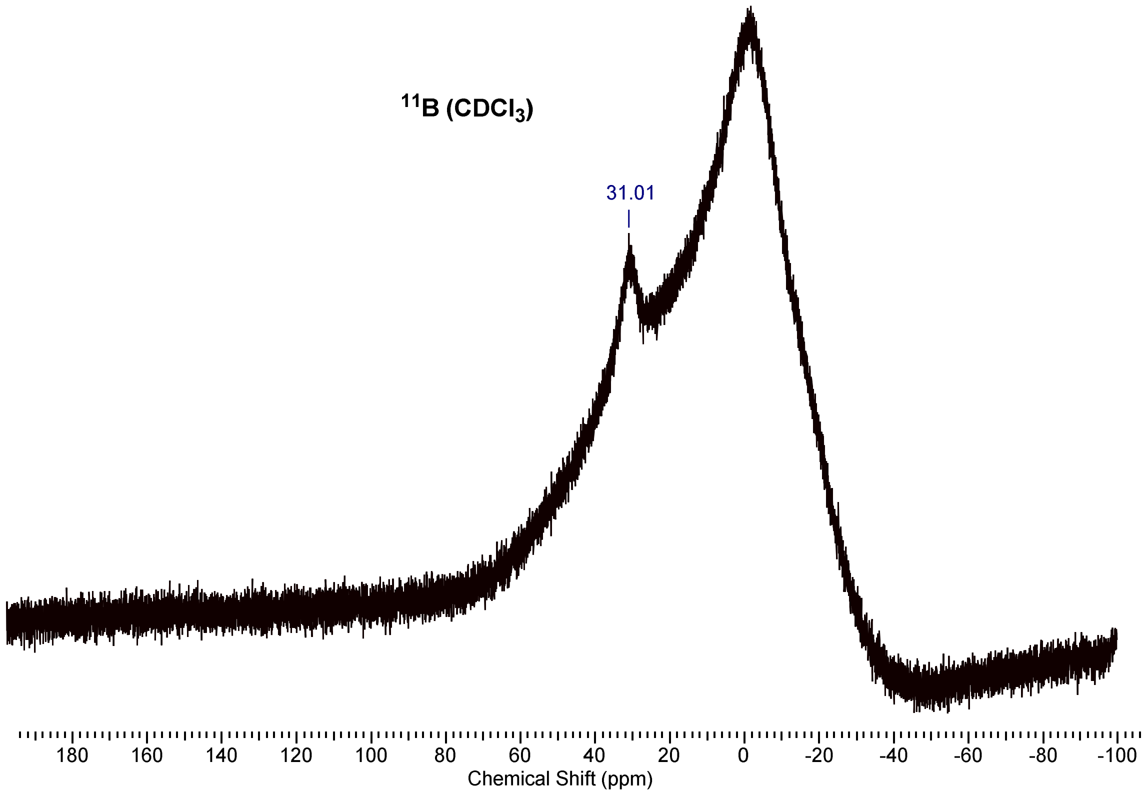

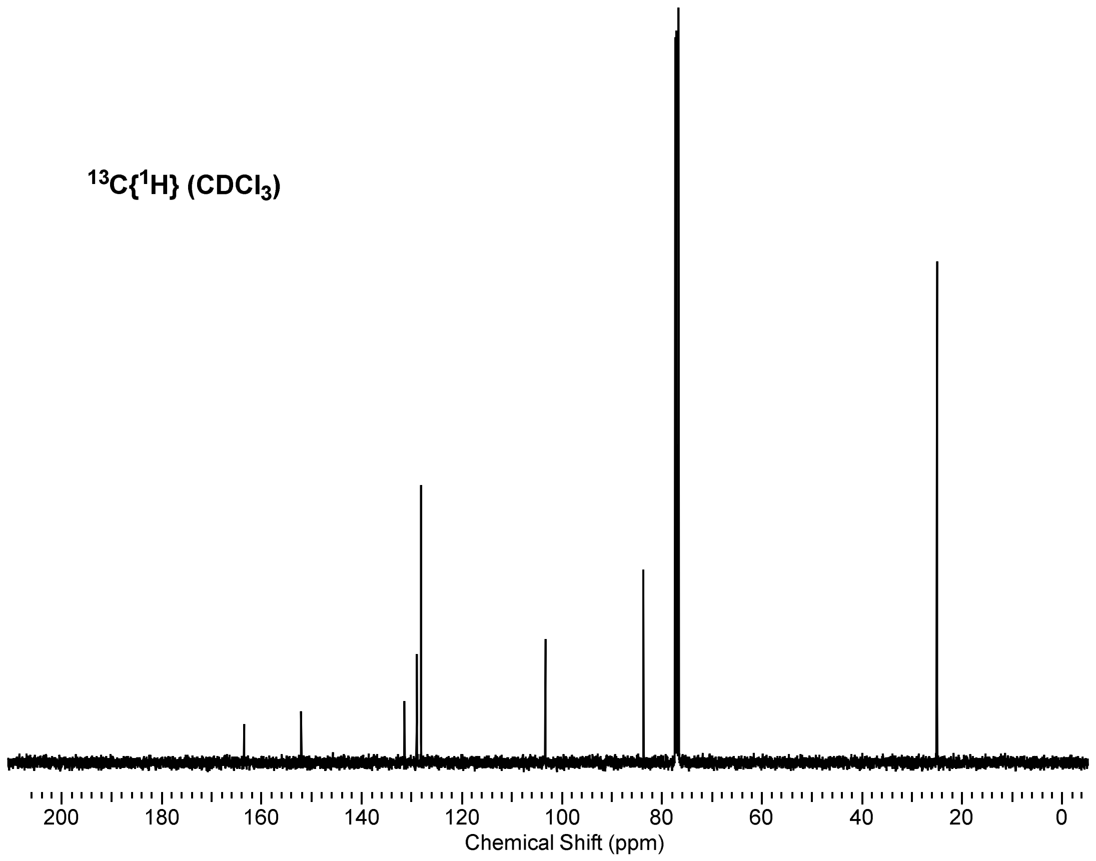

$^{13}C\{^{1}H\}$ ($CDCl_3$)
200
180
160
140
120
100
80
60
40
20
0
Chemical Shift (ppm)

5-methyl-2-phenyl-3-(p-tolyl)benzofuran **(8)**

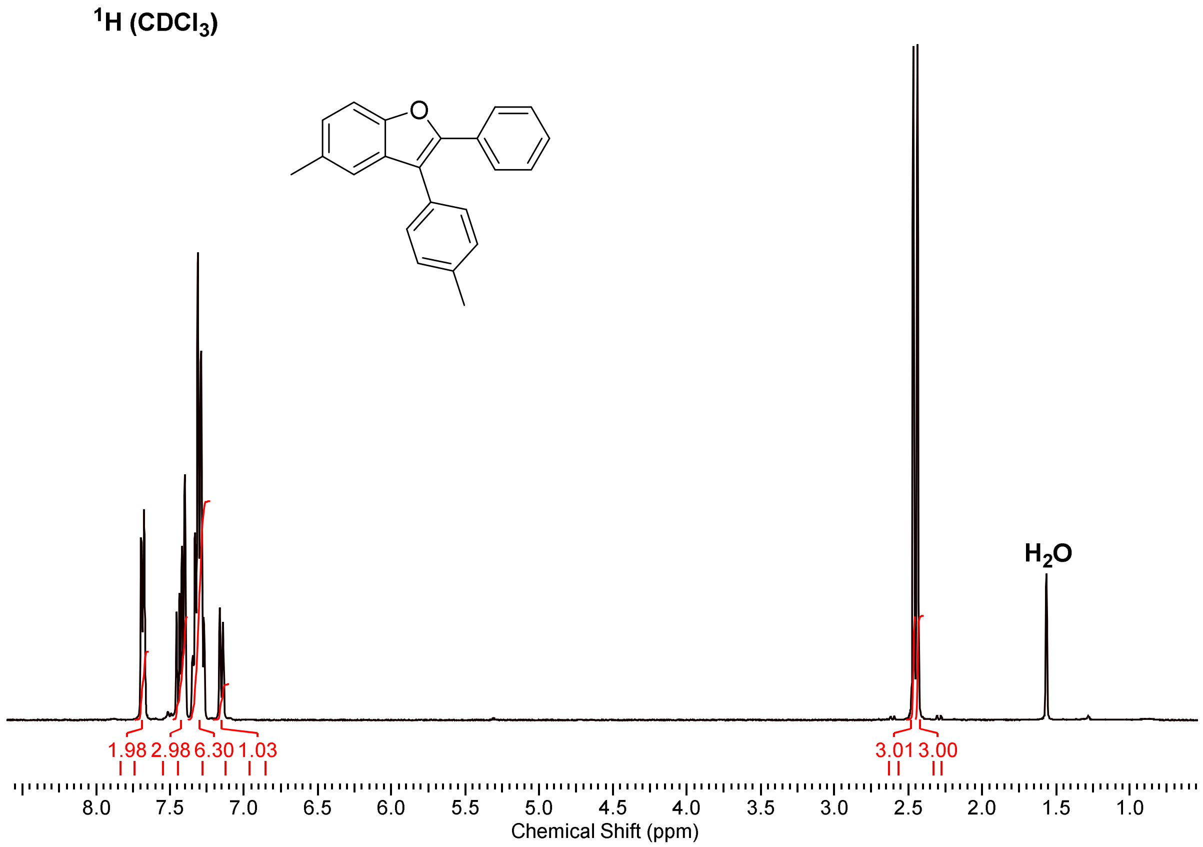

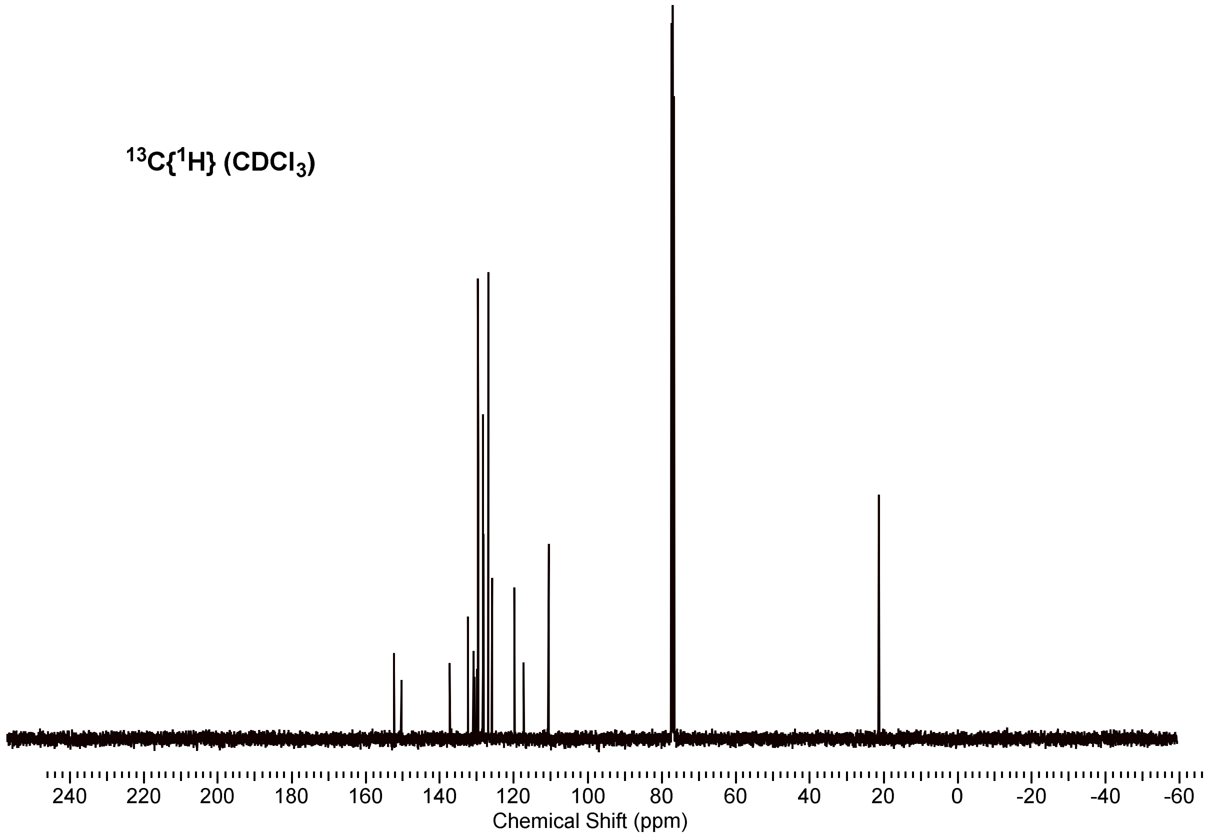

Cross coupling reaction to generate 2-methyl-3-(p-tolyl)benzofuran

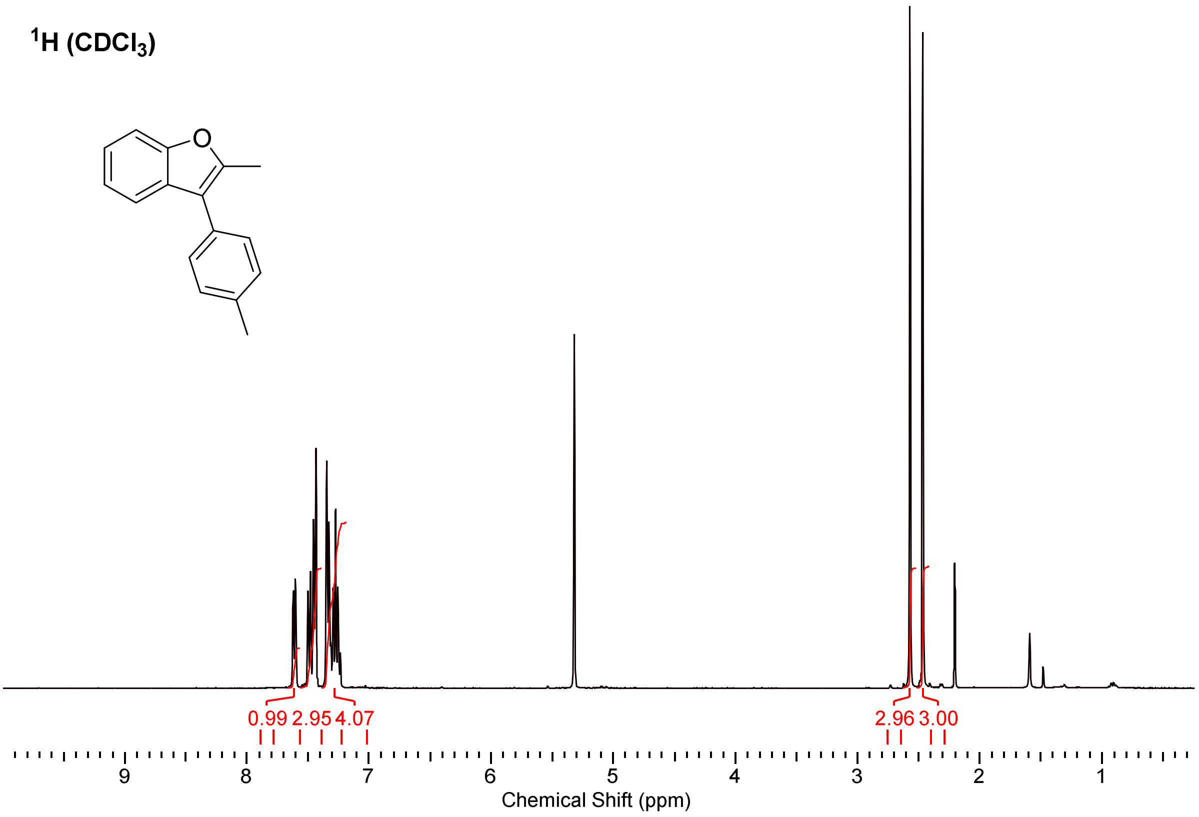

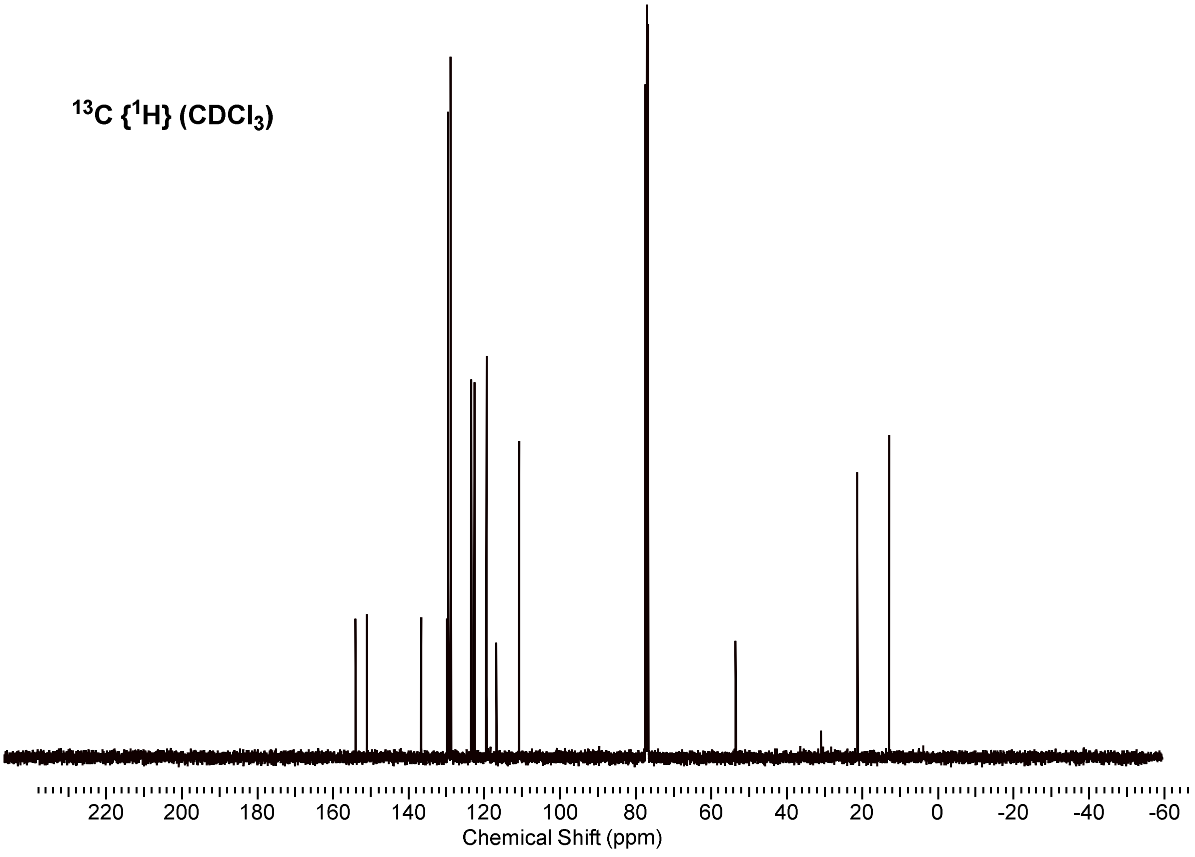

4,4,5,5-tetramethyl-2-(2-phenylbenzo[b]thiophen-3-yl)-1,3,2-dioxaborolane **(9a)**

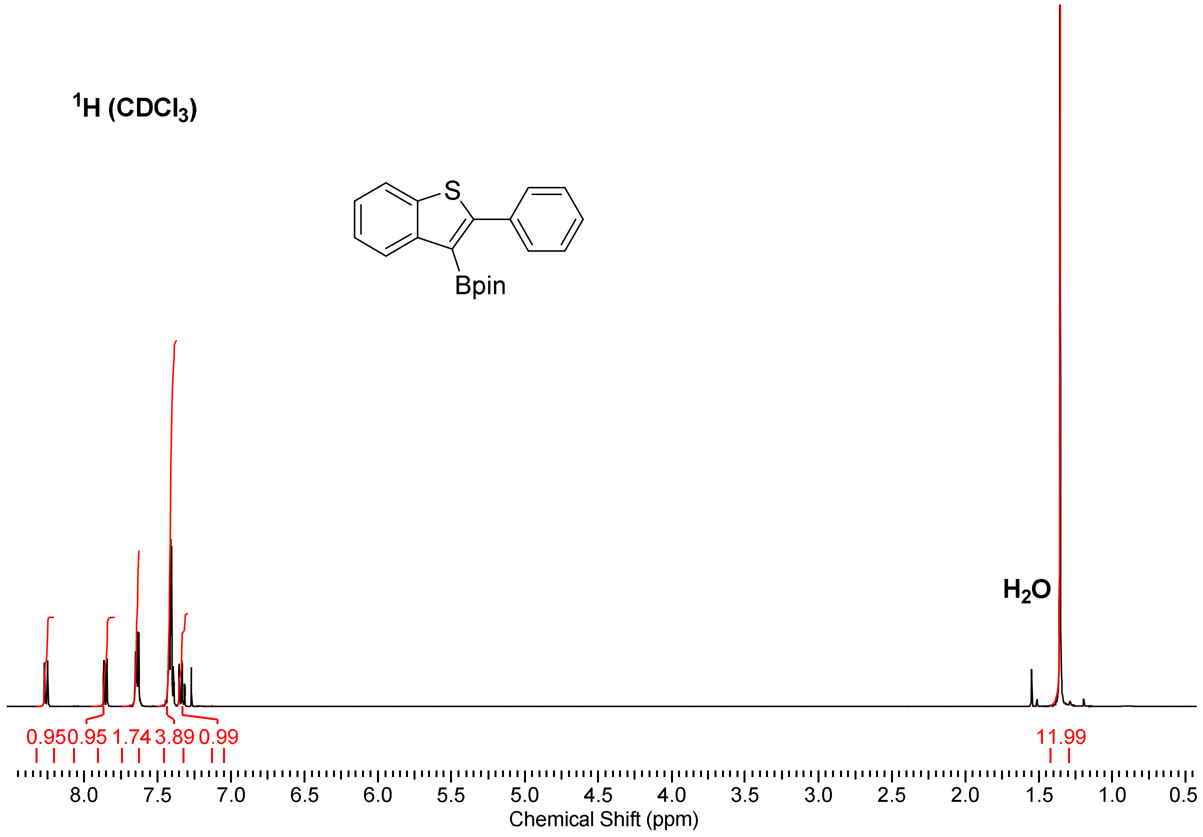

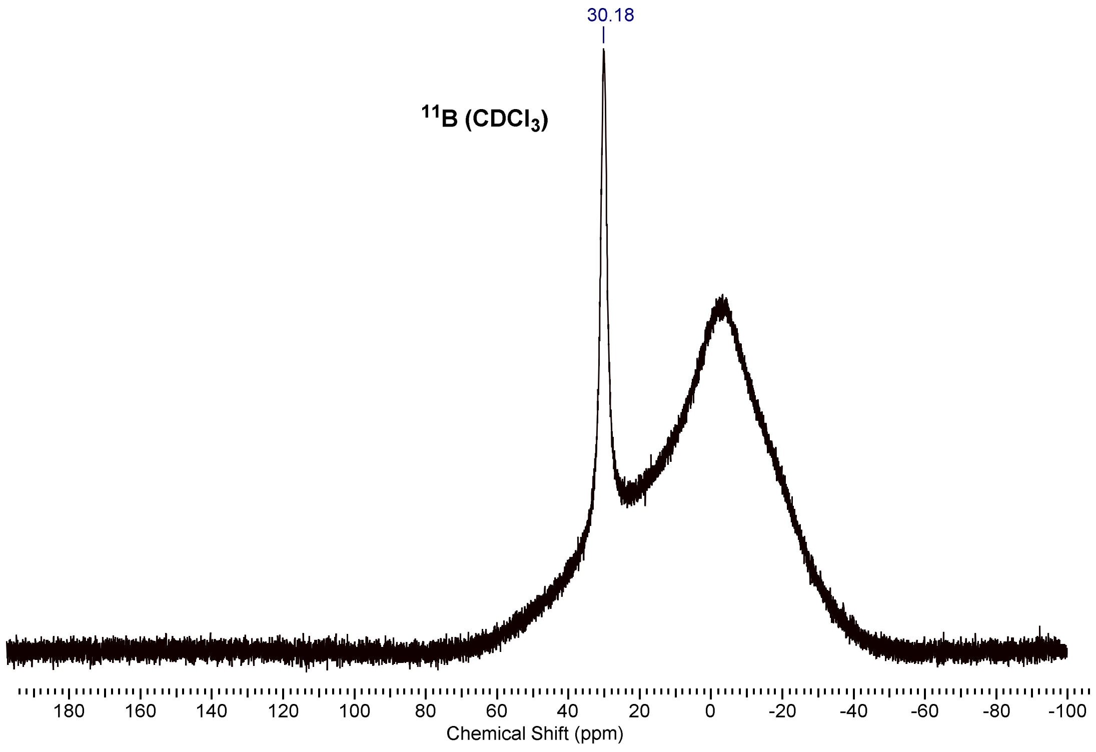

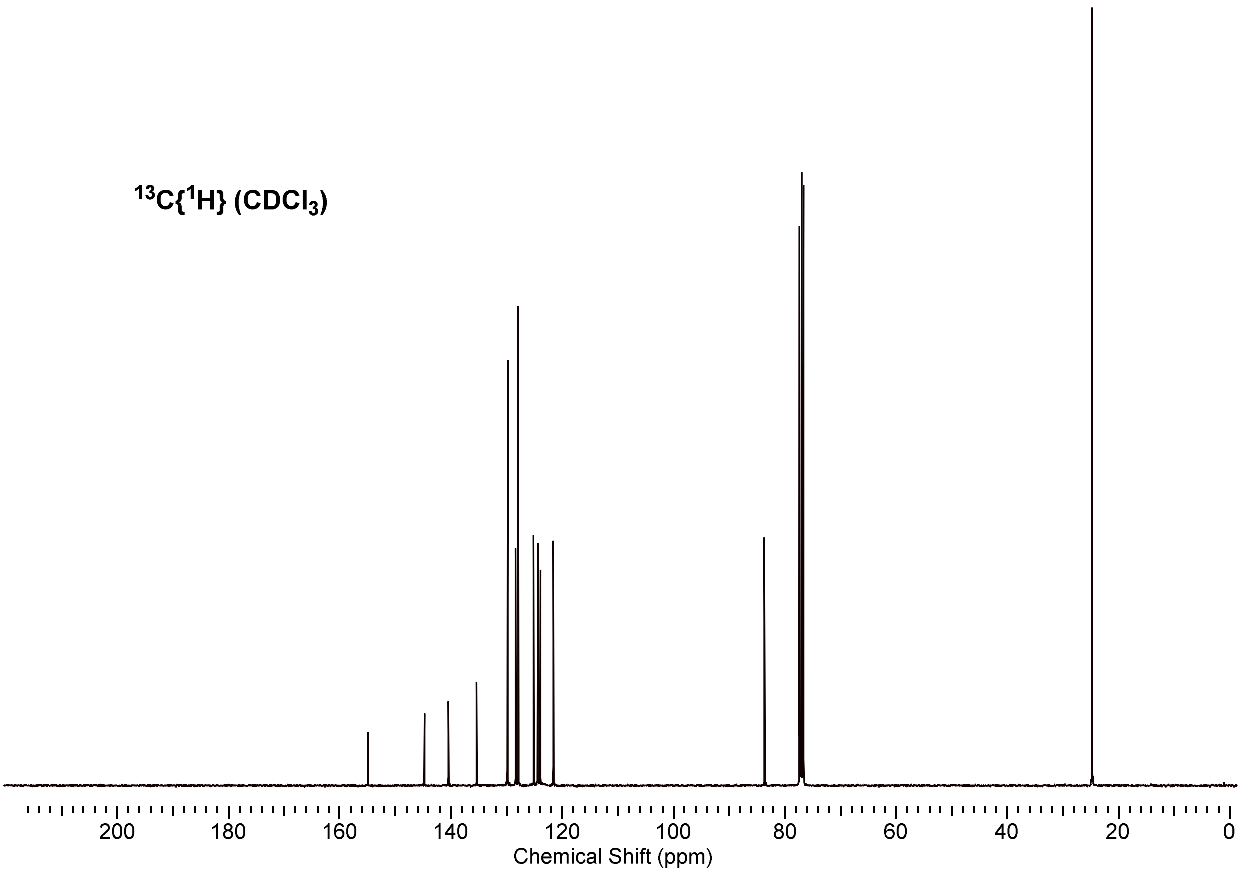

$^{13}C\{^1H\}$ ($CDCl_3$)
200
180
160
140
120
100
80
60
40
20
0
Chemical Shift (ppm)

4,4,5,5-tetramethyl-2-(2-(naphthalen-1-yl)benzo[b]thiophen-3-yl)-1,3,2-dioxaborolane **(9b)**

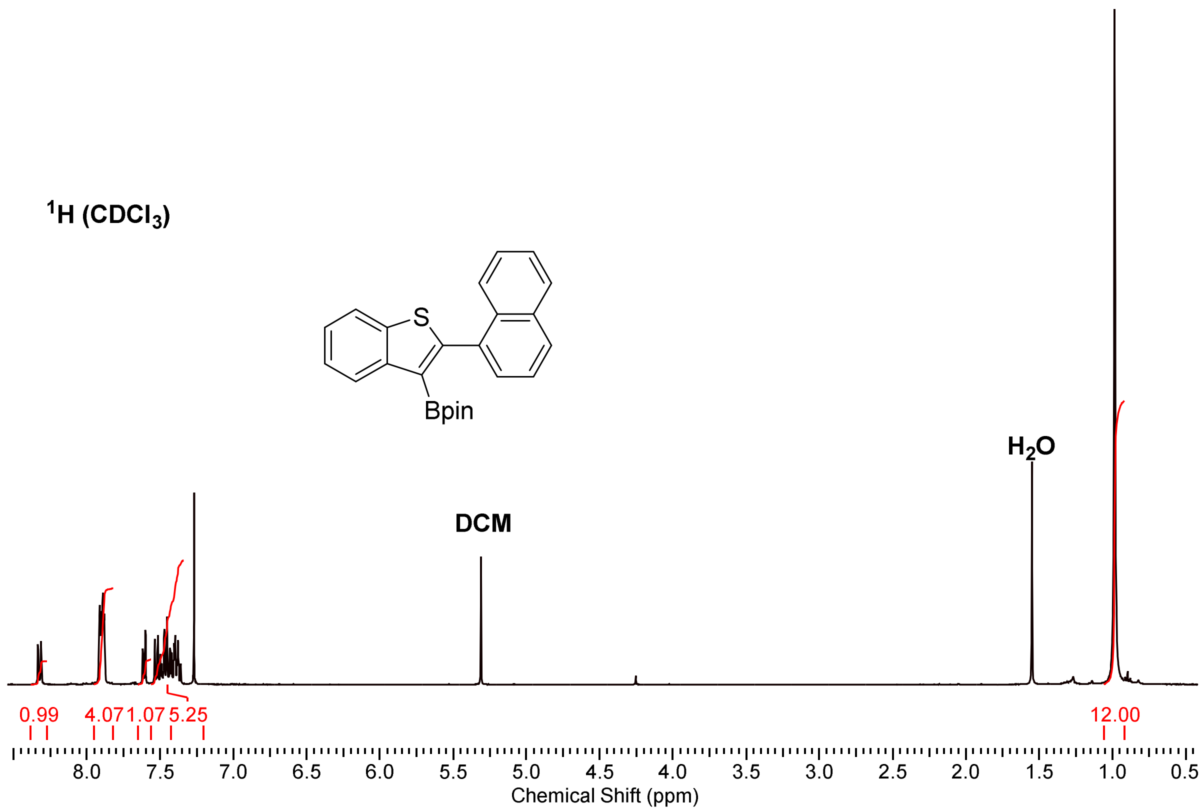

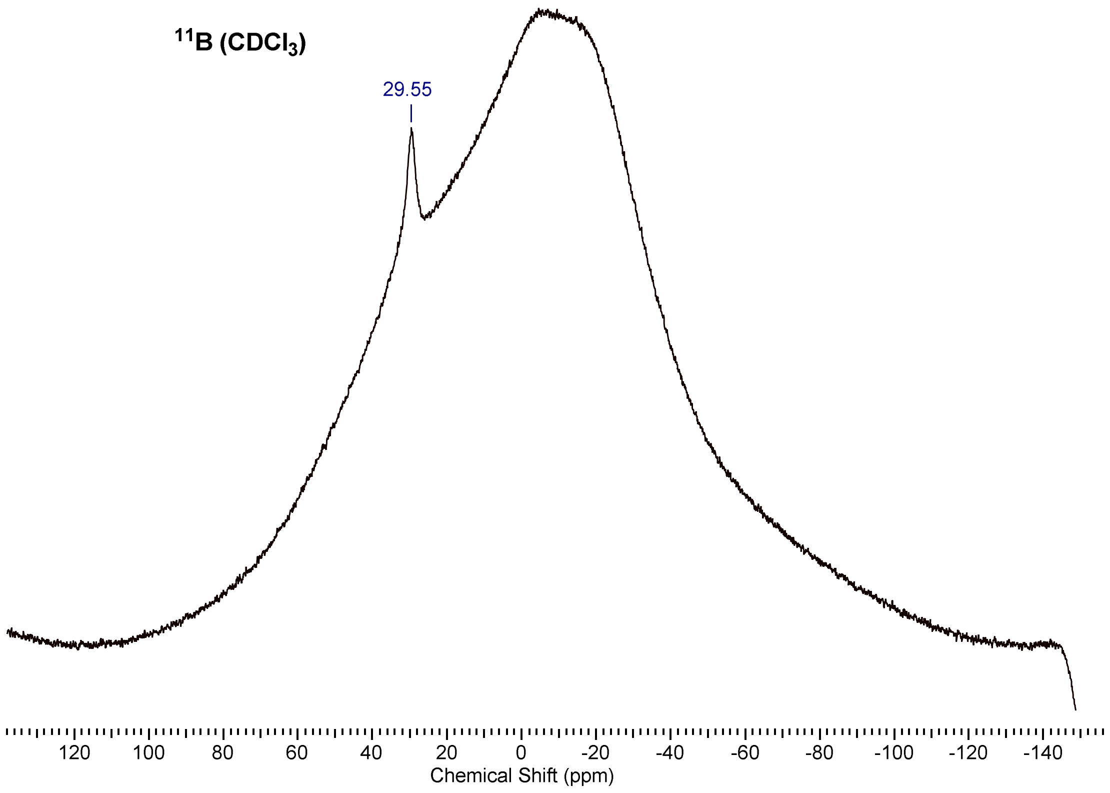

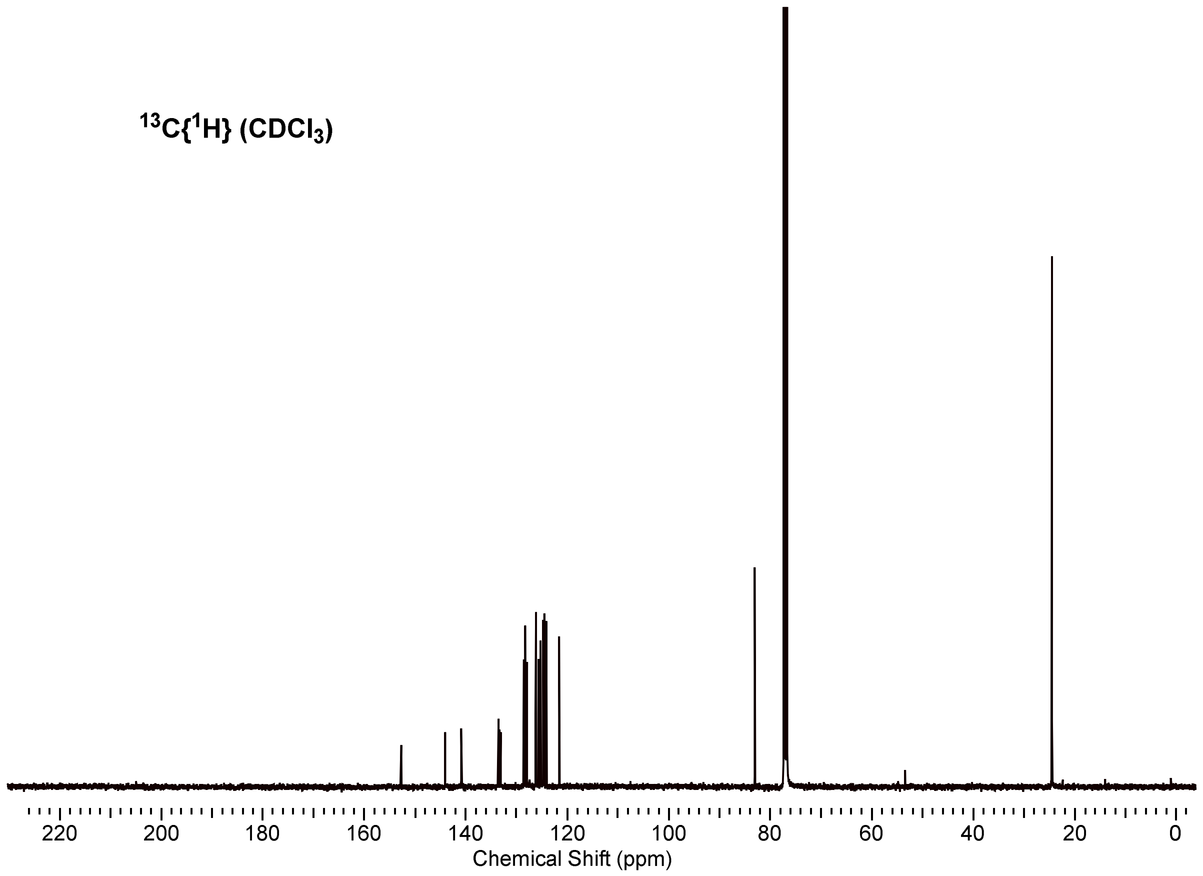
$^{13}C\{^1H\}$ ($CDCl_3$)
220
200
180
160
140
120
100
80
60
40
20
0
Chemical Shift (ppm)

2-(2-mesitylbenzo[b]thiophen-3-yl)-4,4,5,5-tetramethyl-1,3,2-dioxaborolane **(9c)**

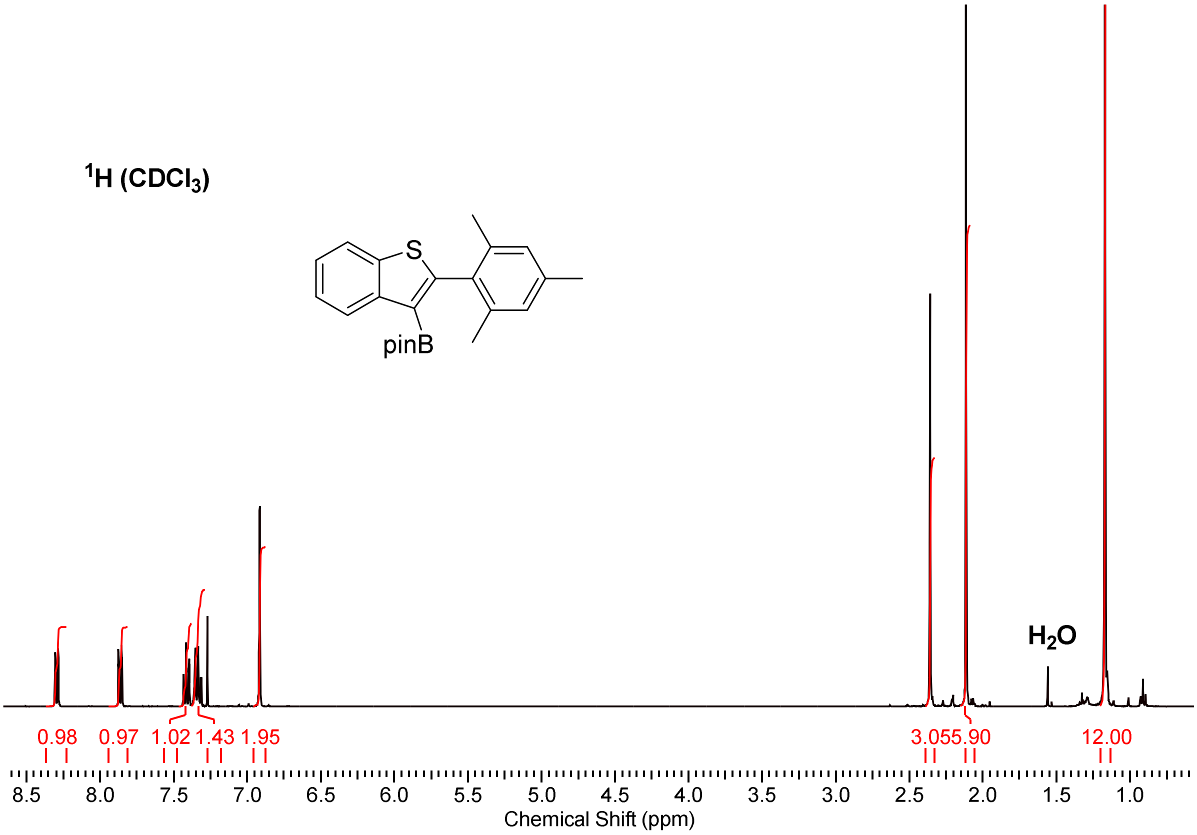

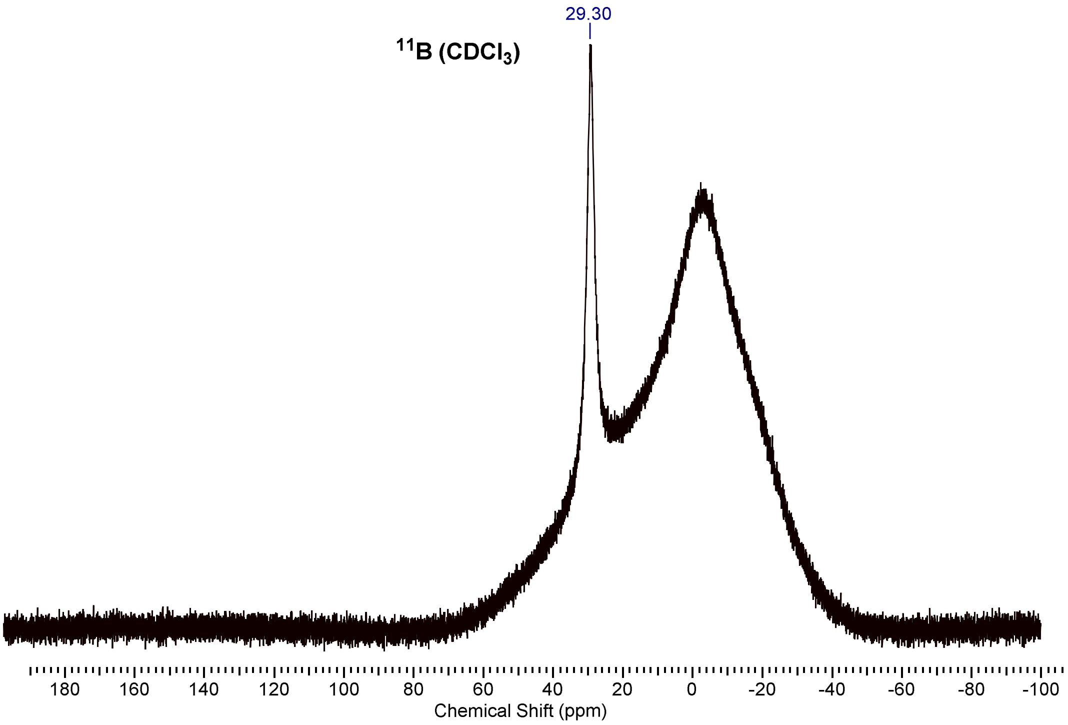

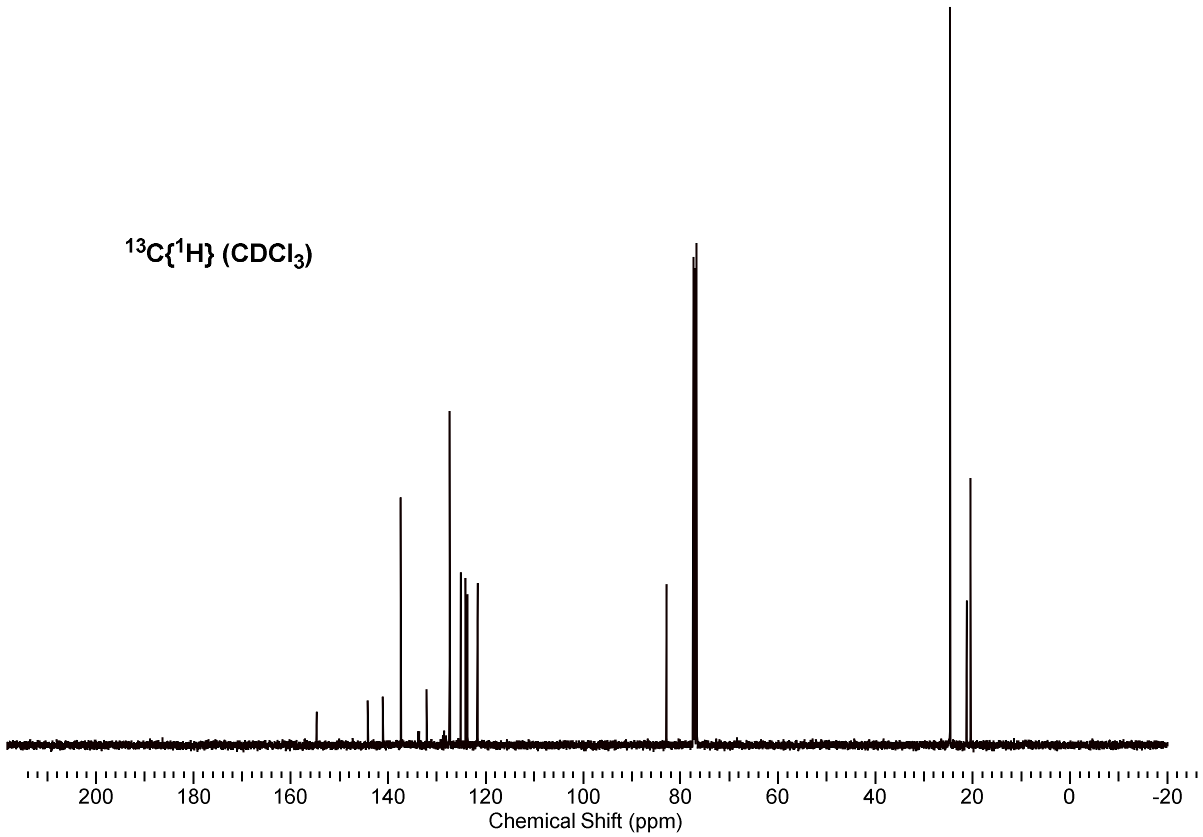
$^{13}C\{^1H\}$ (CDCl$_3$)
200
180
160
140
120
100
80
60
40
20
0
-20
Chemical Shift (ppm)

4,4,5,5-tetramethyl-2-(2-(thiophen-3-yl)benzo[b]thiophen-3-yl)-1,3,2-dioxaborolane **(9d)**

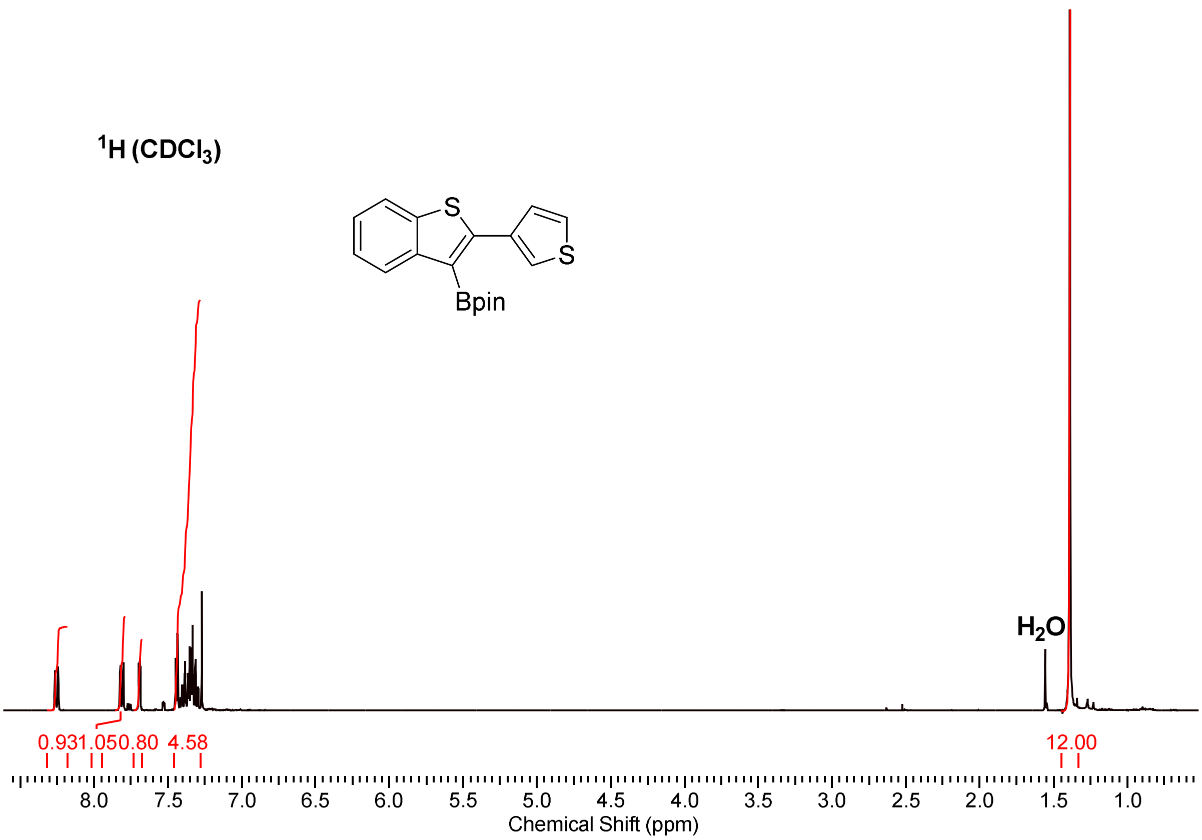

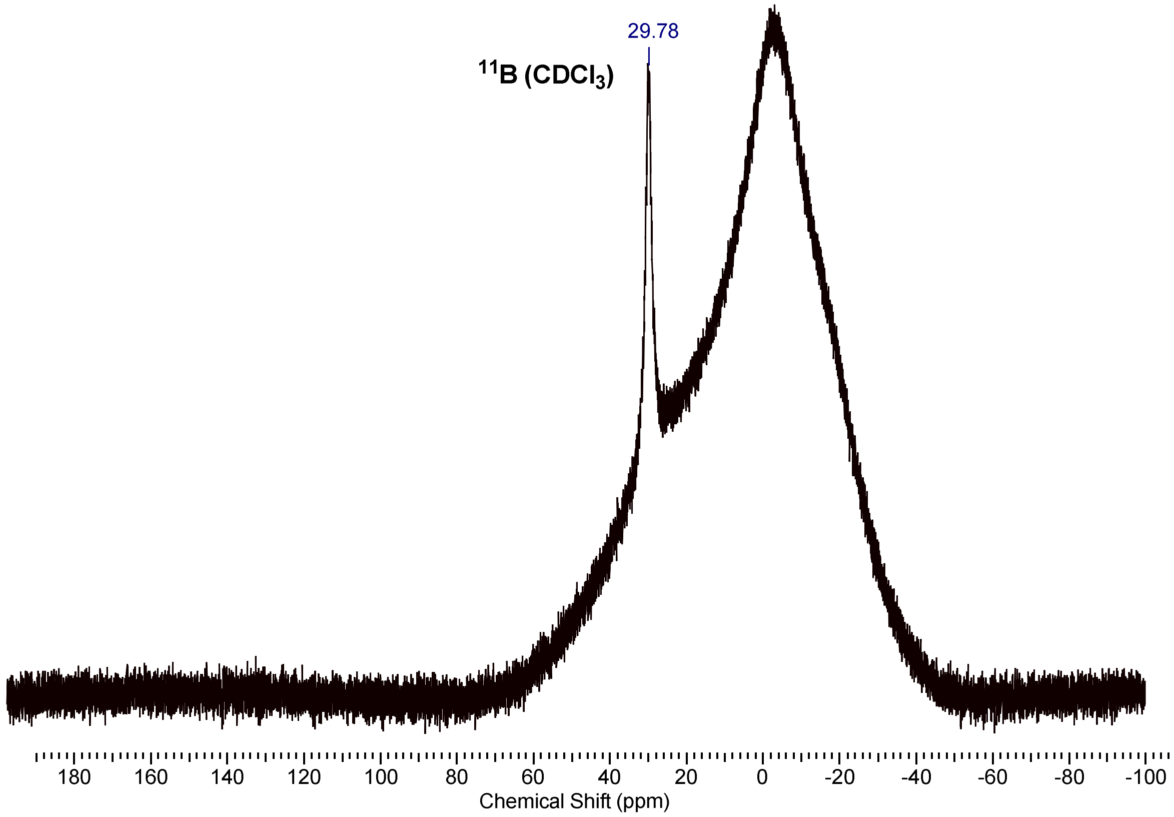
29.78
11B (CDCl3)
180 160 140 120 100 80 60 40 20 0 -20 -40 -60 -80 -100
Chemical Shift (ppm)

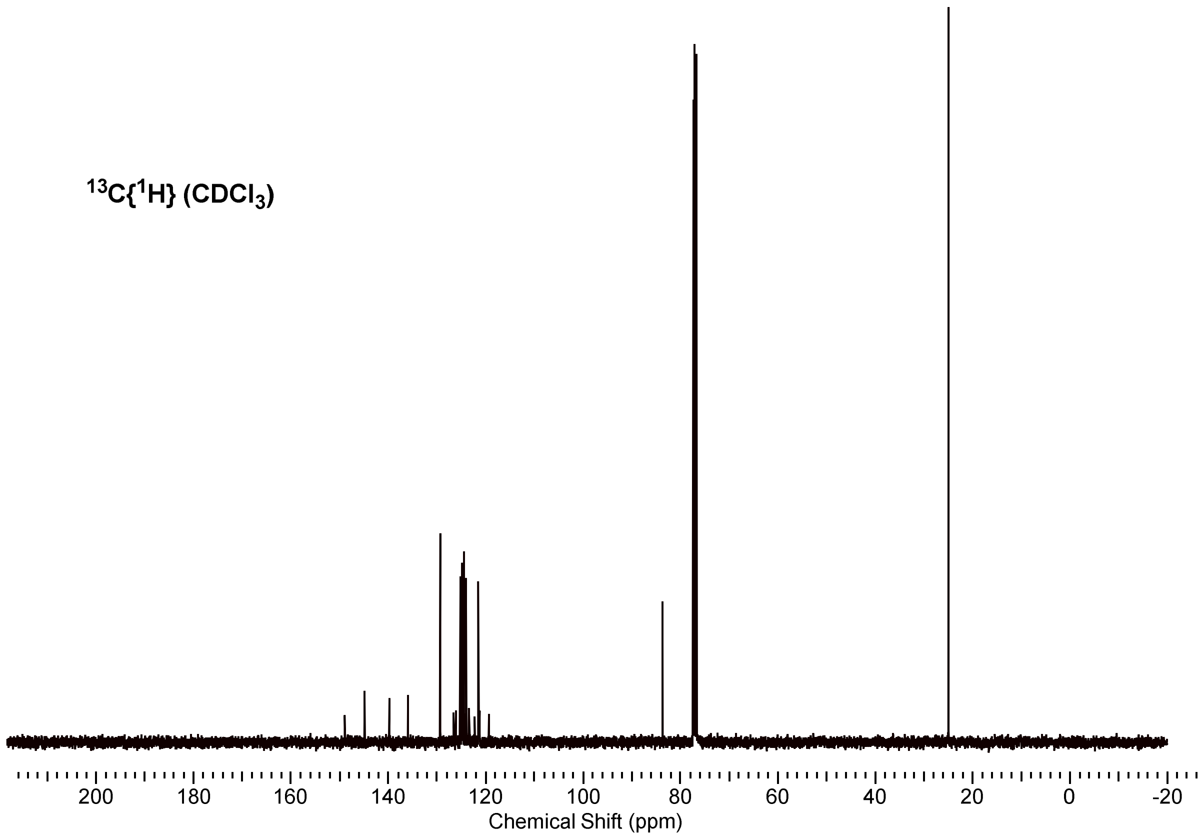
13C{1H} (CDCl3)
200 180 160 140 120 100 80 60 40 20 0 -20
Chemical Shift (ppm)

12. Crystal Data

Crystal structure of **2**, CCDC No: 1497854

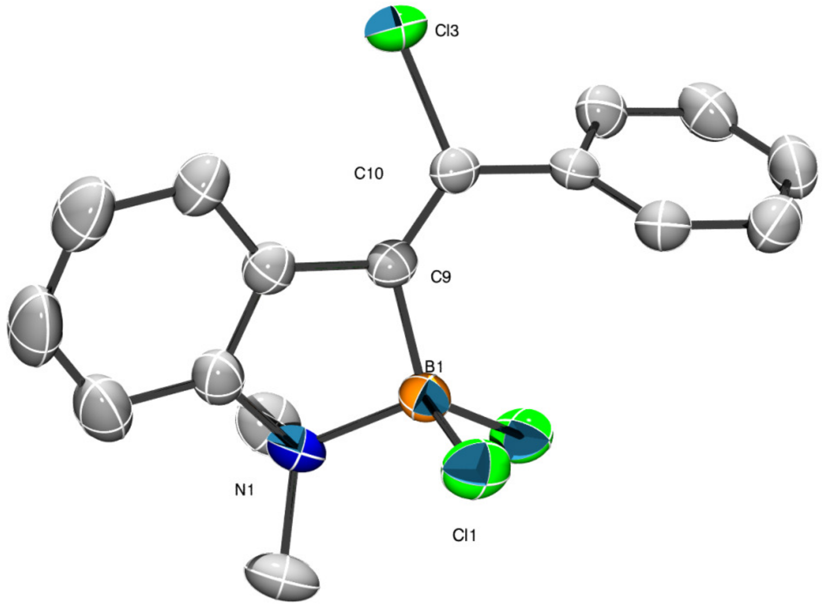

Empirical Formula	$C_{16}H_{15}BCl_3N$
FW g/mol	338.45
Crystal System and Space Group	Monoclinic, P1 21/n 1
Temperature (K)	293
a, b, c (Å)	8.9587(3), 18.6905(11), 9.9044(6)
α, β, γ (degrees)	90, 102.534(6), 90
Volume (Å^3)	1618.89(7)
Z	4
Radiation	Mo K\α, λ = 0.71703 Å
Absorption coefficient	0.557
F(000)	697.9662
Θ range (degrees)	29.32 – 3.46
Number of reflections collected	3687
Number of unique reflections	2382
Number of data/ restraints/ parameters	3687/ 0/ 191
R1 (data with [I^2> 2σ(I^2)])	0.0514
wR2 (all data)	0.0886
Goodness of fit	1.0452
Δρ maximum and minimum (e. Å^{-3})	0.3896, -0.4563

Crystal structure of **4f**, CCDC No: 1497853

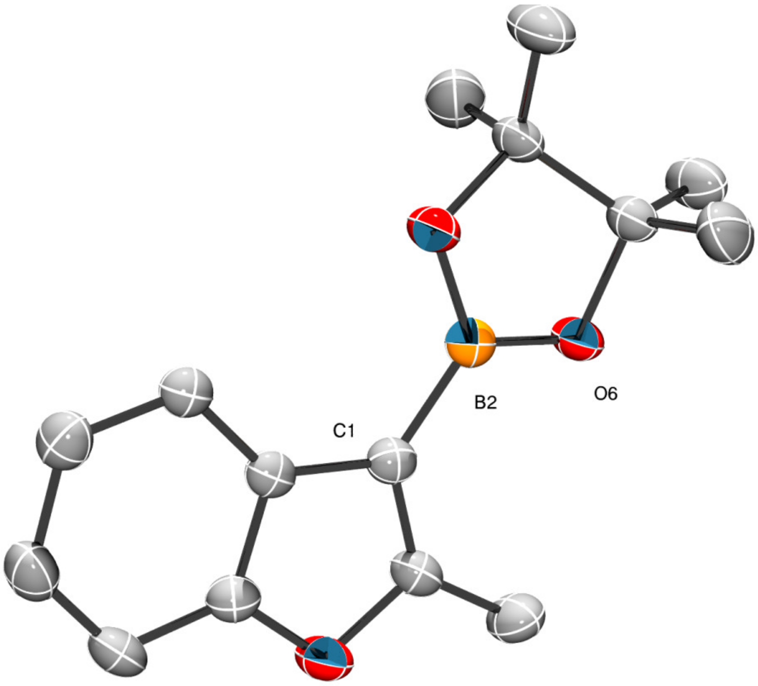

Empirical Formula	$C_{15}H_{19}BO_3$
FW g/mol	258.14
Crystal System and Space Group	Triclinic, P-1
Temperature (K)	150 K
a, b, c (Å)	9.8070(6), 11.9834(8), 13.1747(10)
α, β, γ (degrees)	110.781(6), 90.362(6), 101.733(18)
Volume (Å^3)	1412.12(18)
Z	2
Radiation	Mo K\α, λ = 0.71703 Å
Absorption coefficient	0.082
F(000)	552.2883
Θ range (degrees)	29.39 – 3.49
Number of reflections collected	6407
Number of unique reflections	3689
Number of data/ restraints/ parameters	6407/ 0/ 352
R1 (data with [I^2> 2σ(I^2)])	0.0674
wR2 (all data)	0.1300
Goodness of fit	1.0252
Δρ maximum and minimum (e. Å^{-3})	0.5223, -0.6167

13. References

1. Walkinshaw, A. J.; Xu, W.; Suero, M. G.; Gaunt, M. J. *J. Am. Chem. Soc.* **2013**, *135*, 12532
2. Yamauchi, T.; Shibahara, F.; Murai, T. Tetrahedron Lett. **2016**, *57,* 2945-2948
3. Rubin, M.; Trofimov, A.; Gevorgyan, V. *J. Am. Chem. Soc.* **2005**, *127,* 10243 – 10249
4. Gay, R. M.; Manarin, F.; Schneider, C. C.; Barancelli, D. A.; Costa, M. D.; Zeni, G. *J. Org. Chem.* **2010**, *75*, 5701 – 5706
5. Cheng, C.; Isobe, M.; Tetrahedron, **2011**, *67,* 9957-9965
6. Maji, M. S.; Murarka, S.; Studer, A., *Org.Lett.* **2010**, *12,* 3878 – 3881
7. Yue, D.; Yao, T.; Larock, R. C. *J. Org. Chem.* **2005**, *70*, 25, 10292-10296
8. Jacubert, M.; Provot, O.; Peyrat, J.; Hamze, A.; Brion, J.; Alami, M., *Tetrahedron,* **2010**, *66*, 3375-3787
9. Thomas, R.; Varghese, S.; *Journal of Materials Chemistry*, **2009**, *19*, 4401 – 4406
10. Li, X.; Liu, X.; Chen, H.; Wu, W.; Qi, C.; Jiang, H. *Angew. Chem. Int. Ed.* **2014**, *53*, 14485-14489
11. Warner, A. J., Lawson, J. R., Fasano, V., Ingleson, M. J., *Angew. Chem. Int. Ed.,* **2015**, *127,* 11397-11401
12. Paraja, M.; Prez-Aguilar, C.; Valds, C.; *Chem. Comm.,* **2015**, *51*, 90, 16241-16243
